# Supplementary material for: Liver ChREBP deficiency inhibits fructose-induced insulin resistance in pregnant mice and female offspring
Source: EMBO Rep. 2024 Mar 26;25(4):25. doi: 10.1038/s44319-024-00121-w (PMC11014959; doi:10.1038/s44319-024-00121-w)
Supplement: Supplementary file 1 — Appendix [file 44319_2024_121_MOESM1_ESM.pdf]

Appendix

Table of Contents

Appendix Table S1…………………………………………………………………………..2
Appendix Table S2…………………………………………………………………………..3
Appendix Figure S1 and Figure Legend ……………………………………………………4
Appendix Figure S2 and Figure Legend ……………………………………………………5
Appendix Figure S3 and Figure Legend ……………………………………………………6
Appendix Figure S4 and Figure Legend……………………………………………….……7
Appendix Figure S5 and Figure Legend……….……………………………………………8
Appendix Figure S6 and Figure Legend ………………………………………………..….9
Appendix Figure S7 and Figure Legend …………………………………………………..10
Appendix Figure S8 and Figure Legend …………………………………………………..11
Appendix Figure S9 and Figure Legend …………………………………………………..12
Appendix Figure S10 and Figure Legend ……………………………………………..…..13
Appendix Figure S11 and Figure Legend ……………………………..…………………..14

Appendix Table S1. Sequences of primers for qRT-PCR, PCR, and promotor analysis

Gene	Sense	anti-sense
m-*Chrebp*	5'-AGATGGAGAACCGACGTATCA-3'	5'-ACTGAGCGTGCTGACAAGTC-3'
m-*Chrebpa*	5'-CGACACTCACCCACCTCTTC-3'	5'-TTGTTCAGCCGGATCTTGTC-3'
m-*Chrebpb*	5'-TCTGCAGATCGCGTGGAG-3'	5'-CTTGTCCCGGCATAGCAAC-3'
m-*Apob*	5'-AAGCACCTCCGAAAGTACGTG-3'	5'-CTCCAGCTCTACCTTACAGTTGA-3'
m-*Mttp*	5'-CTGGATCTCCATATTGGCCT-3'	5'-TGATGTCCAAAATGCTGTCG-3'
m-*Abca1*	5'-CCCAGAGCAAAAAGCGACTC-3'	5'-GGTCATCATCACTTTGGTCCTTG-3'
m-*Abcg1*	5'-GCCTACTACCTGGCAAAGACC-3'	5'-GAACAGCACAAAACGCACAG-3'
m-*β-actin*	5'-ATGGAGGGGAATACAGCCC-3'	5'-TTCTTTGCAGCTCCTTCGTT-3'
m-*Star*	5'-ATGTTCCTCGCTACGTTCAAG-3'	5'-CCCAGTGCTCTCCAGTTGAG-3'
m-*Cyp11a1*	5'-AGGTCCTTCAATGAGATCCCTT-3'	5'-TCCCTGTAAATGGGGCCATAC-3'
m-*Hsd3b*	5'-GGTTTTTGGGGCAGAGGATCA-3'	5'-GGTACTGGGTGTCAAGAATGTCT-3'
m-*Mln64*	5'-GTGACTTGGAGCGCAGTTTG-3'	5'-GCCAGTGTTGGTATTTAGCTCG-3'
h-*Chrebp*	5'-AGAACCGGCGTATCACACAC-3'	5'-GTGCTCACGAGCCCATGAA-3'
h-*Chrebpa*	5'-AGTGCTTGAGCCTGGCCTAC-3'	5'-TTGTTCAGGCGGATCTTGTC-3'
h-*Chrebpb*	5'-AGCGGATTCCAGGTGAGG-3'	5'-TTGTTCAGGCGGATCTTGTC-3'
h-*β-actin*	5'-CTGGAACGGTGAAGGTGACA-3'	5'-AAGGGACTTCCTGTAACAATGCA-3'
Chrebp$^{\text{flox/flox}}$ 5'arm	ATAGGACAGGAAGGTGTCCAAGACC	GGCCTTGAAATCAACTCTGTAGACC
Chrebp$^{\text{flox/flox}}$ 3'arm	TCCAGTGGAAAAGTGTCTGCCTAG	AGGATCTCTGTGTTCGAGGCCA
h*Chrebp* promoter-*PPRE* mutation	ggttggaggtgaCTACAGTTCCAGTggtgagg	cctcaccACTGGAACTGTAGtcacctccaacc

m, mus musculus; h, homo sapiens; ChREBP, carbohydrate response element-binding protein; Apob, apolipoprotein B; Mttp, microsomal triglyceride transfer protein; Abca1, ATP binding cassette subfamily A member 1; Abcg1, ATP binding cassette subfamily G member 1. Star, steroidogenic acute regulatory protein; Cyp11a1, cholesterol side-chain cleavage; Hsd3b, 3-β-hydroxysteroid dehydrogenase; Mln64, StAR-related lipid-transfer protein 3. The capital letters are mutated nucleotides in the *PPRE* of the human *ChREBP* promoter.

Appendix Table S2. Embryonic absorption rate in each group

	Absorbed embryos (pcs)	Survived embryos (pcs)	Embryo absorption rate (%)
Chrebp$^{flox/flox}$-NC	0	7±1	0%
Chrebp$^{flox/flox}$-HFrD	1.8±1***	5.2±1*	25%±8***
Chrebp KO-NC	3±3*	6±3	30%±34*
Chrebp KO-HFrD	2±2	5.7±3	28%±35

*, P<0.05, **, P<0.01, ***, P<0.001 vs *Chrebp*$^{flox/flox}$-NC. n = 6~8. Statistical comparisons are between genotypes at similar embryonic day.

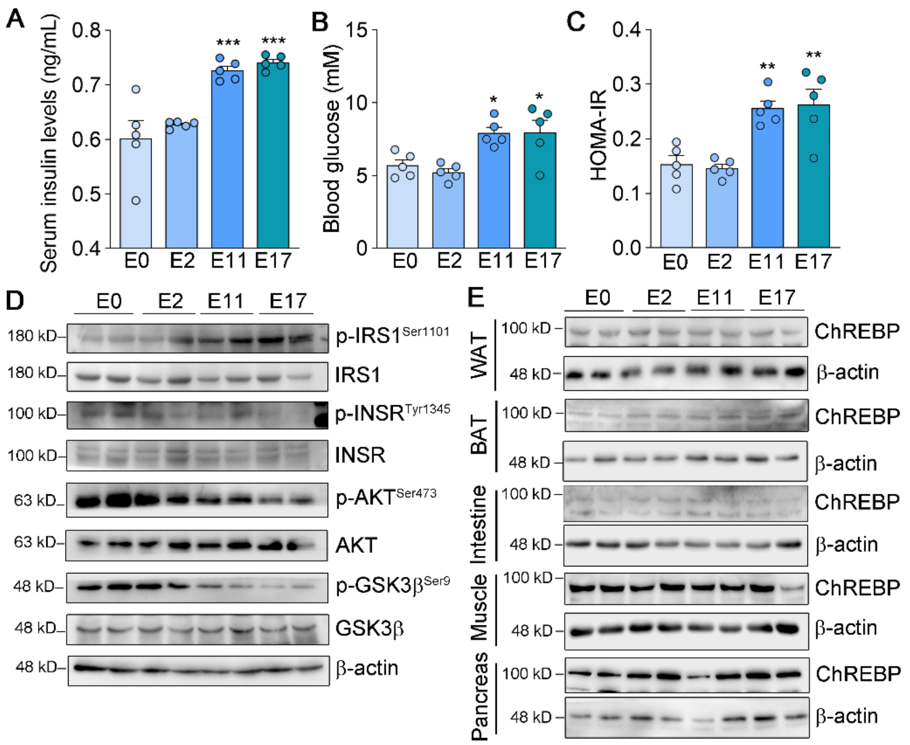

Appendix Figure S1. Increased IR in normal pregnancy. The mice in **Figure 1A** were used to complete following assays: (**A-C**) Determination of serum insulin (**A**), blood glucose (**B**) and HOMA-IR (**C**) levels. (**D**) Expression of protein related to insulin signaling pathway in mouse liver was determined by Western blot. (**E**) Different tissue samples were collected and ChREBP protein levels were determined by Western blot. n = 5.

Data information: All graphs are represented as Mean ± SEM, n: biological replicates. Owo-way ANOVA followed by Tukey's multiple comparisons test were used. *P<0.05, **P<0.01, ***P<0.001 vs. E0 group.

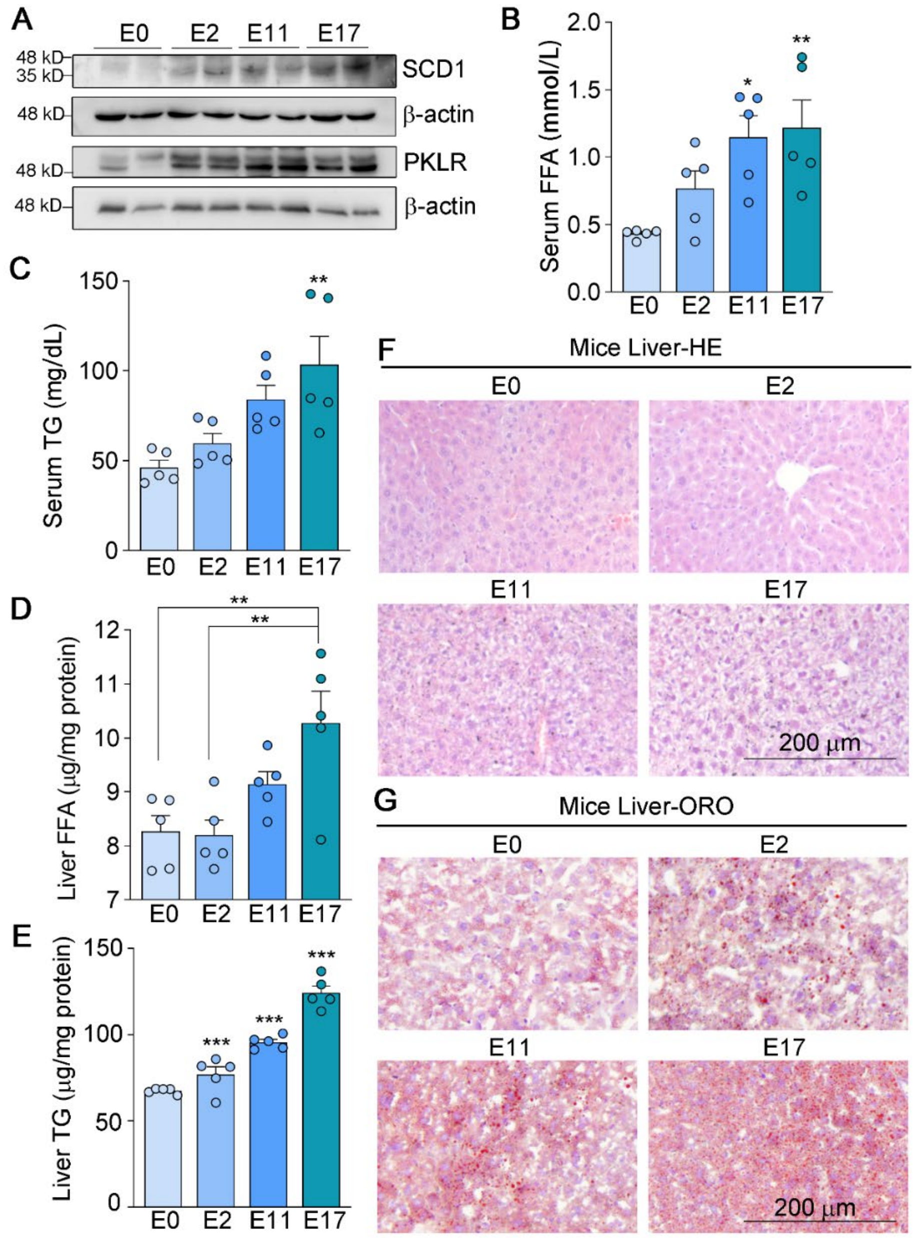

Appendix Figure S2. Increased lipids accumulation in normal pregnancy. The mice in **Figure 1A** were used to complete following assays: (**A**) Expression of protein related to downstream targets protein of ChREBP in mouse liver was determined by Western blot. (**B**, **C**) Free fatty acid (FFA) and triglyceride (TG) quantitative analysis in serum. (**D**, **E**) FFA and TG quantitative analysis in liver lipid extract. (**F, G**) HE staining (**F**) and Oil Red O (ORO) (**G**) staining of liver sections. n = 5.

Data information: All graphs are represented as Mean ± SEM, n: biological replicates. Owo-way ANOVA followed by Tukey's multiple comparisons test were used. *P<0.05, **P<0.01, ***P<0.001 vs. E0 or indicated group.

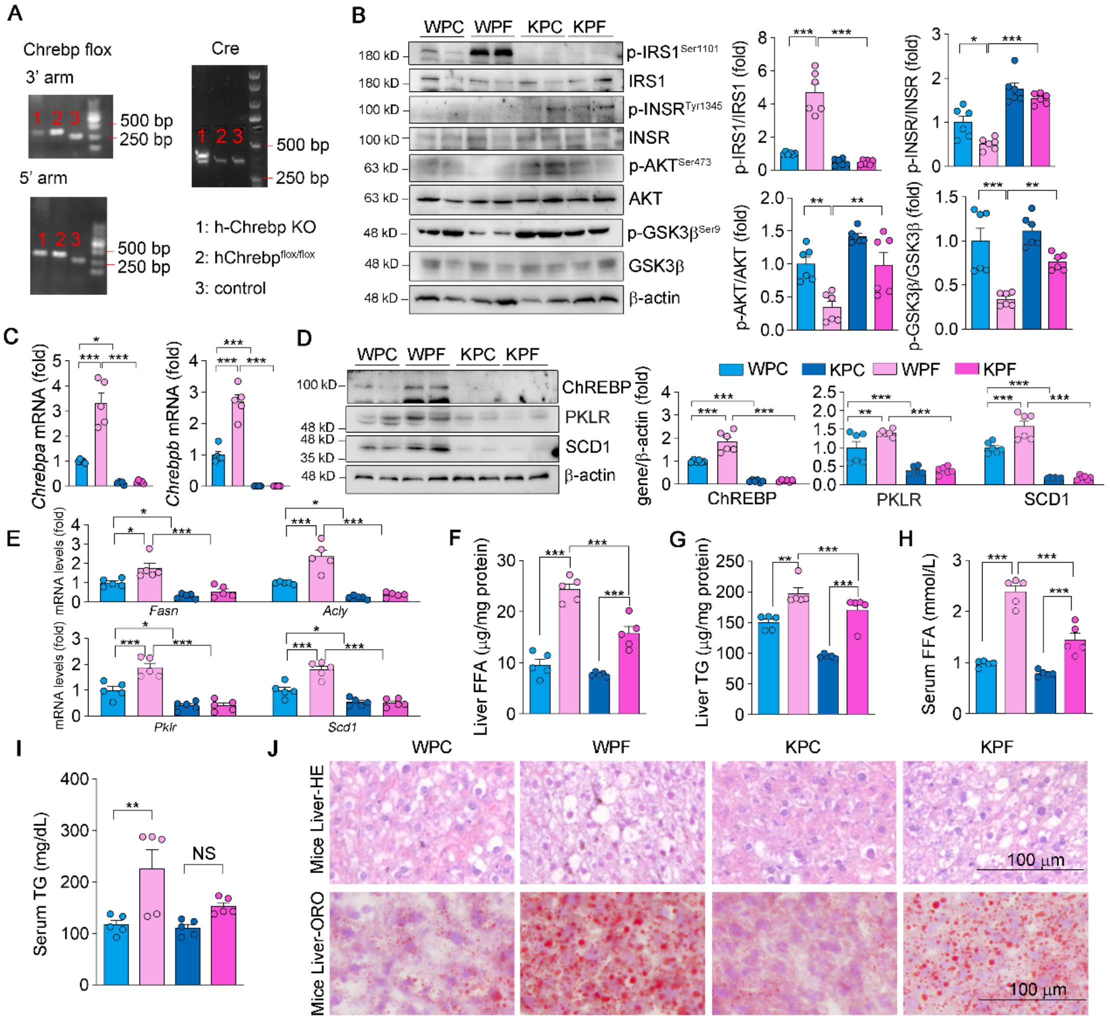

Appendix Figure S3. Hepatic ChREBP deficiency improved IR and hepatic lipid accumulation. The mice in **Figure 2A** were used to complete following assays: (**A**) Representative gel photographs illustrating the PCR products amplified from the mouse tail to identify the genotype in this study. (**B**) Expression of protein related to insulin signaling pathway in liver was determined by Western blot with quantification of the band density. n = 6. (**C**) Expression of *Chrebpa* and *Chrebpb* mRNA in liver was determined by qRT-PCR. n = 5. (**D**) Expression of ChREBP and its downstream targets protein in liver was determined by Western blot with quantification of the band density. n = 5. (**E**) mRNA expression of downstream targets gene of *Chrebp* in liver was determined by qRT-PCR. n = 5. (**F-I**) Hepatic (**F** and **G**) and serum (**H** and **I**) lipid extracts were subjected to free fatty acid (FFA) and triglyceride (TG) quantitative analysis. n = 5. (**J**) HE staining and Oil Red O (ORO) staining of liver sections. n = 5. Data information: All graphs are represented as Mean ± SEM, n: biological replicates. Two-way ANOVA followed by Tukey's multiple comparisons test were used. *P<0.05, **P<0.01, ***P<0.001, NS: no significance vs. indicated group.

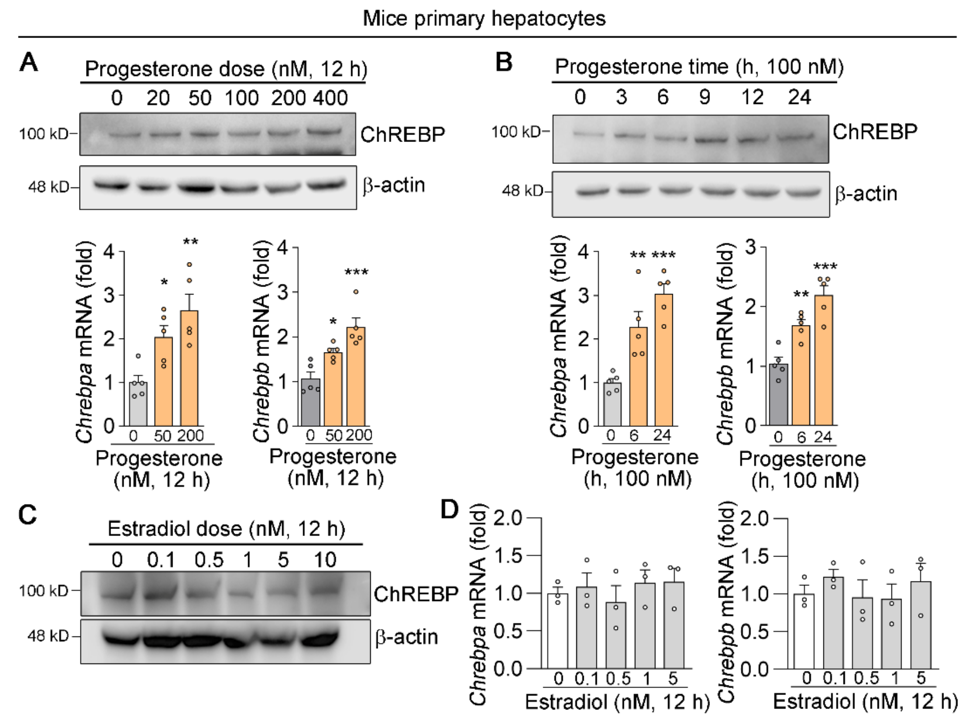

Appendix Figure S4. Progesterone but not estradiol activates hepatic ChREBP expression. (**A, B**) Primary hepatocyte was treated with progesterone at the indicated concentrations for 12 h (**A**) or 100 nM for the indicated time points (**B**), the ChREBP protein level was determined by Western blot, the *Chrebpa* and *Chrebpb* mRNA level was determined by qRT-PCR. n = 5. (**C, D**) Primary hepatocyte was treated with estradiol at the indicated concentrations for 12 h, the ChREBP protein level was determined by Western blot (**C**), the *Chrebpa* and *Chrebpb* mRNA level was determined by qRT-PCR (**D**), n = 3.

Data information: All graphs are represented as Mean ± SEM, n: biological replicates. One-way ANOVA followed by Tukey's multiple comparisons test were used. *P<0.05, **P<0.01, ***P<0.001 vs. control group.

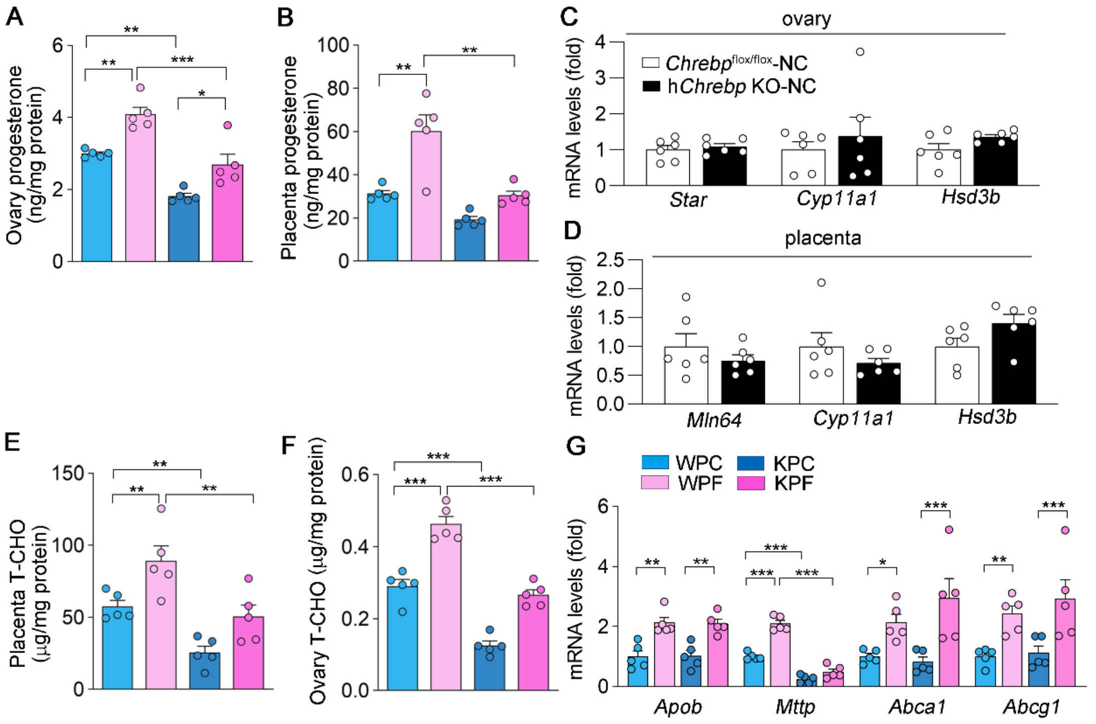

Appendix Figure S5. Hepatic ChREBP deficiency did not affect the expression of progesterone synthases. (**A**) Determination of progesterone levels in the ovary of mice, n = 5. (**B**) Determination of progesterone levels in the placenta of mice, n = 5. (**C, D**) mRNA levels of progesterone synthesis-related genes in ovary and placenta were determined by qRT-PCR analysis, n = 6. (**E, F**) Determination of total cholesterol (T-CHO) levels in placenta (**E**), ovary (**F**) of mice, n = 5. (**G**) Expression of genes involved in cholesterol transport was determined by qRT–PCR in mouse liver, n = 5.

Data information: All graphs are represented as Mean ± SEM, n: biological replicates. Two-way ANOVA followed by Tukey's multiple comparisons test were used. *P<0.05, **P<0.01, ***P<0.001 vs. the indicated group.

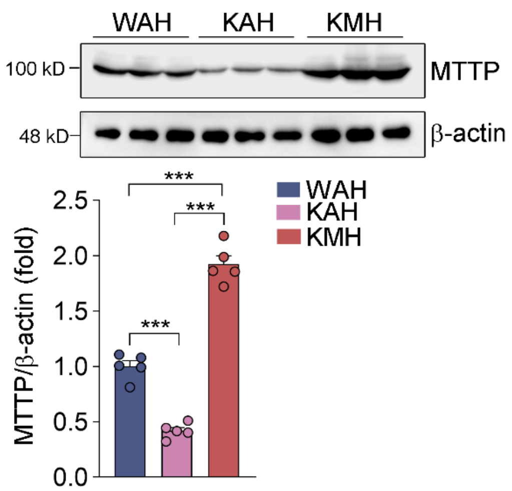

Appendix Figure S6. AAV-mediated overexpression of MTTP in the liver. Expression of MTTP protein in **Figure 4F** mouse liver was determined by Western blot with quantification of band density. n = 5.

Data information: All graphs are represented as Mean ± SEM, n: biological replicates. Two-way ANOVA followed by Tukey's multiple comparisons test were used. ***P<0.001 vs. indicated group.

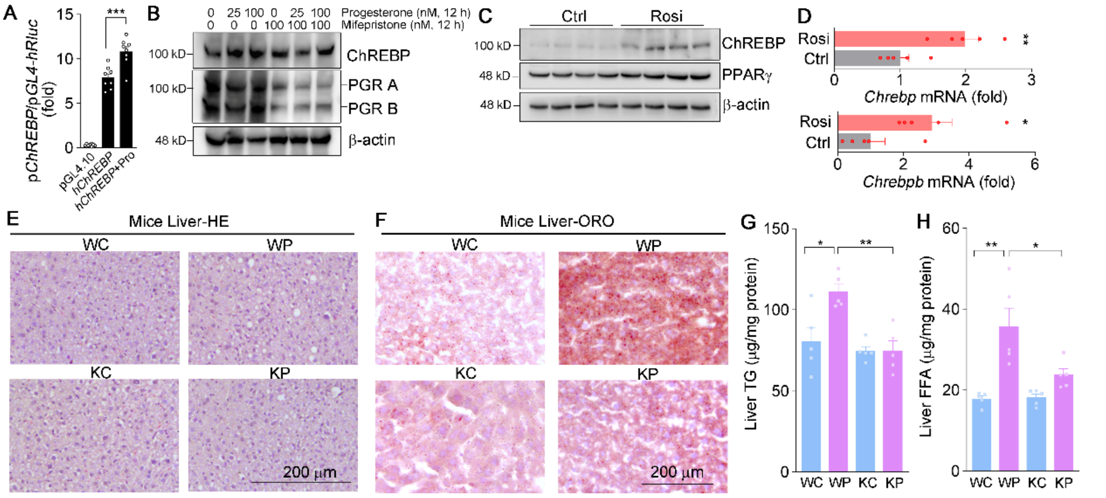

Appendix Figure S7. Progesterone activates hepatic ChREBP expression by PPARγ. (A) HepG2 cells were transfected with the h*ChREBP* promoter plus Renilla (as an internal control) overnight, followed by 100 nM progesterone treatment for another 12 h. The activity of firefly and renilla luciferases in the cellular lysate was determined by dual-luciferase reporter assay (n = 8). (**B**) Primary hepatocytes were treated with progesterone at indicated concentrations with 0 or 100 nM mifepristone for 12 h and protein level was determined by Western blot. (**C, D**) Female C57BL/6J mice were gavaged with vehicle (saline solution, Ctrl groups) or rosiglitazone solution (30 mg/kg body weight, Rosi groups) in saline solution for 5 consecutive days. Mouse livers were collected for determination of proteins and mRNA expression by Western blot (**C**) and qRT-PCR (**D**), n = 5. (**E-H**) Mouse liver sections of **Figure 5I** were conducted HE staining (**E**) and Oil Red O (ORO) staining (**F**); hepatic lipid extracts were subjected to triglyceride (TG) (**G**) and free fatty acid (FFA) (**H**) quantitative analysis, n = 5.

Data information: All graphs are represented as Mean ± SEM, n: biological replicates. In (A), One-way ANOVA followed by Tukey's multiple comparisons test were used. ***$P<0.001$ vs. the indicated group. In (D), Two-tailed t-tests were performed to assess differences between two experimental groups. *$P<0.05$, **$P<0.01$ vs. control group. In (G, H), Two-way ANOVA followed by Tukey's multiple comparisons test were used. *$P<0.05$, **$P<0.01$ vs. the indicated group.

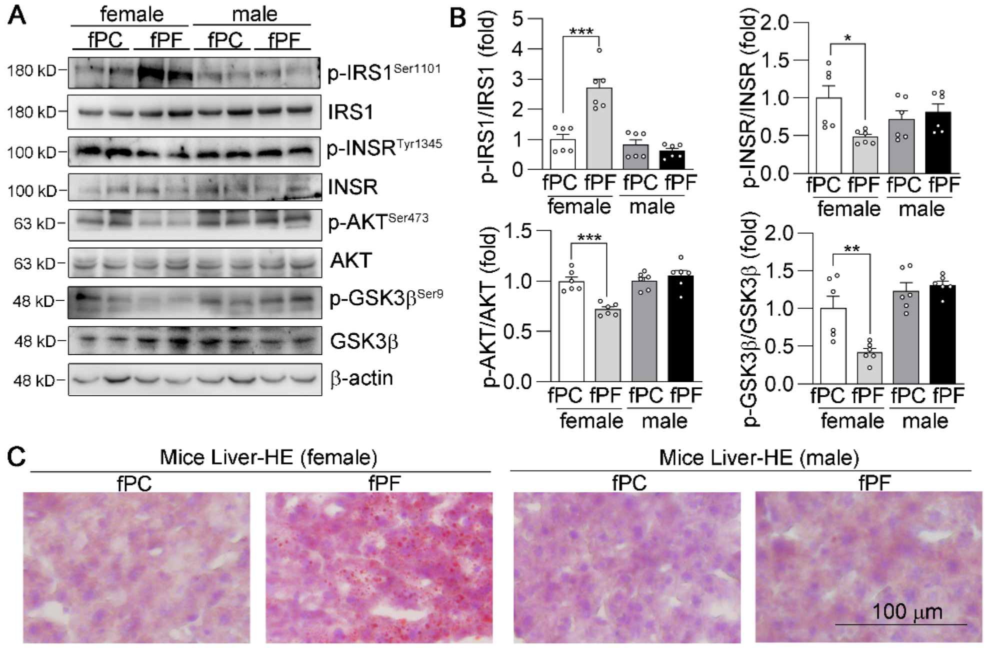

Appendix Figure S8. HFrD aggravates glucose and lipid metabolism disorders in female offspring. The mice in **Figure 6A** were used to complete following assays: (**A-C**) Expression of insulin signaling pathway-related proteins in mouse liver was determined by Western blot (**A**) with quantification of band density (**B**). Oil Red O (ORO) staining of liver sections (**C**). n = 6, *P<0.05, **P<0.01, ***P<0.001 vs. indicated group.

Data information: All graphs are represented as Mean ± SEM, n: biological replicates. One-way ANOVA followed by Tukey's multiple comparisons test were used. *P<0.05, **P<0.01, ***P<0.001 vs. indicated group.

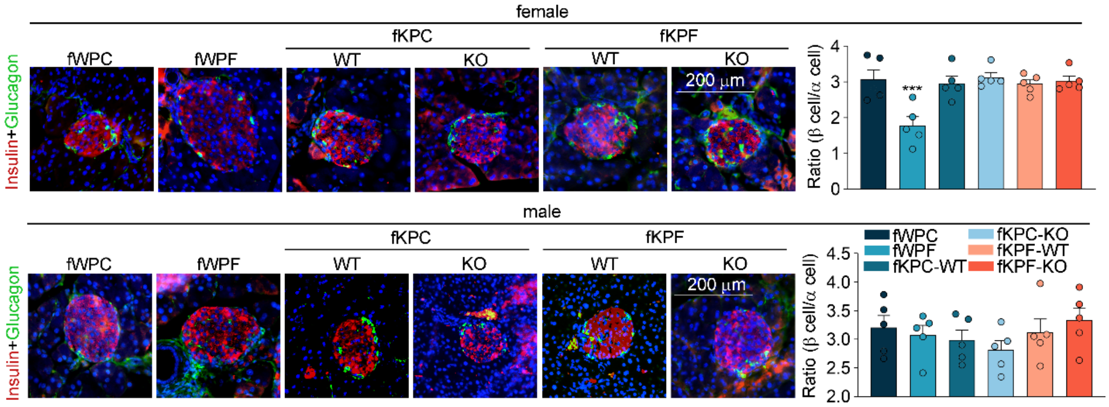

Appendix Figure S9. Maternal hepatic ChREBP deficiency improves HFrD-impaired pancreas in female offspring. Immunofluorescent staining with anti-glucagon (green) or anti-insulin (red) antibodies of mice pancreas sections, and the ratio of beta cell area (insulin-positive) to alpha cell area (glucagon positive) was calculated. n = 5.

Data information: All graphs are represented as Mean ± SEM, n: biological replicates. One-way ANOVA followed by Tukey's multiple comparisons test were used. ***P<0.001 vs. fWPC group.

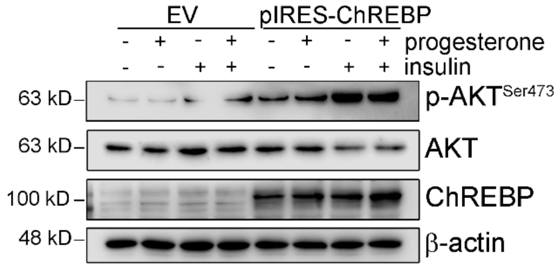

Appendix Figure S10. Hepatic ChREBP improves hepatic insulin sensitivity in vitro. HepG2 cells were transfected with pIRES-*ChREBP* or pIRES-*HA* (empty vectors, EV) overnight and then treated with or without 100 nM progesterone (PRO) overnight as well as 10 nM insulin for 15 min. Expression of protein related to insulin signaling pathway was determined by Western blot.

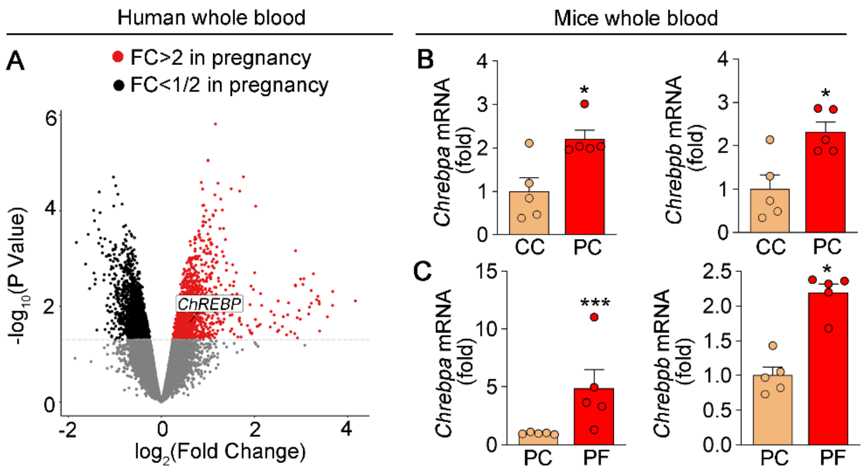

Appendix Figure S11. ChREBP mRNA level in blood was activated by pregnancy and HFrD-diet. (**A**) Volcano plot showing the transcripts in healthy donors' peripheral blood mononuclear cells (PBMCs) at the 9th month of pregnancy that were decreased (black dots), increased (red dots), or not changed (gray dots). The data were generated from the NCBI gene expression omnibus database (GEO access: GSE17409). $p<0.05$; n = 3 (healthy donors at the 9th month of pregnancy), n = 5 (nonpregnant subjects). (**B**) Whole blood was obtained from unpregnancy mice (CC group) and pregnancy mice (PC group). RNA was extracted by the blood total RNA kit (Zomanbio, China), and *Chrebpa* and *Chrebpb* mRNA levels were evaluated by qRT-PCR. n = 5. (**C**) Whole blood was obtained from normal diet pregnancy mice (PC group) and HFrD diet pregnancy mice (PF group). RNA was extracted by the blood total RNA kit (Zomanbio, China), and *Chrebpa* and *Chrebpb* mRNA levels were evaluated by qRT-PCR. n = 5.

Data information: All graphs are represented as Mean ± SEM, n: biological replicates. Two-tailed t-tests were performed to assess differences between two experimental groups. In (B), *P<0.05 vs. CC group. In (C), *P<0.05, ***P<0.001 vs. PC group.
