# Supplementary material for: Liver ChREBP deficiency inhibits fructose-induced insulin resistance in pregnant mice and female offspring
Source: EMBO Rep. 2024 Mar 26;25(4):25. doi: 10.1038/s44319-024-00121-w (PMC11014959; doi:10.1038/s44319-024-00121-w)
Supplement: Supplementary file 2 — Source data Fig. 1 [file 44319_2024_121_MOESM2_ESM.zip › Figure 1/H/Results of statistical analysis of band density for Western blot.docx]

**Results of statistical analysis of band density for Western blot**

All the Western blot images were conducted analysis of band density, and normalized to the density of β-actin in the corresponding samples.

**Figure 1**

**Figure 1H:** (*P<0.05, **P<0.01, ***P<0.001 *vs.* CC, ^#^P<0.05, ^##^P<0.01, ^###^P<0.001 *vs.* CF, ^^^P<0.05, ^^^^P<0.01, ^^^^^P<0.001 *vs.* PC, n = 3)

| **Genes** | **CC** | **CF** | **PC** | **PF** |
| --- | --- | --- | --- | --- |
| ChREBP | 100±3 | 129±3* | 140±8** | 172±11^##^^ |
| PKLR | 100±6 | 165±13*** | 154±20*** | 228±31^###^^^^ |
| SCD1 | 100±30 | 280±33* | 291±71* | 460±44^#^^ |
