# Supplementary figures and images for: Liver ChREBP deficiency inhibits fructose-induced insulin resistance in pregnant mice and female offspring
Source: EMBO Rep. 2024 Mar 26;25(4):25. doi: 10.1038/s44319-024-00121-w (PMC11014959; doi:10.1038/s44319-024-00121-w)

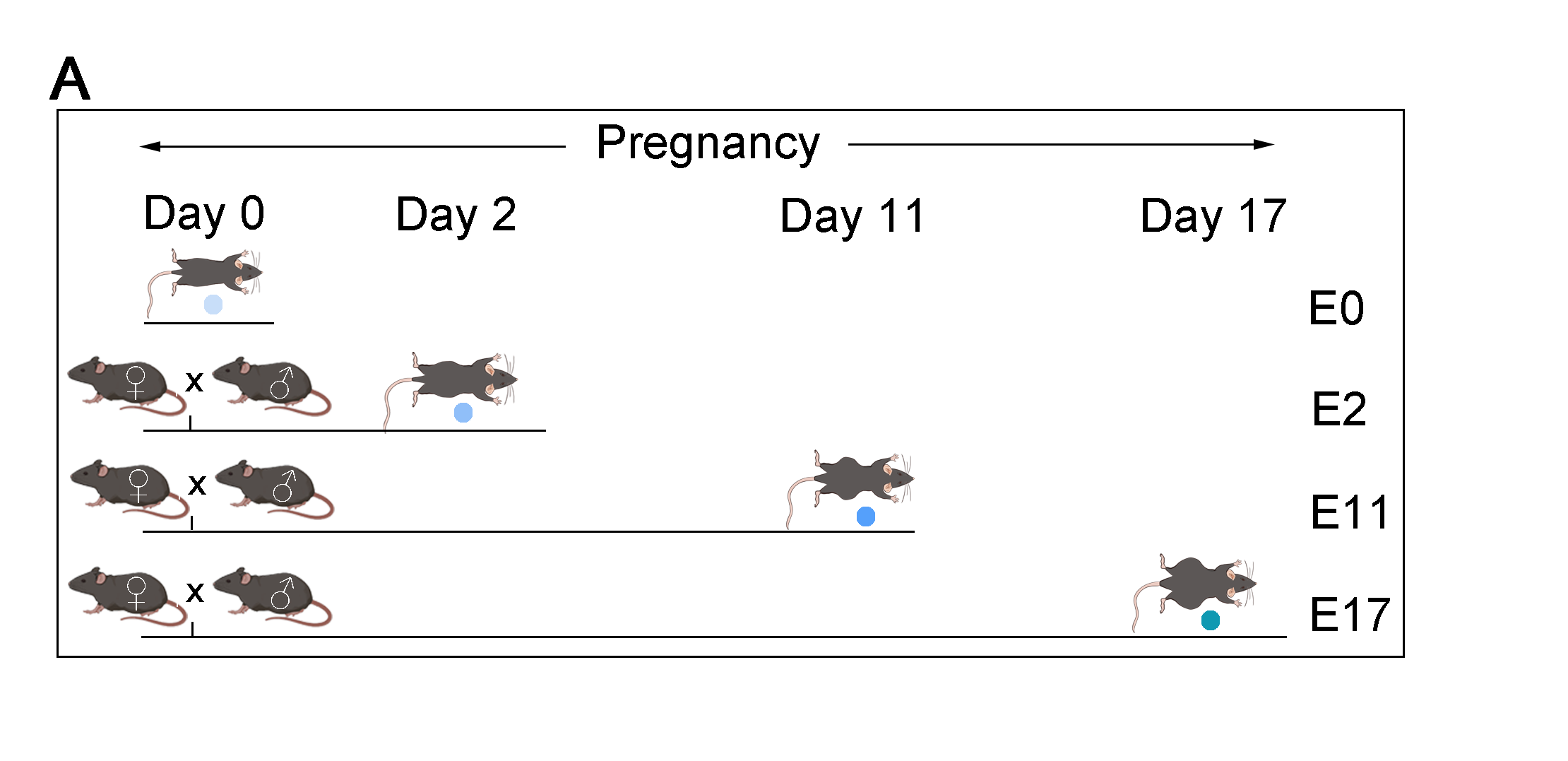

Supplement: Supplementary file 2 — Source data Fig. 1 [file 44319_2024_121_MOESM2_ESM.zip › Figure 1/A/Figure 1A.tif]

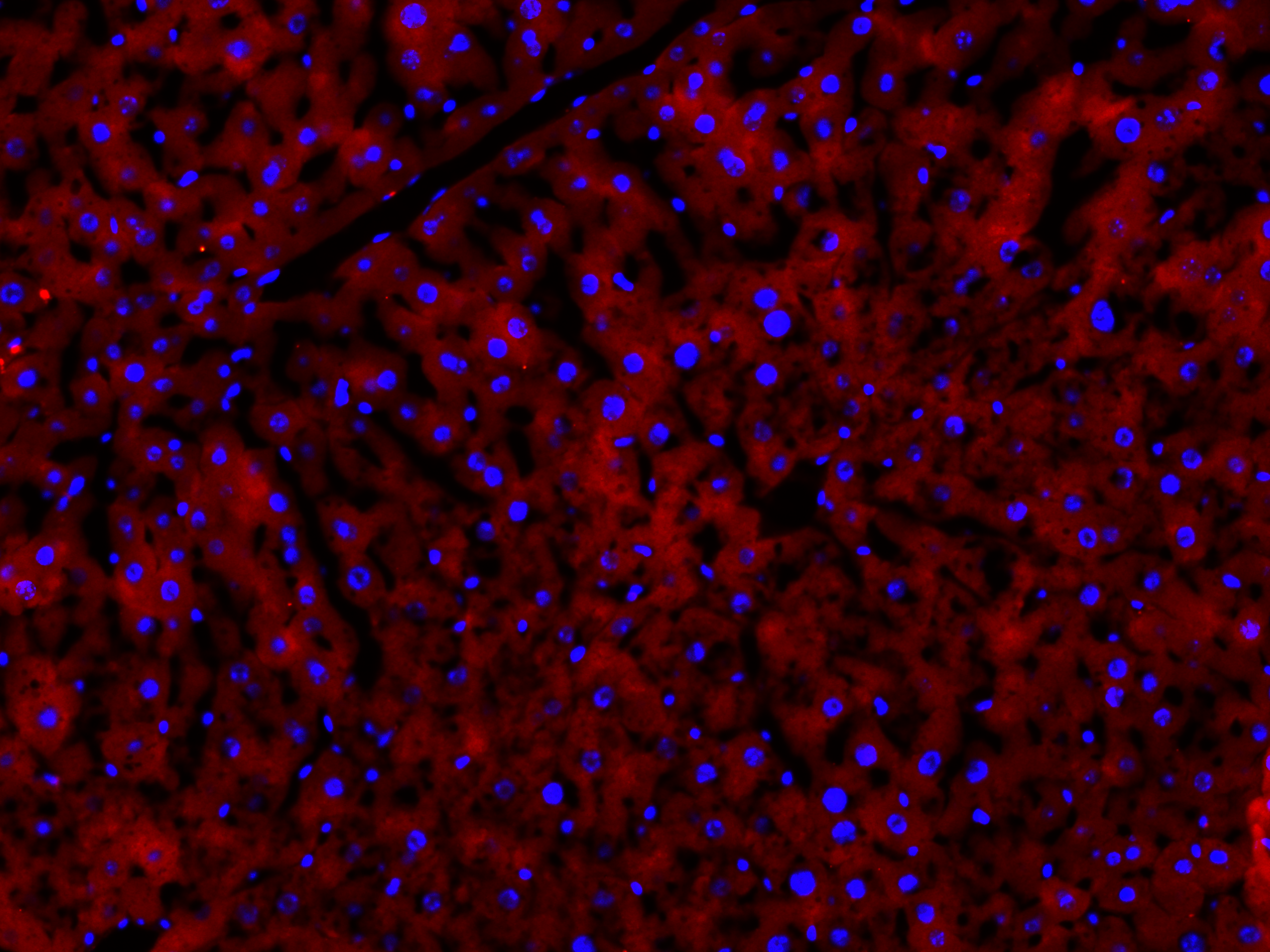

Supplement: Supplementary file 2 — Source data Fig. 1 [file 44319_2024_121_MOESM2_ESM.zip › Figure 1/C/1-E0.tif]

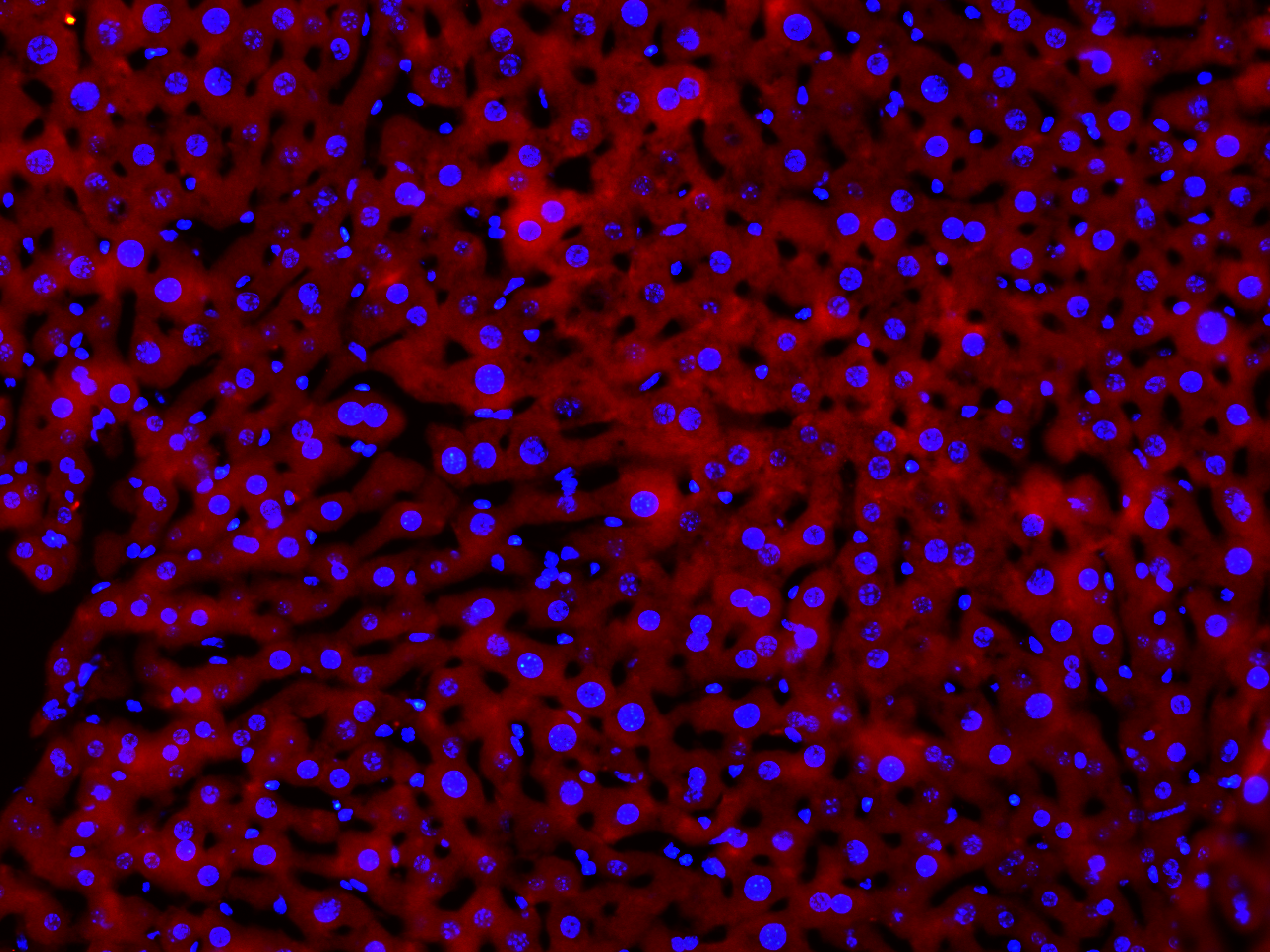

Supplement: Supplementary file 2 — Source data Fig. 1 [file 44319_2024_121_MOESM2_ESM.zip › Figure 1/C/2-E2.tif]

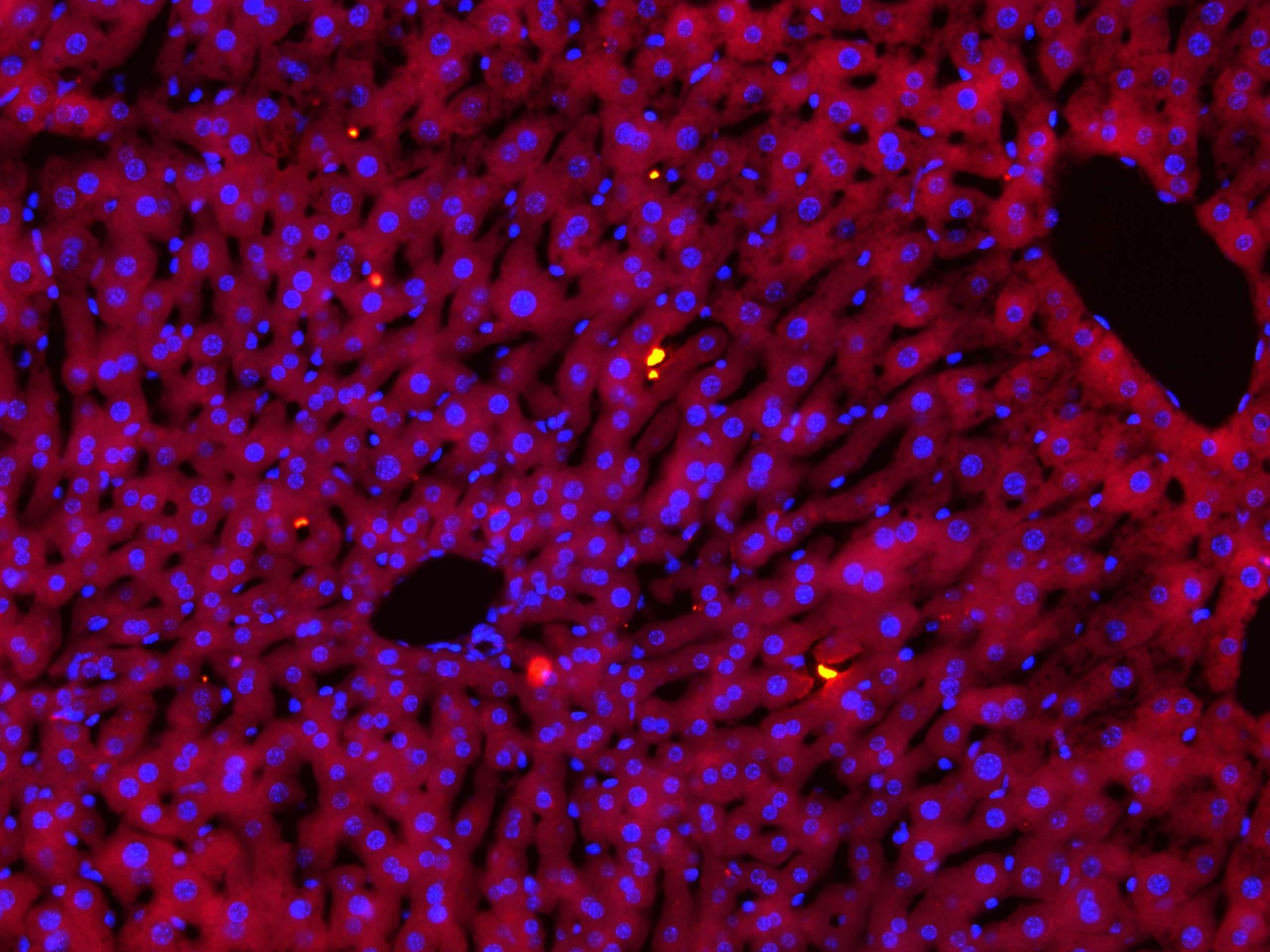

Supplement: Supplementary file 2 — Source data Fig. 1 [file 44319_2024_121_MOESM2_ESM.zip › Figure 1/C/3-E11.tif]

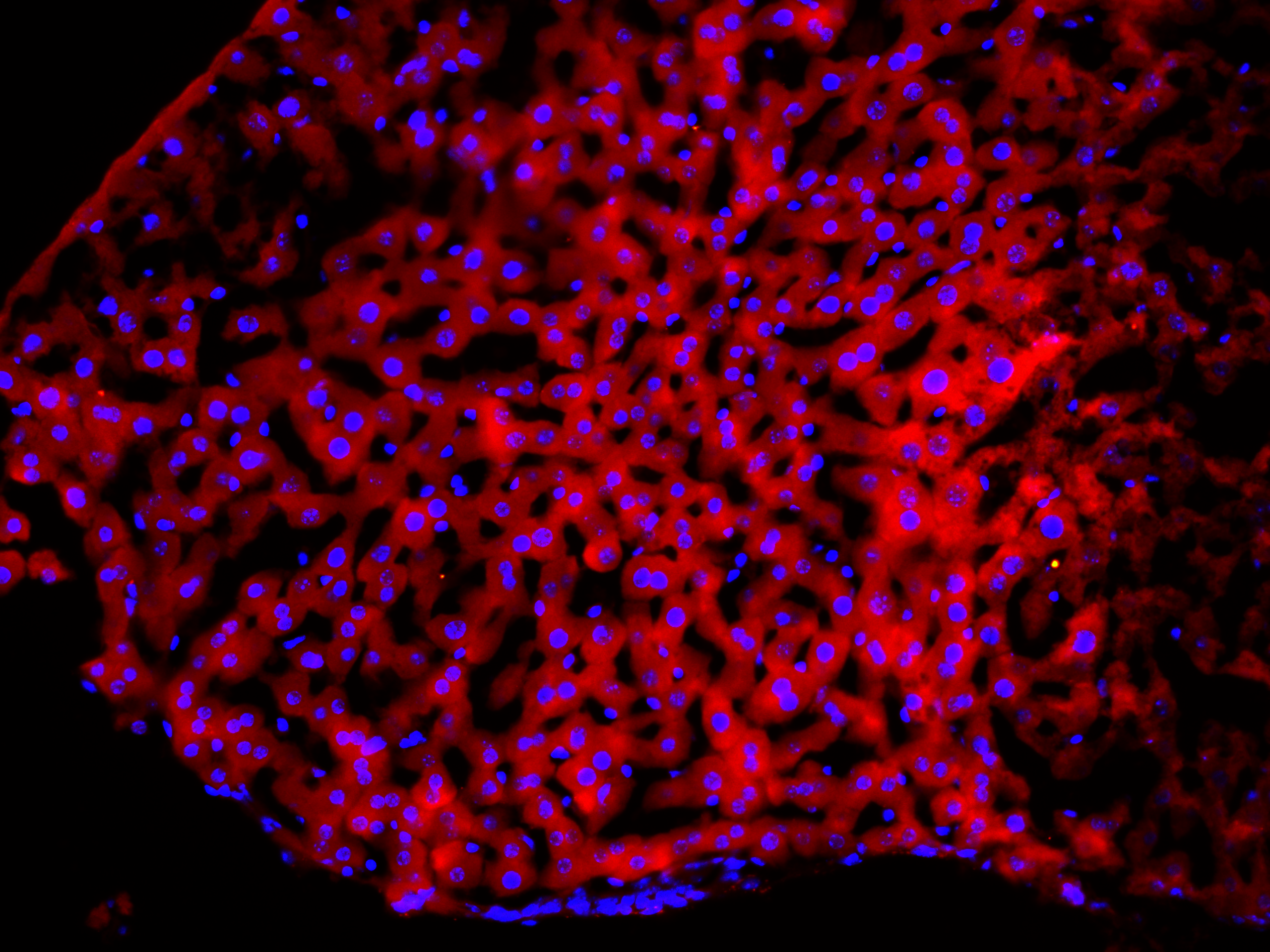

Supplement: Supplementary file 2 — Source data Fig. 1 [file 44319_2024_121_MOESM2_ESM.zip › Figure 1/C/4-E17.tif]

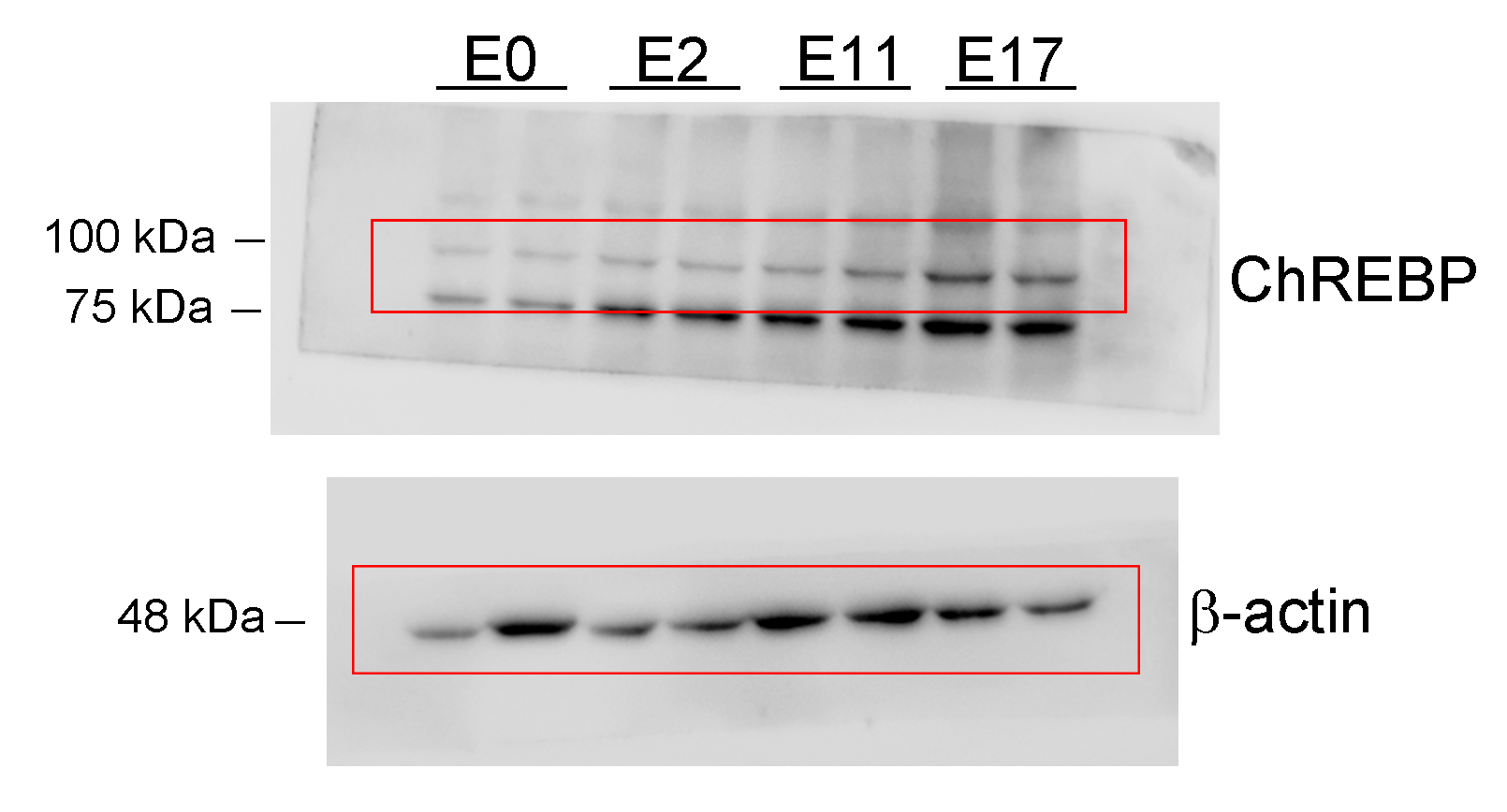

Supplement: Supplementary file 2 — Source data Fig. 1 [file 44319_2024_121_MOESM2_ESM.zip › Figure 1/D/Figure 1D.tif]

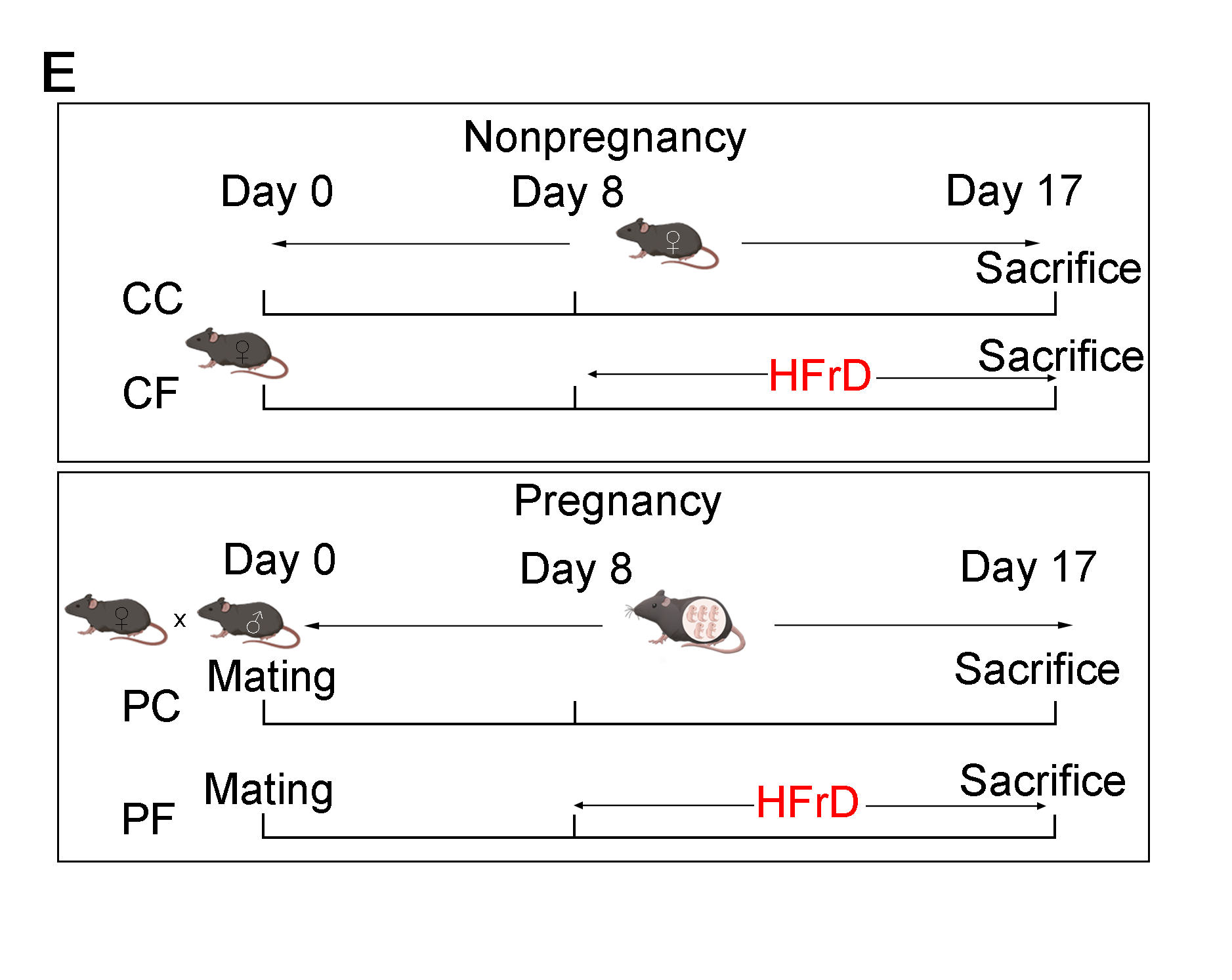

Supplement: Supplementary file 2 — Source data Fig. 1 [file 44319_2024_121_MOESM2_ESM.zip › Figure 1/E/Figure 1E.tif]

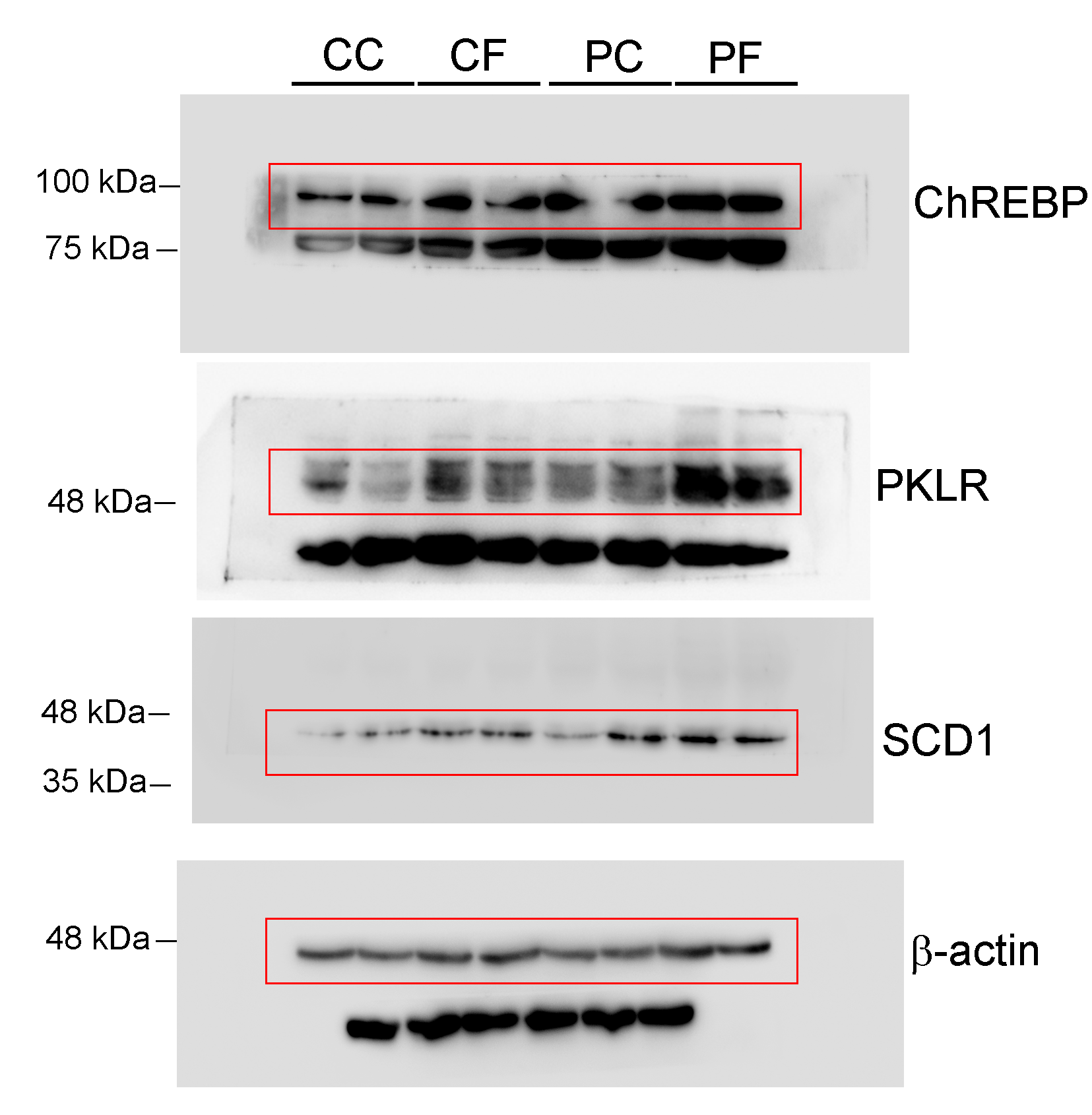

Supplement: Supplementary file 2 — Source data Fig. 1 [file 44319_2024_121_MOESM2_ESM.zip › Figure 1/H/Figure 1H.tif]

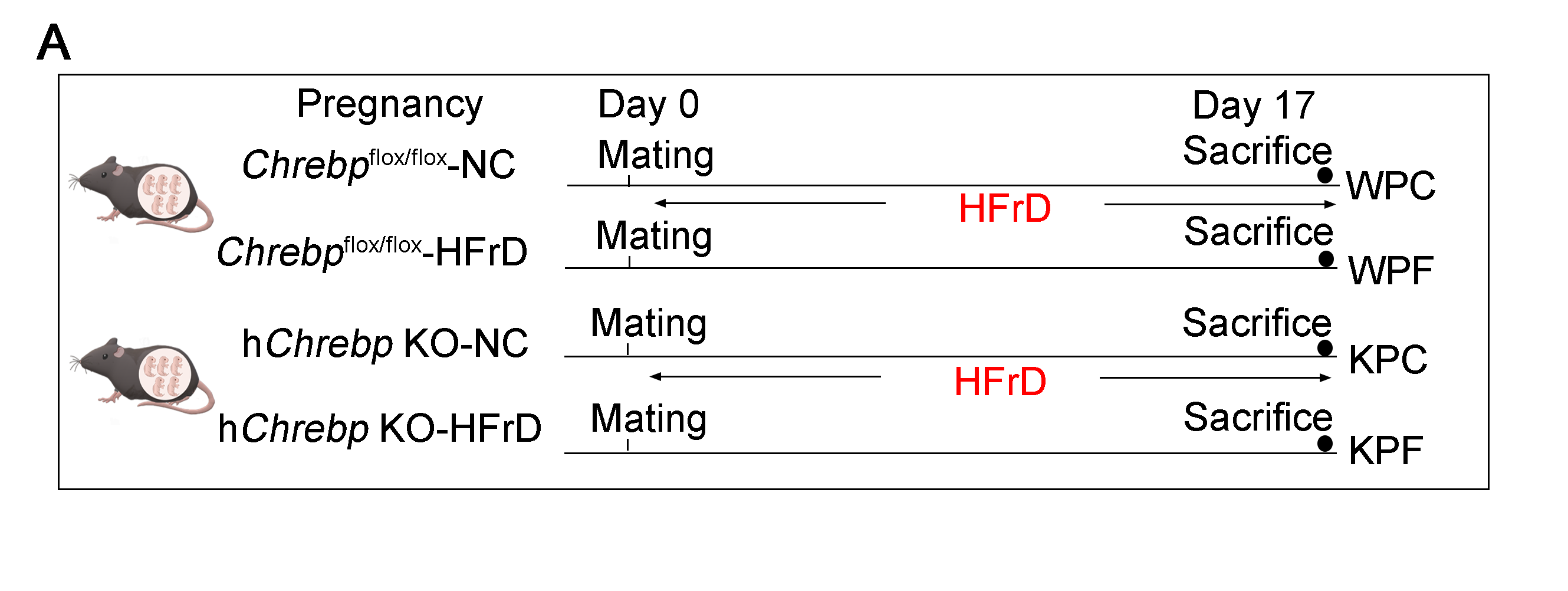

Supplement: Supplementary file 3 — Source data Fig. 2 [file 44319_2024_121_MOESM3_ESM.zip › Figure 2/A/Figure 2A.tif]

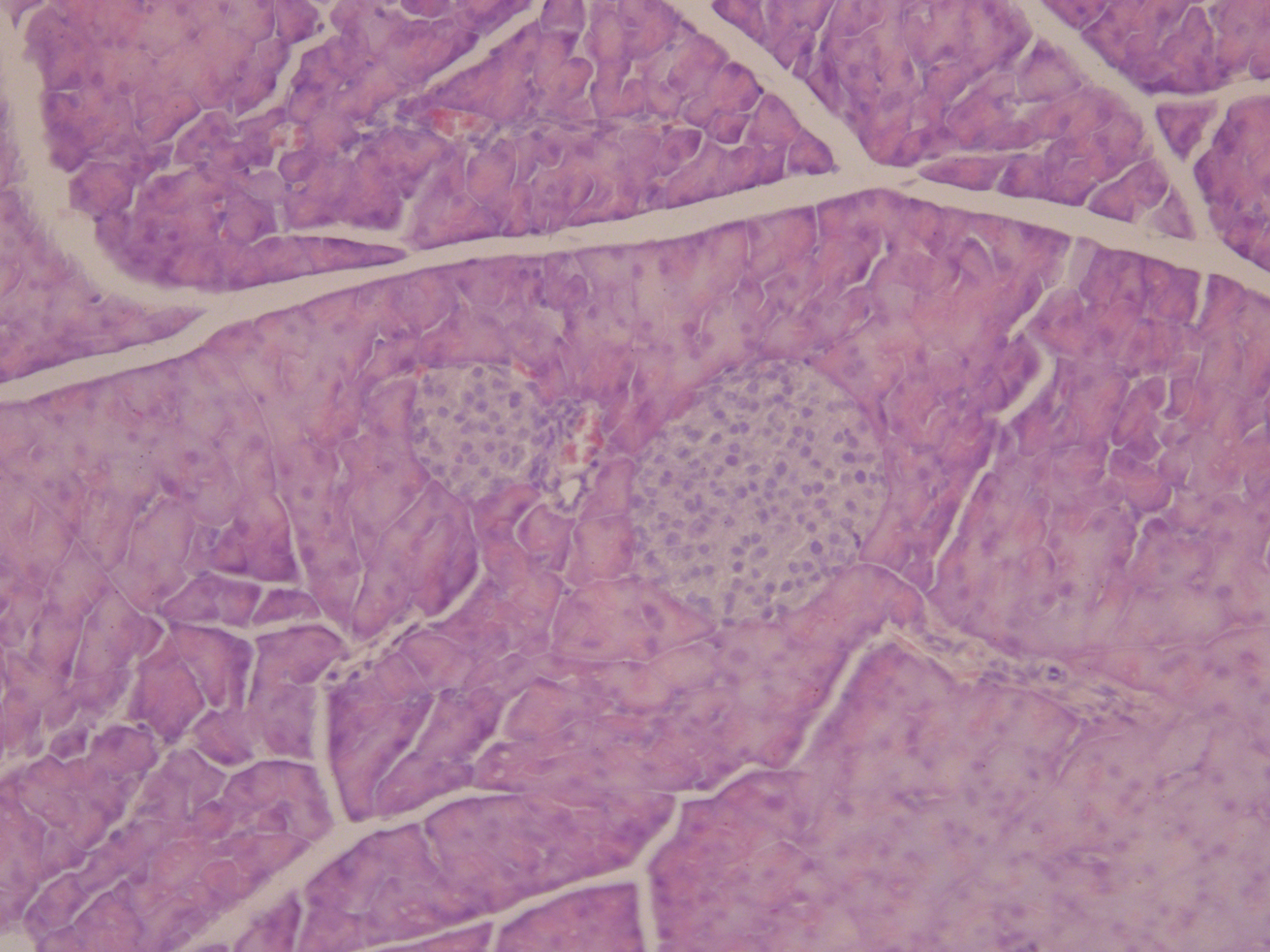

Supplement: Supplementary file 3 — Source data Fig. 2 [file 44319_2024_121_MOESM3_ESM.zip › Figure 2/G/1-WPC.tif]

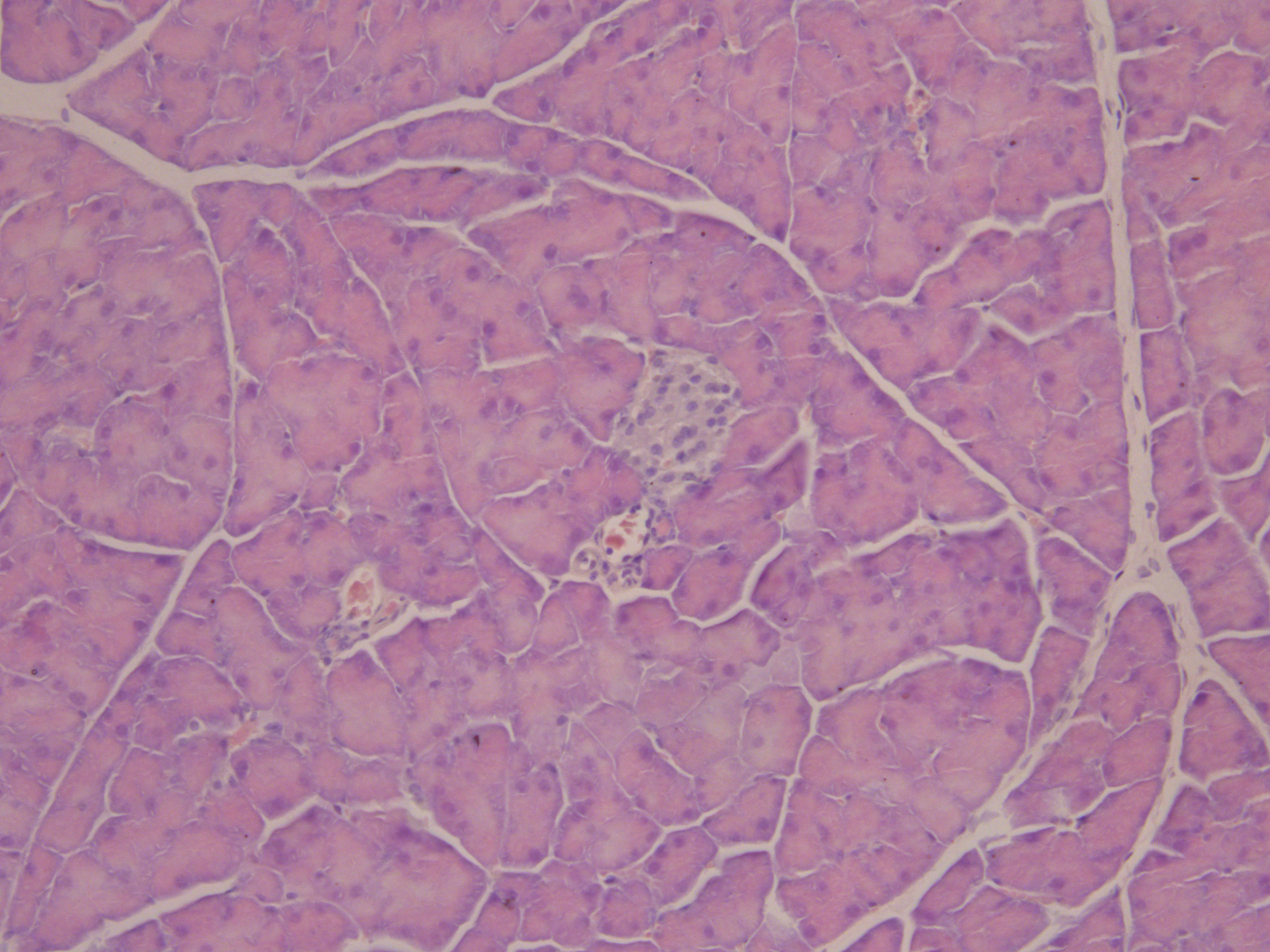

Supplement: Supplementary file 3 — Source data Fig. 2 [file 44319_2024_121_MOESM3_ESM.zip › Figure 2/G/2-WPF.tif]

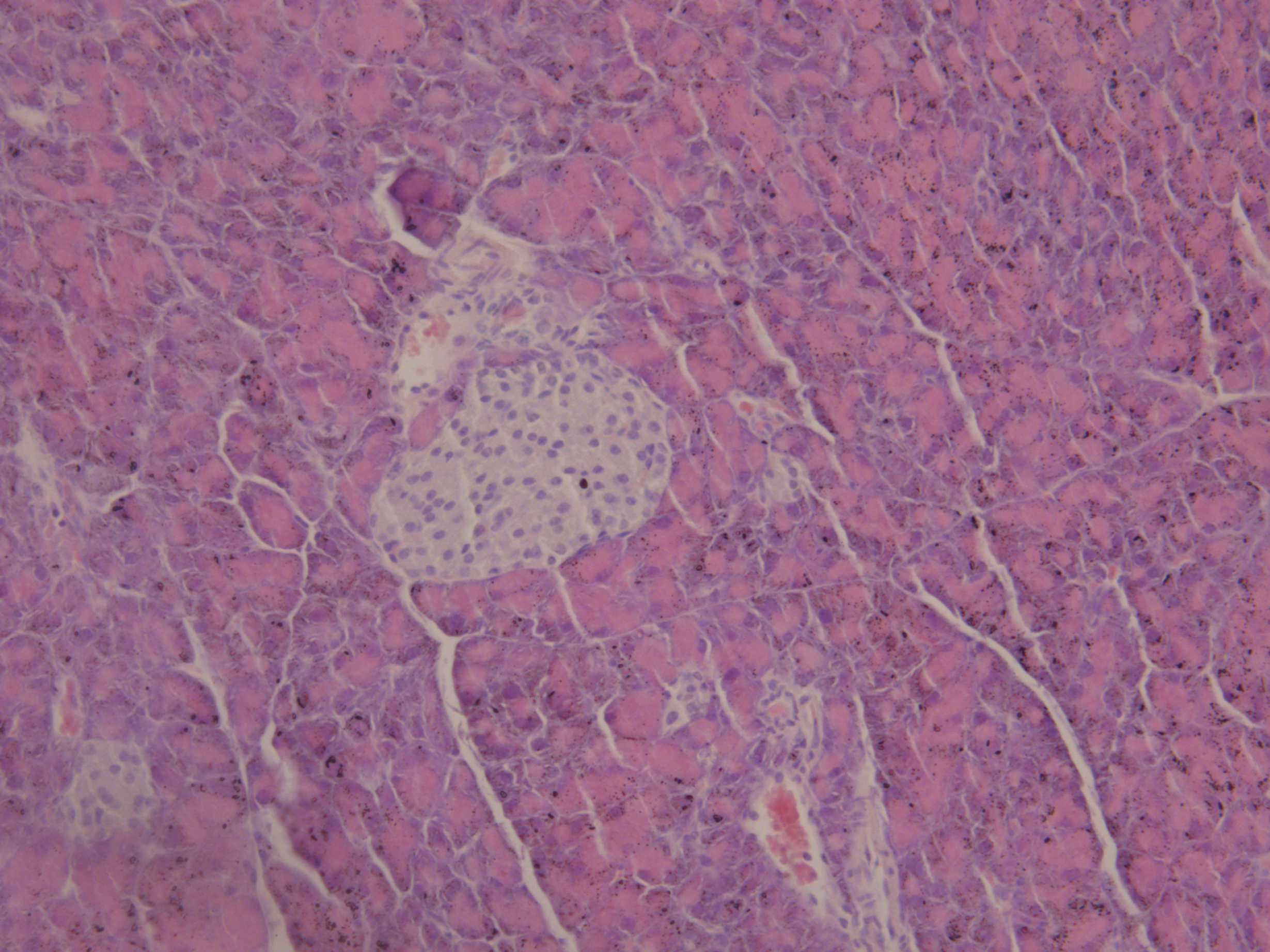

Supplement: Supplementary file 3 — Source data Fig. 2 [file 44319_2024_121_MOESM3_ESM.zip › Figure 2/G/3-KPC.tif]

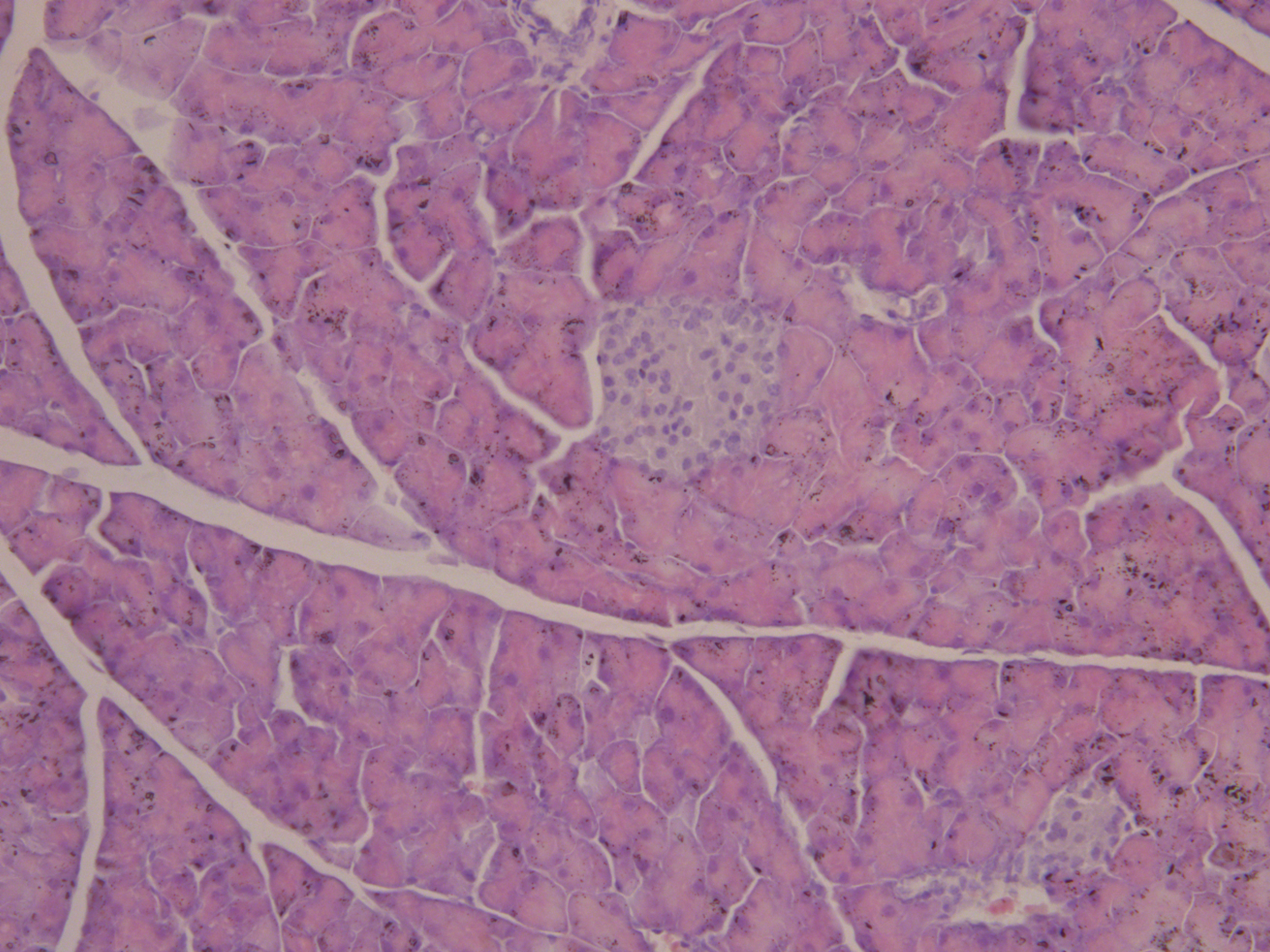

Supplement: Supplementary file 3 — Source data Fig. 2 [file 44319_2024_121_MOESM3_ESM.zip › Figure 2/G/4-KPF.tif]

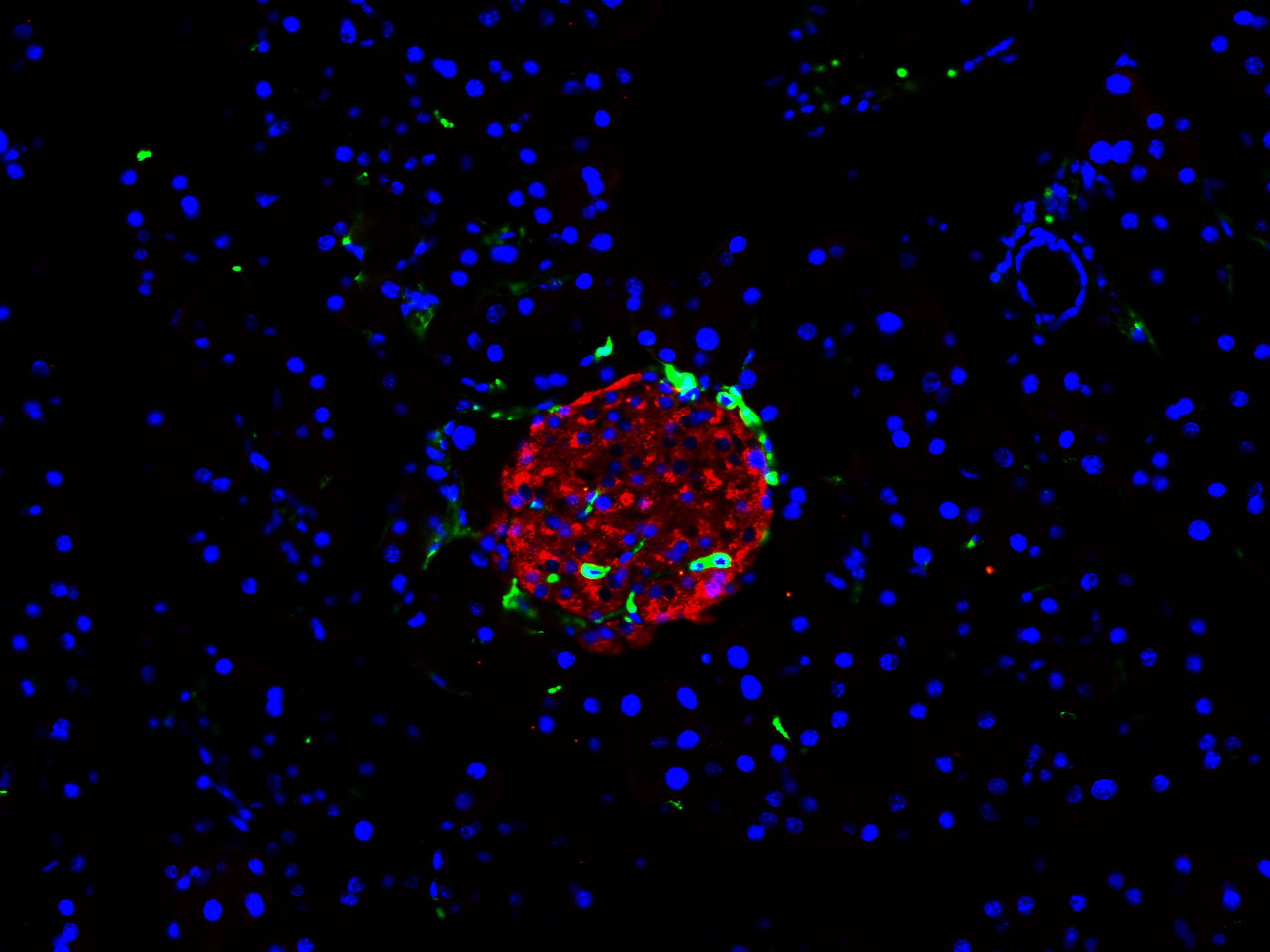

Supplement: Supplementary file 3 — Source data Fig. 2 [file 44319_2024_121_MOESM3_ESM.zip › Figure 2/H/1-WPC.tif]

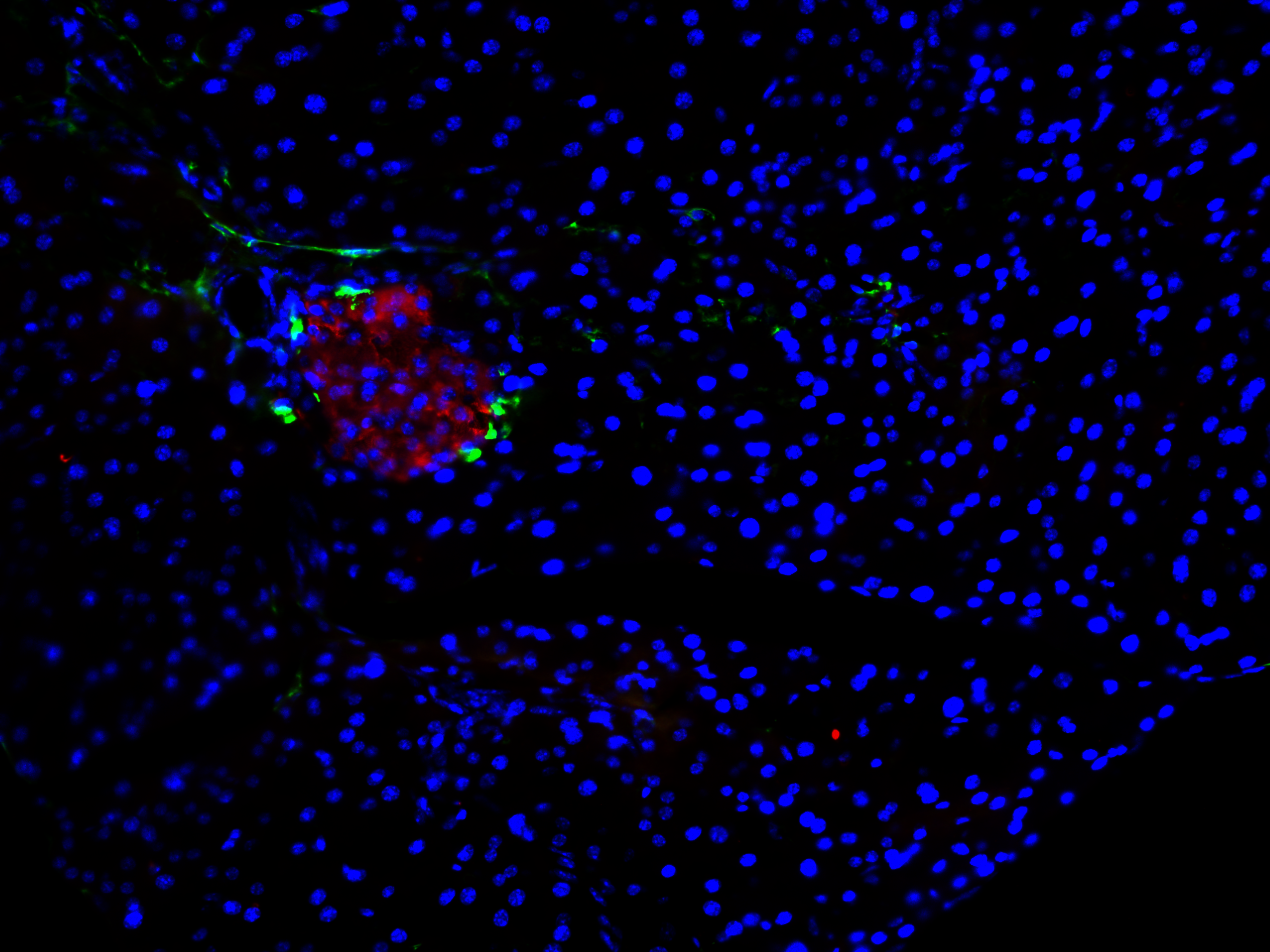

Supplement: Supplementary file 3 — Source data Fig. 2 [file 44319_2024_121_MOESM3_ESM.zip › Figure 2/H/2-WPF.tif]

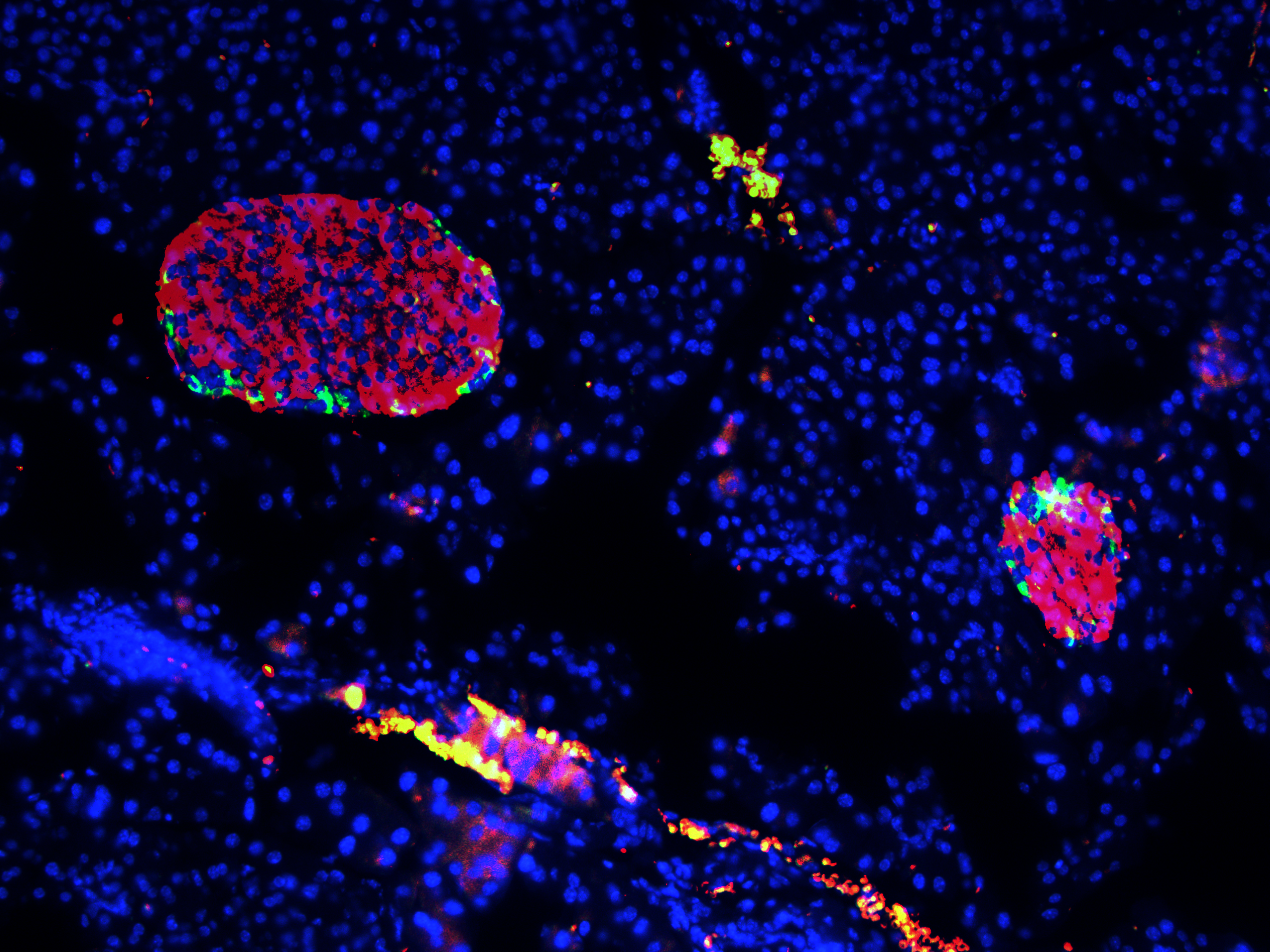

Supplement: Supplementary file 3 — Source data Fig. 2 [file 44319_2024_121_MOESM3_ESM.zip › Figure 2/H/3-KPC.tif]

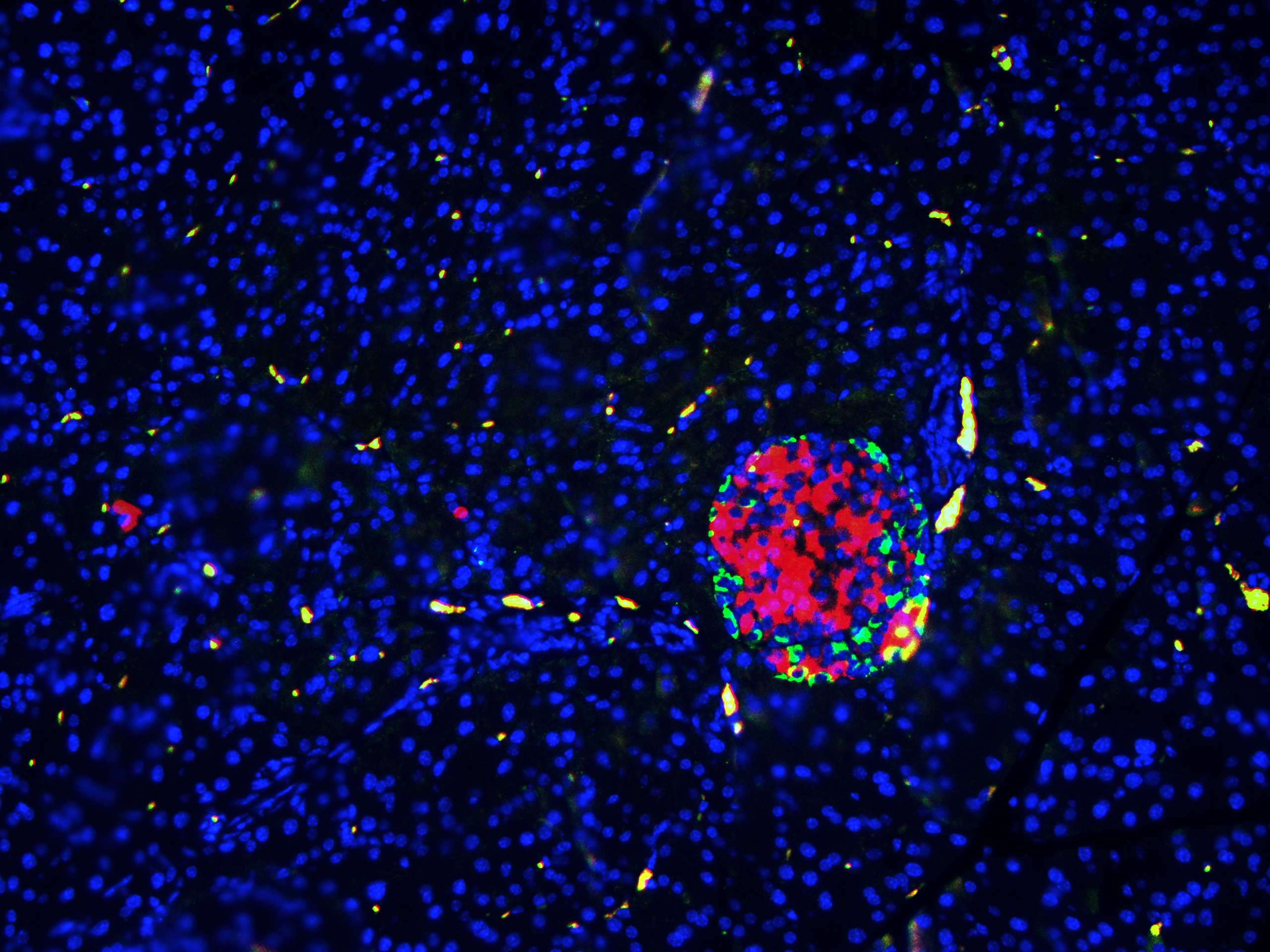

Supplement: Supplementary file 3 — Source data Fig. 2 [file 44319_2024_121_MOESM3_ESM.zip › Figure 2/H/4-KPF.tif]

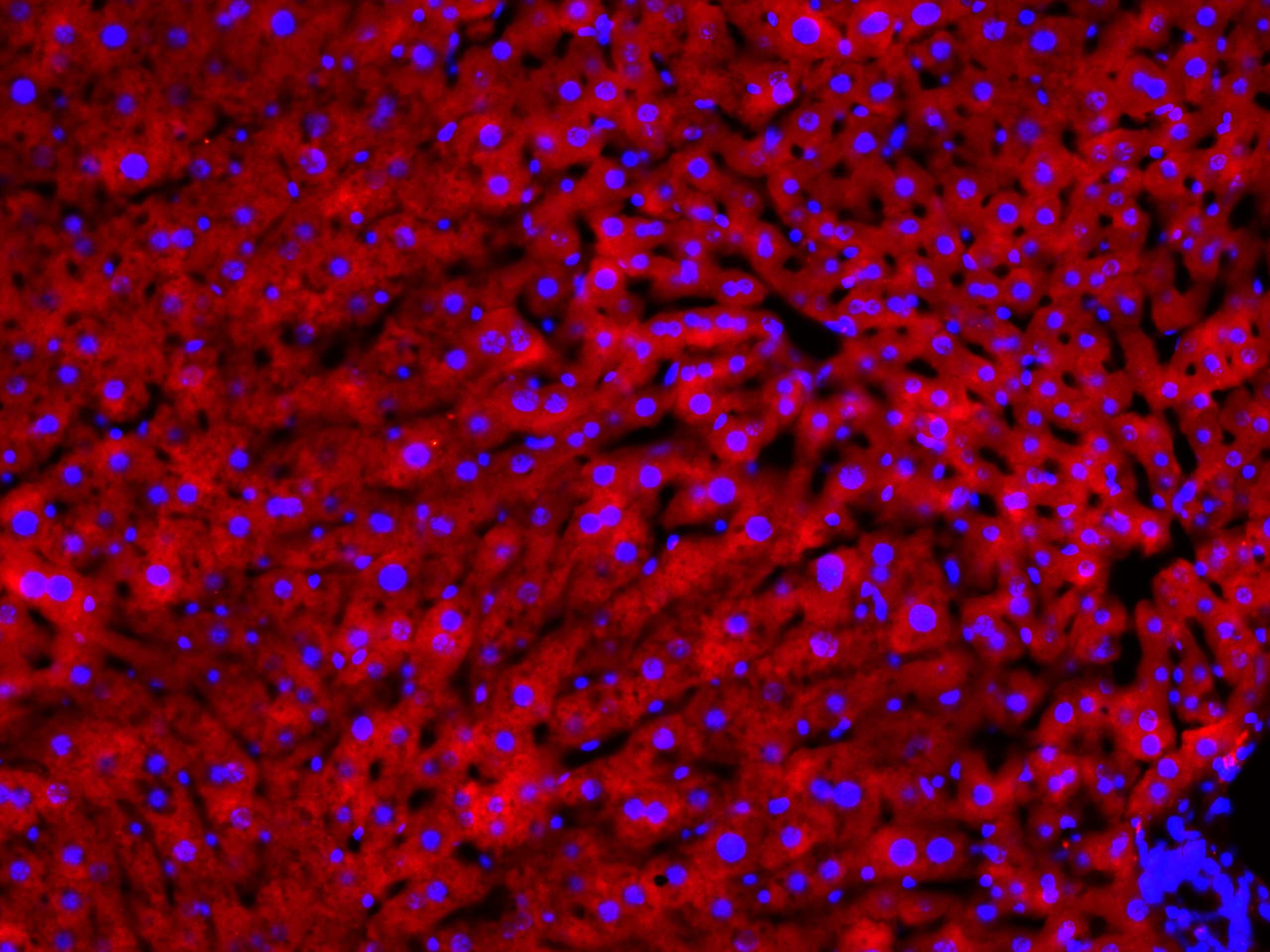

Supplement: Supplementary file 3 — Source data Fig. 2 [file 44319_2024_121_MOESM3_ESM.zip › Figure 2/I/1-WPC.tif]

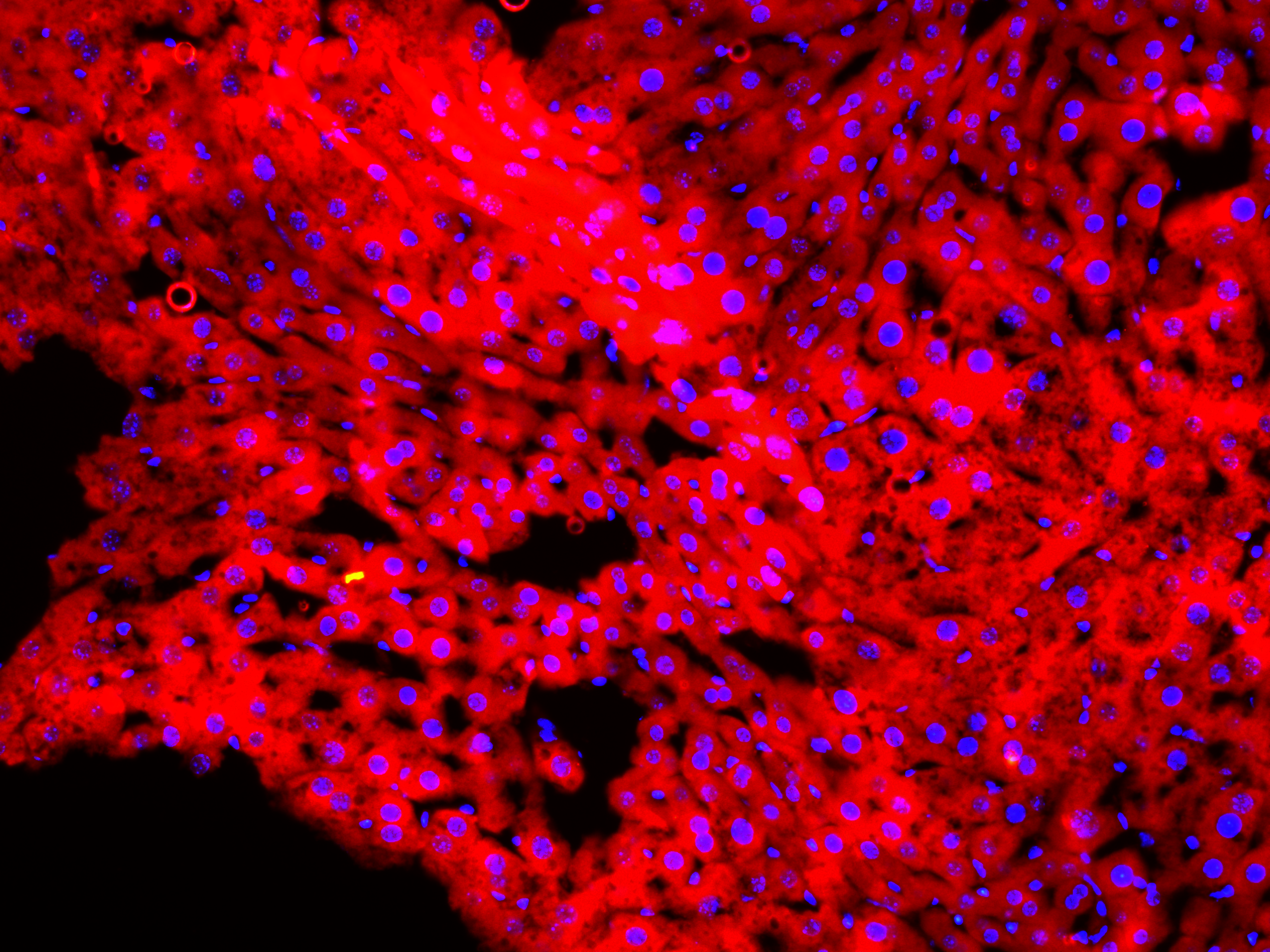

Supplement: Supplementary file 3 — Source data Fig. 2 [file 44319_2024_121_MOESM3_ESM.zip › Figure 2/I/2-WPF.tif]

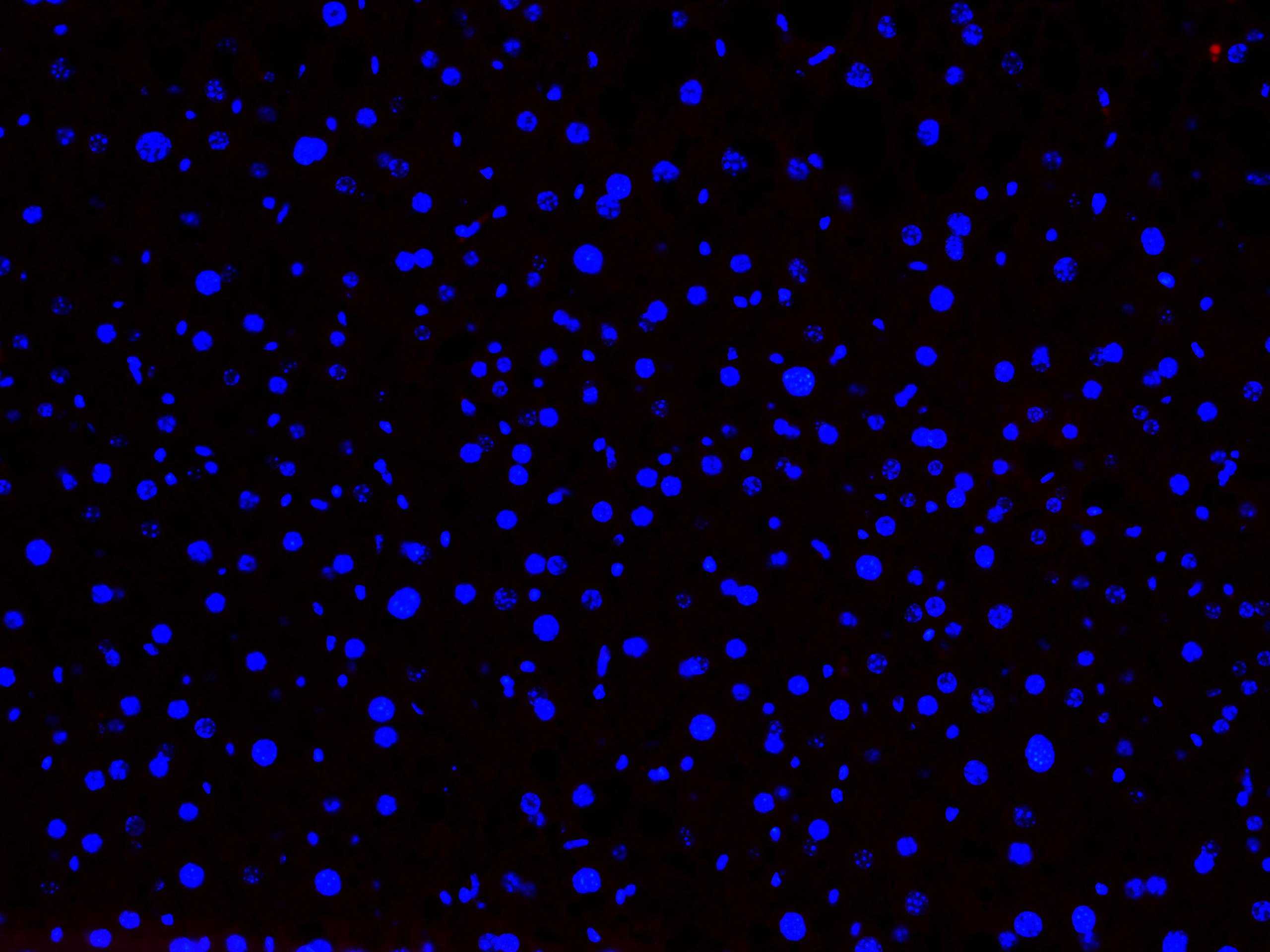

Supplement: Supplementary file 3 — Source data Fig. 2 [file 44319_2024_121_MOESM3_ESM.zip › Figure 2/I/3-KPC.tif]

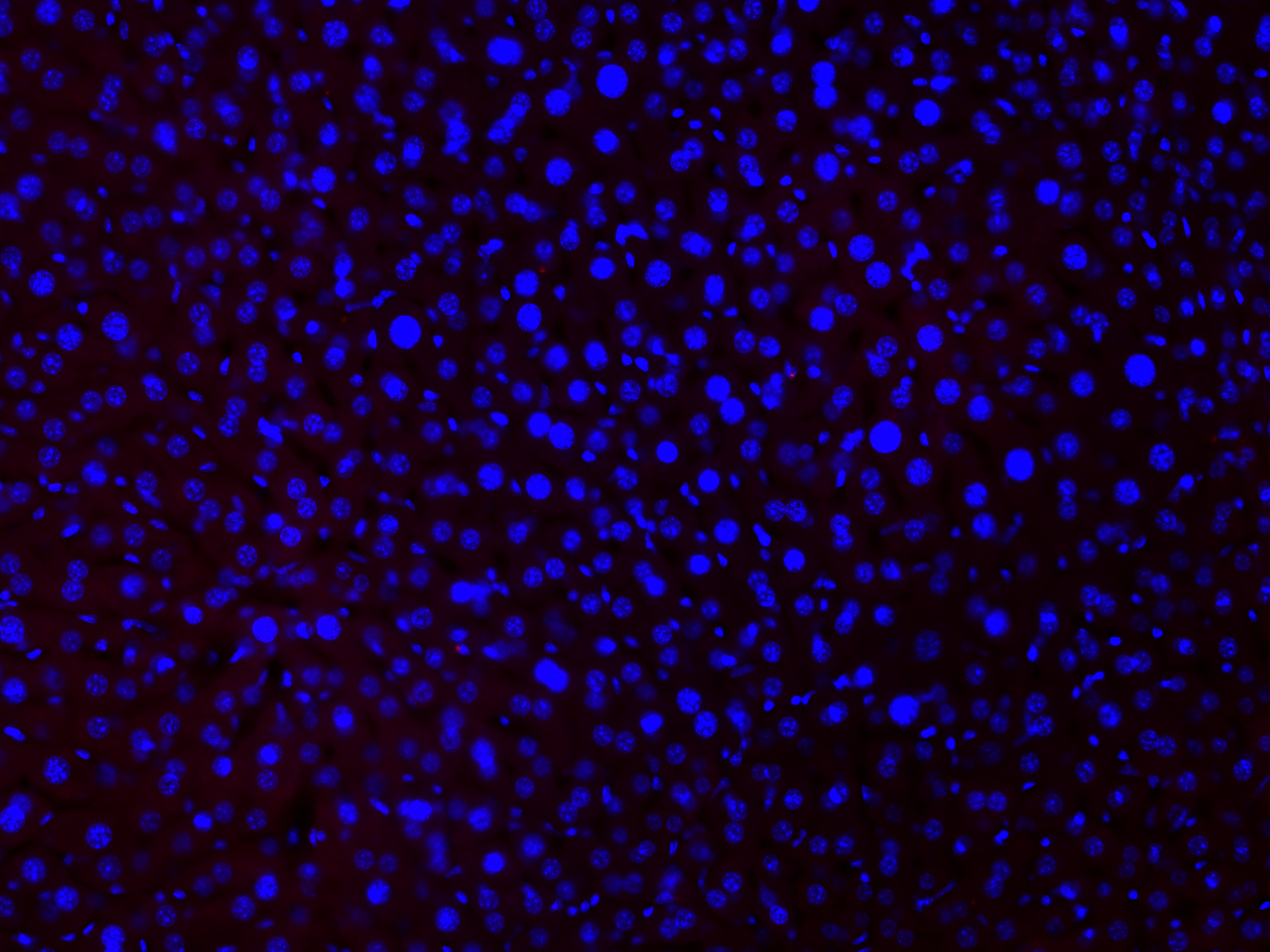

Supplement: Supplementary file 3 — Source data Fig. 2 [file 44319_2024_121_MOESM3_ESM.zip › Figure 2/I/4-KPF.tif]

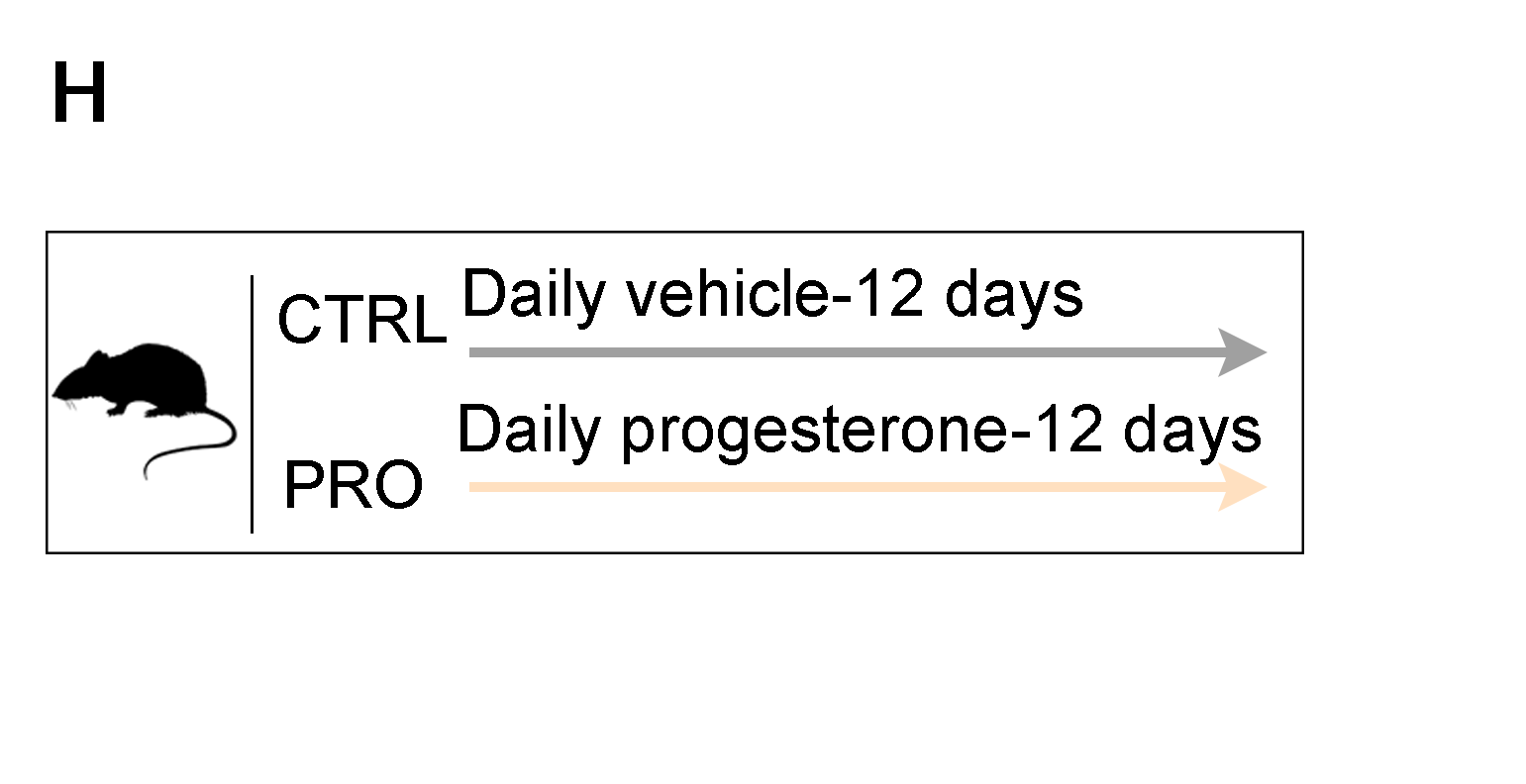

Supplement: Supplementary file 4 — Source data Fig. 3 [file 44319_2024_121_MOESM4_ESM.zip › Figure 3/H/Figure 3H.tif]

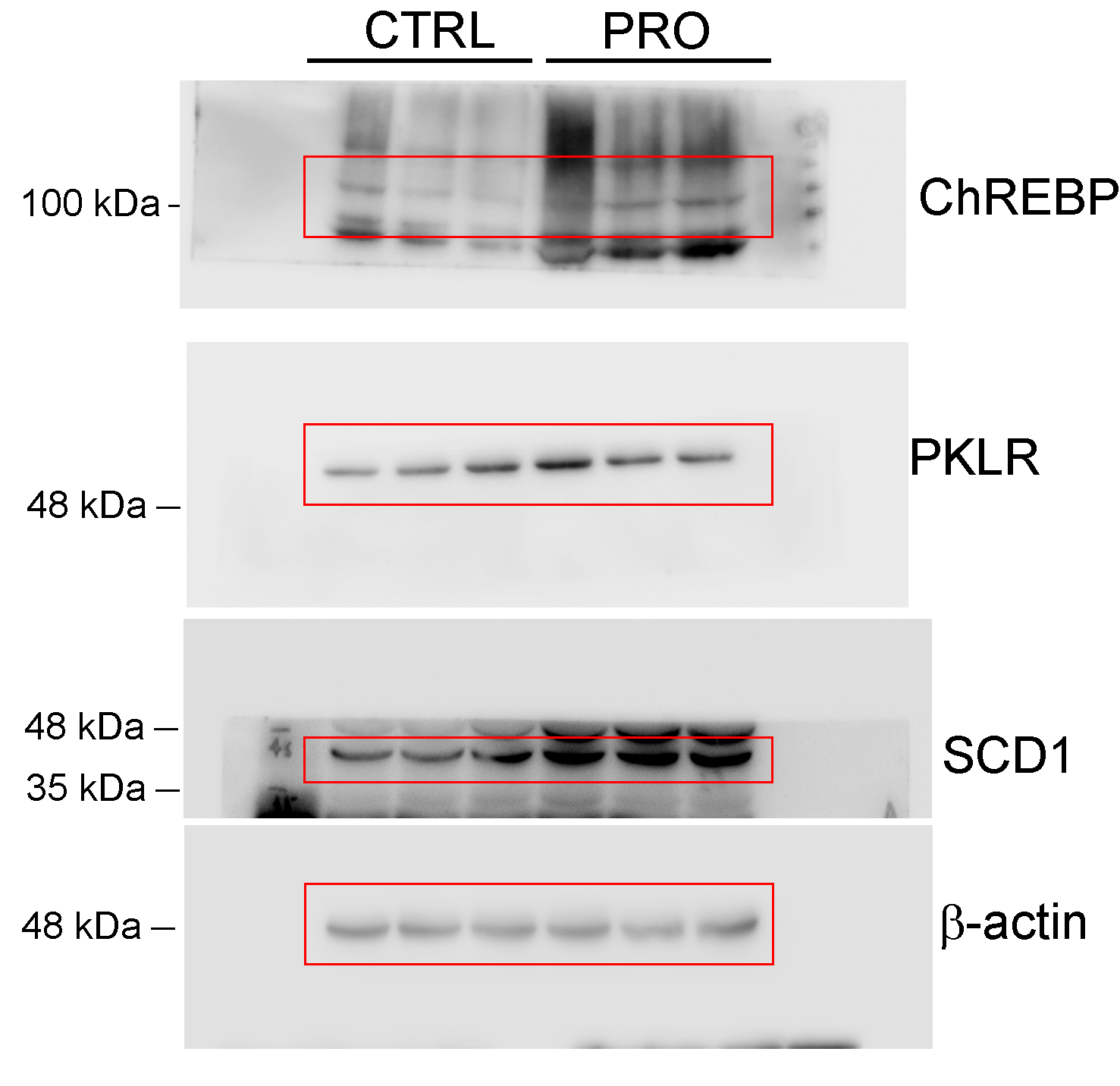

Supplement: Supplementary file 4 — Source data Fig. 3 [file 44319_2024_121_MOESM4_ESM.zip › Figure 3/K/Figure 3K.tif]

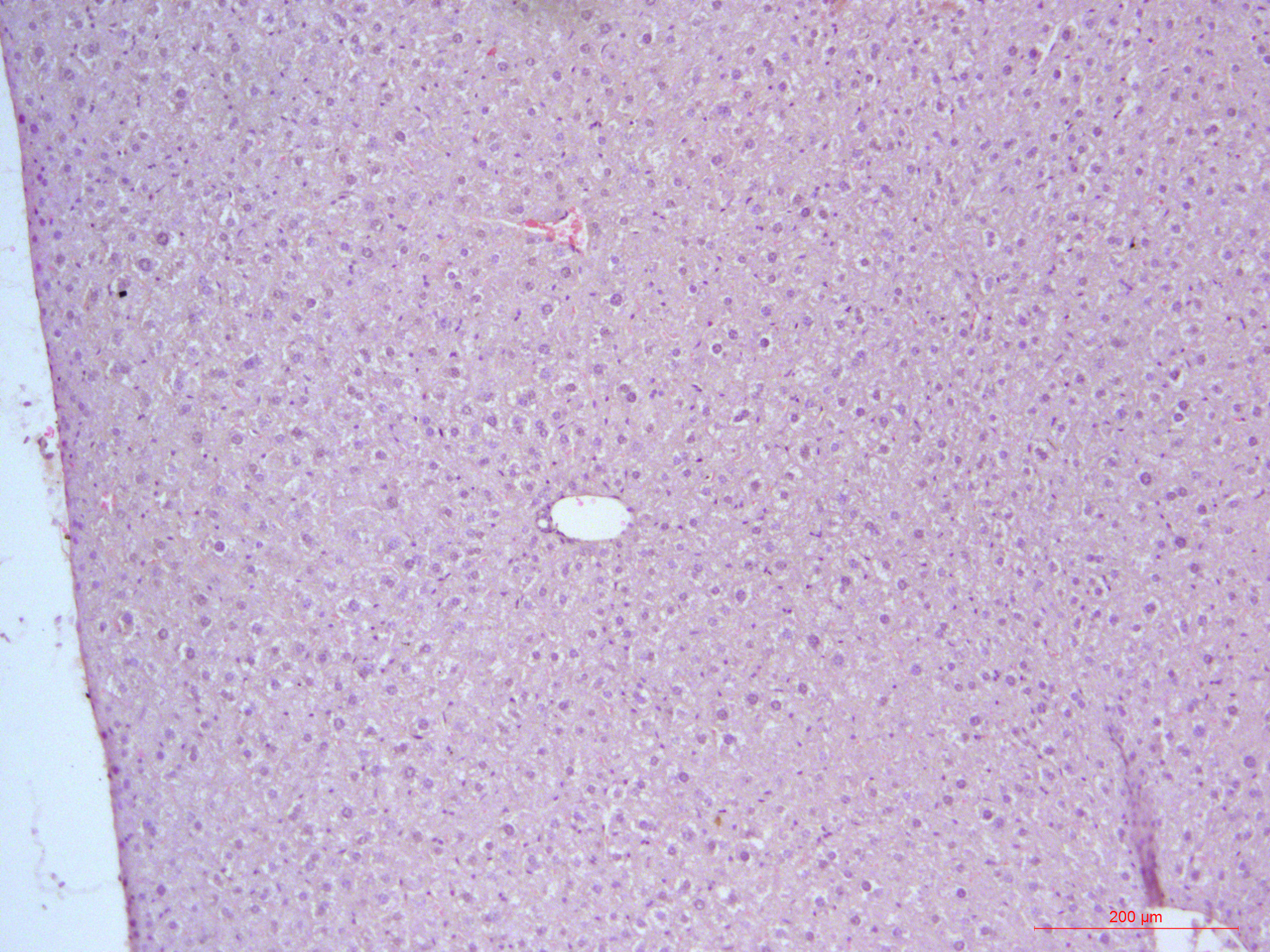

Supplement: Supplementary file 4 — Source data Fig. 3 [file 44319_2024_121_MOESM4_ESM.zip › Figure 3/N/1-HE-CTRL.tif]

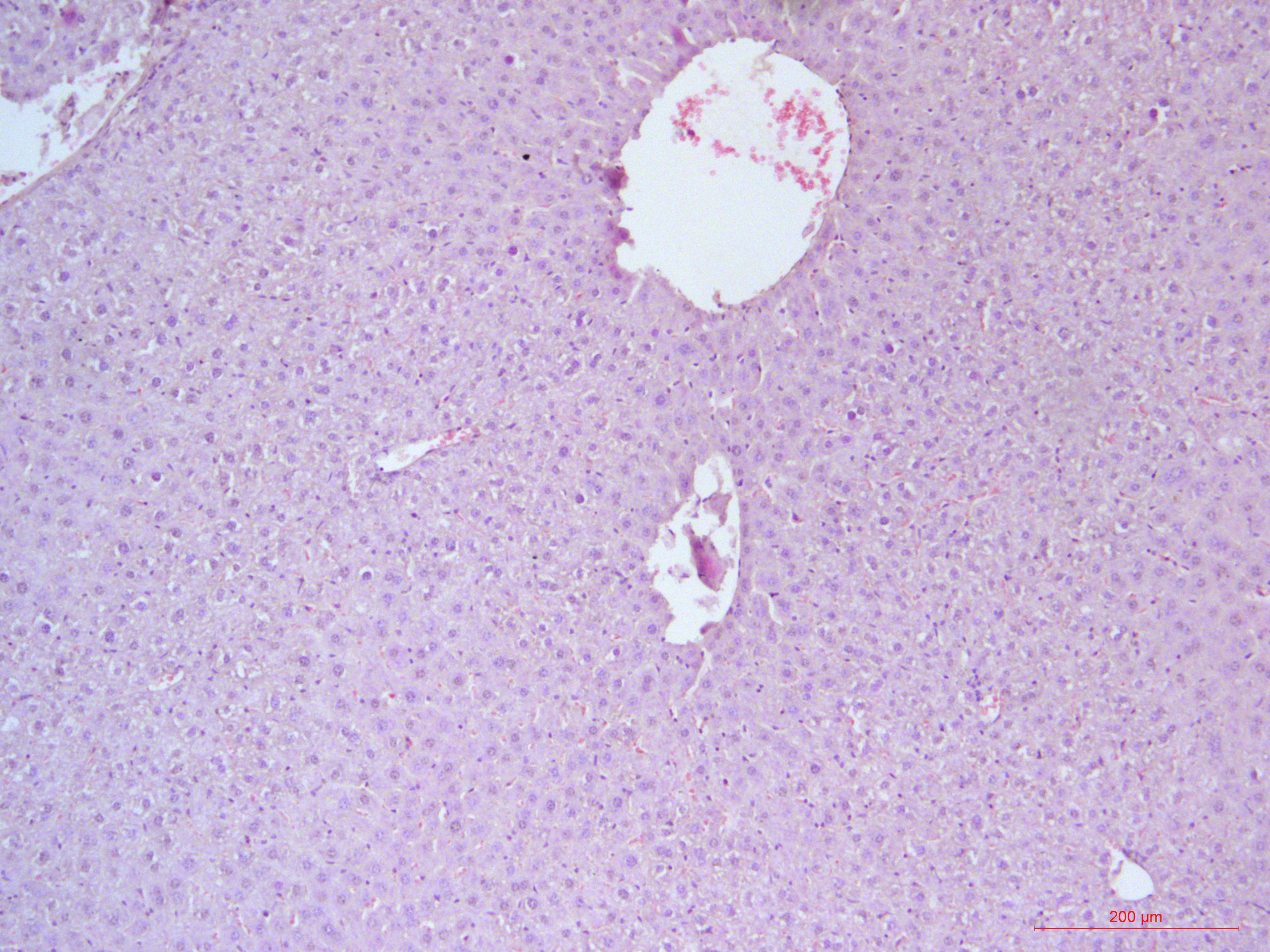

Supplement: Supplementary file 4 — Source data Fig. 3 [file 44319_2024_121_MOESM4_ESM.zip › Figure 3/N/2-HE-PRO.tif]

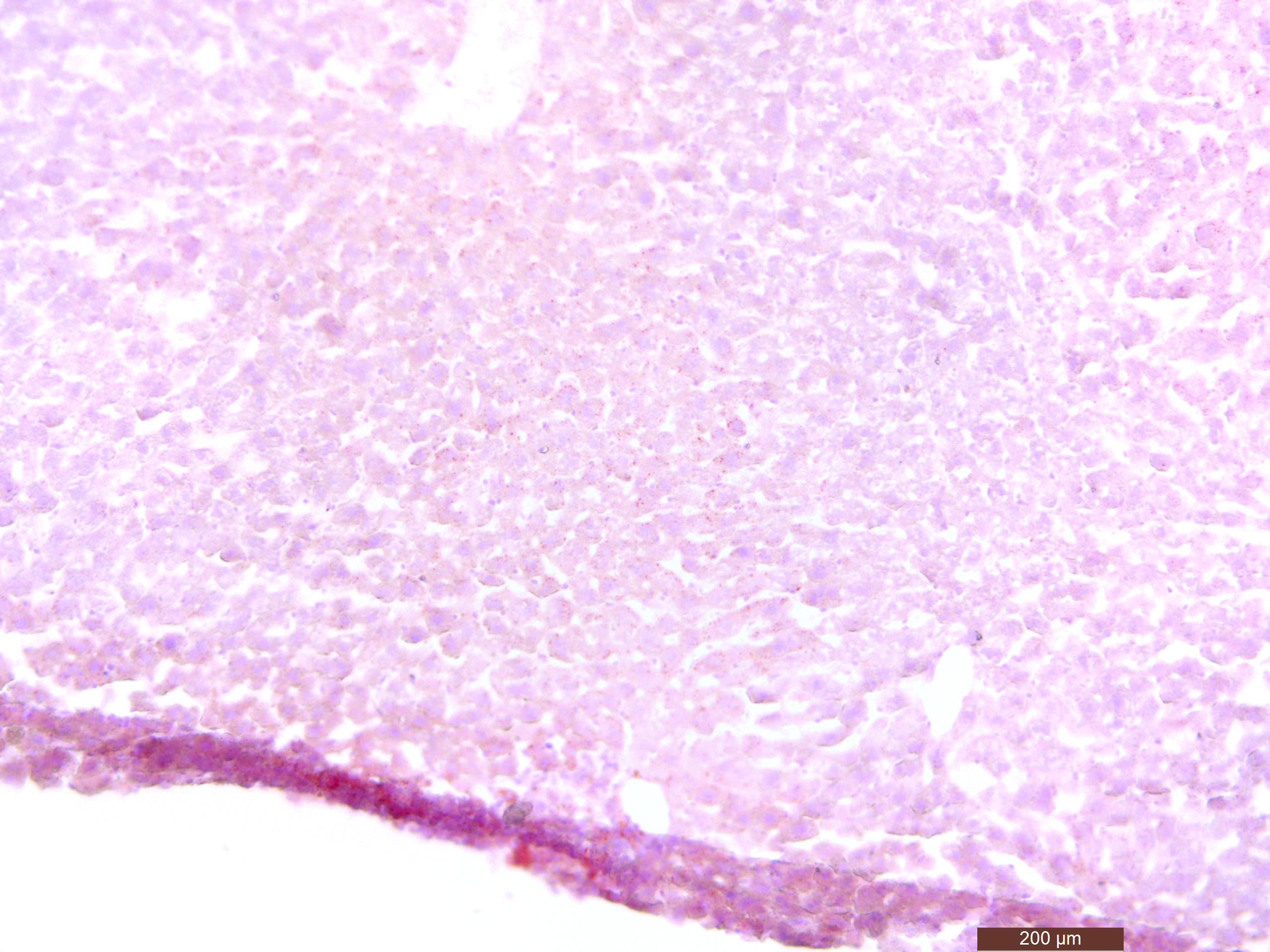

Supplement: Supplementary file 4 — Source data Fig. 3 [file 44319_2024_121_MOESM4_ESM.zip › Figure 3/N/3-ORO-CTRL.tif]

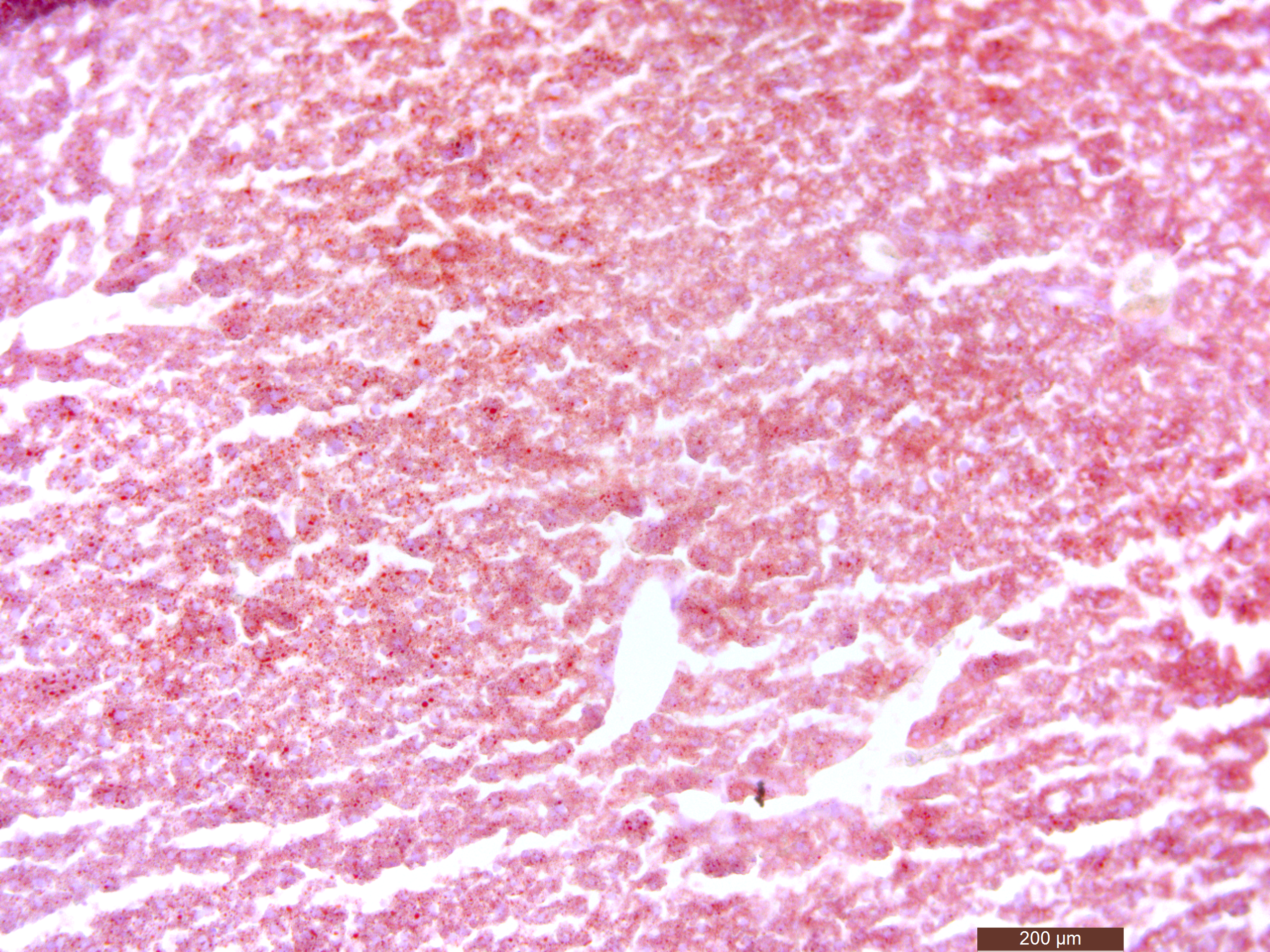

Supplement: Supplementary file 4 — Source data Fig. 3 [file 44319_2024_121_MOESM4_ESM.zip › Figure 3/N/4-ORO-PRO.tif]

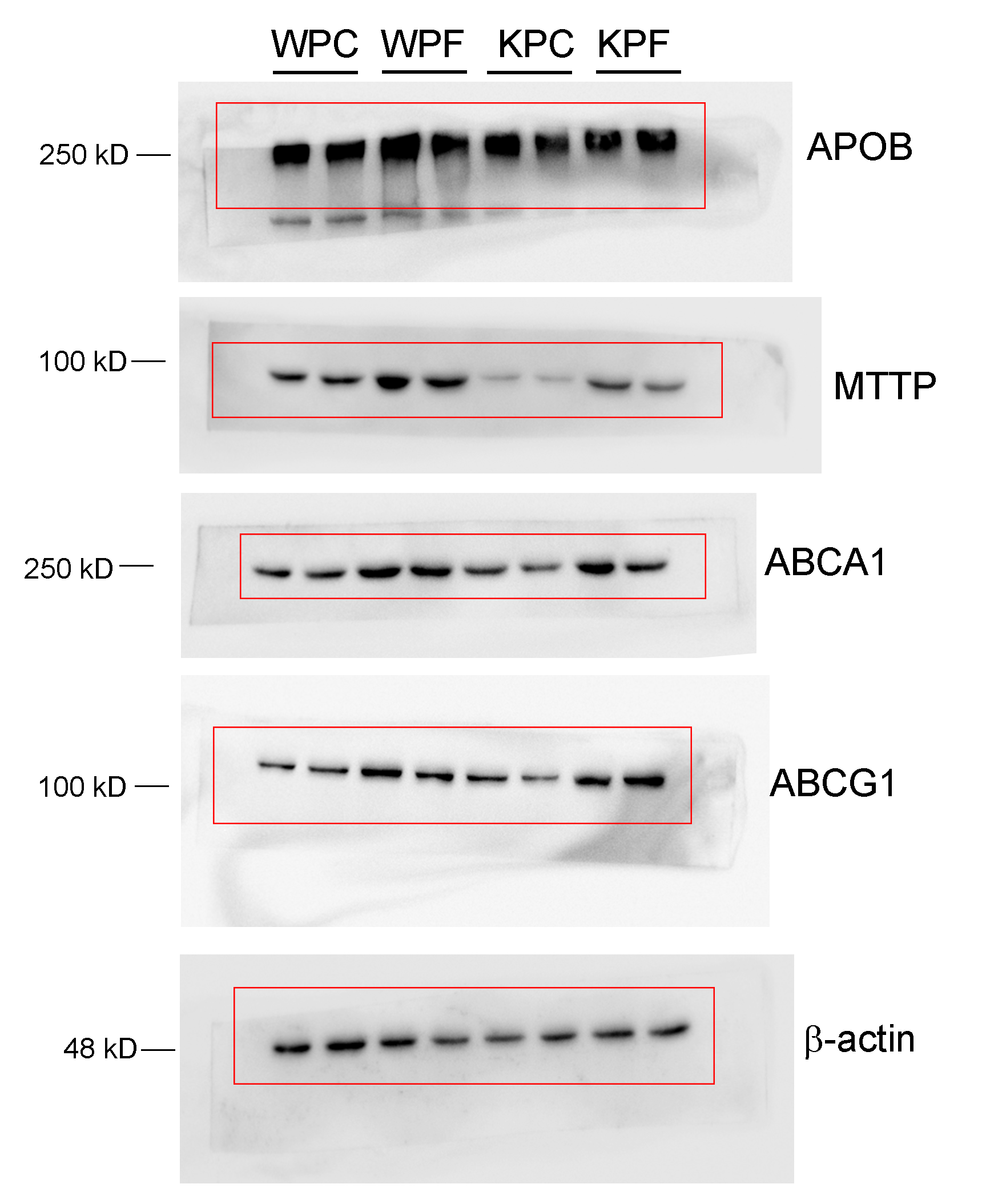

Supplement: Supplementary file 5 — Source data Fig. 4 [file 44319_2024_121_MOESM5_ESM.zip › Figure 4/E/Figure 4E.tif]

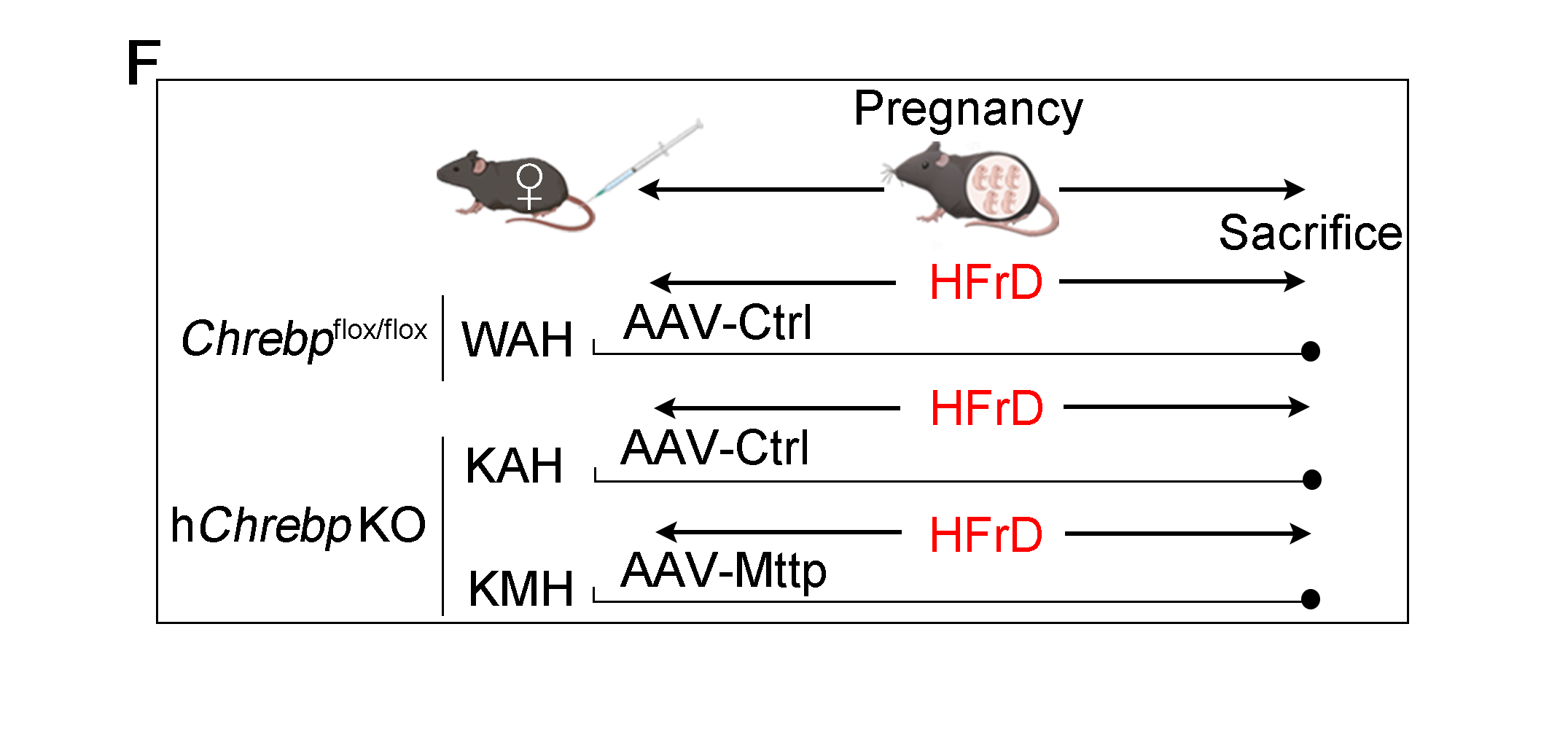

Supplement: Supplementary file 5 — Source data Fig. 4 [file 44319_2024_121_MOESM5_ESM.zip › Figure 4/F/Figure 4F.tif]

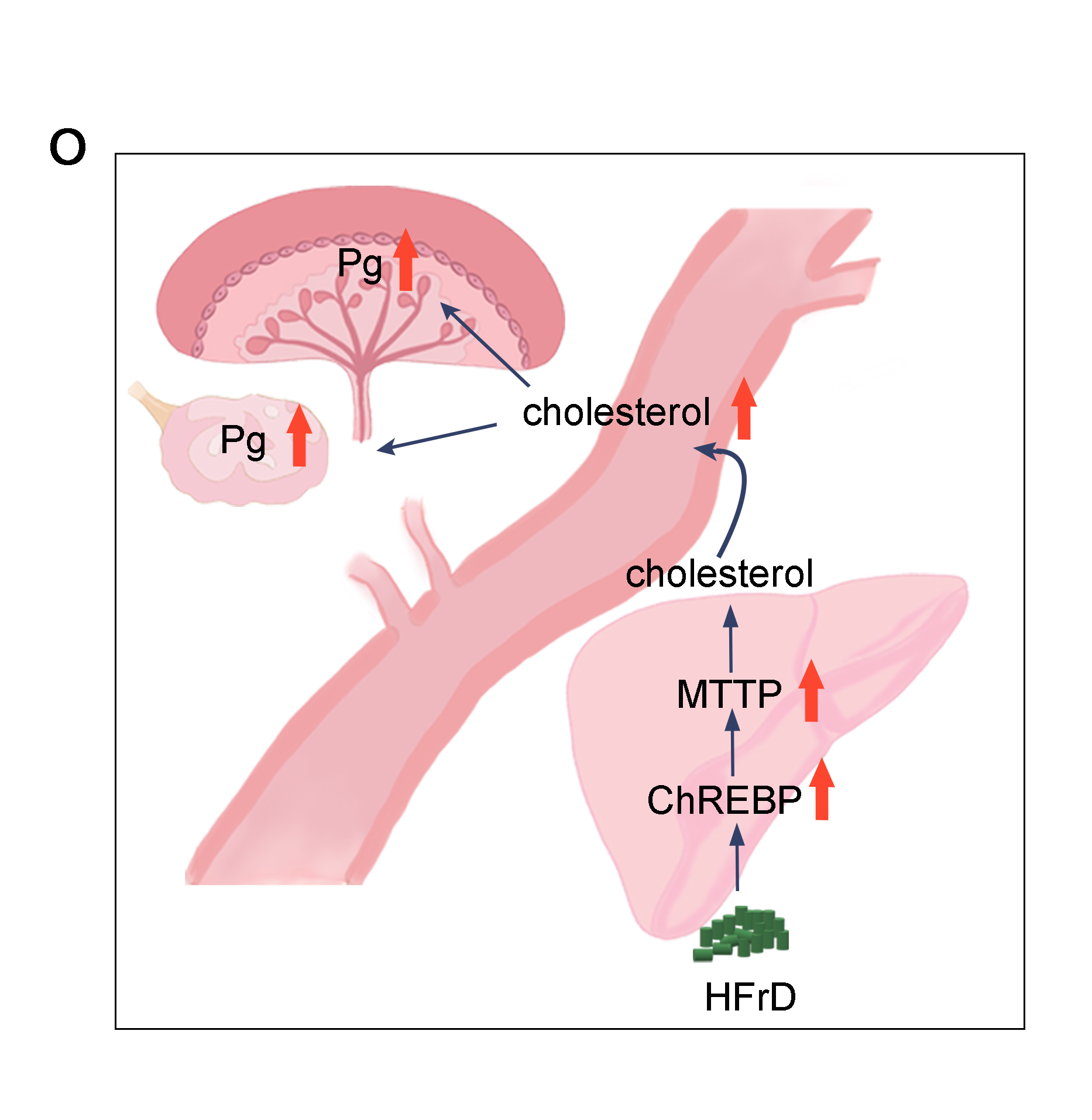

Supplement: Supplementary file 5 — Source data Fig. 4 [file 44319_2024_121_MOESM5_ESM.zip › Figure 4/O/Figure 4O.tif]

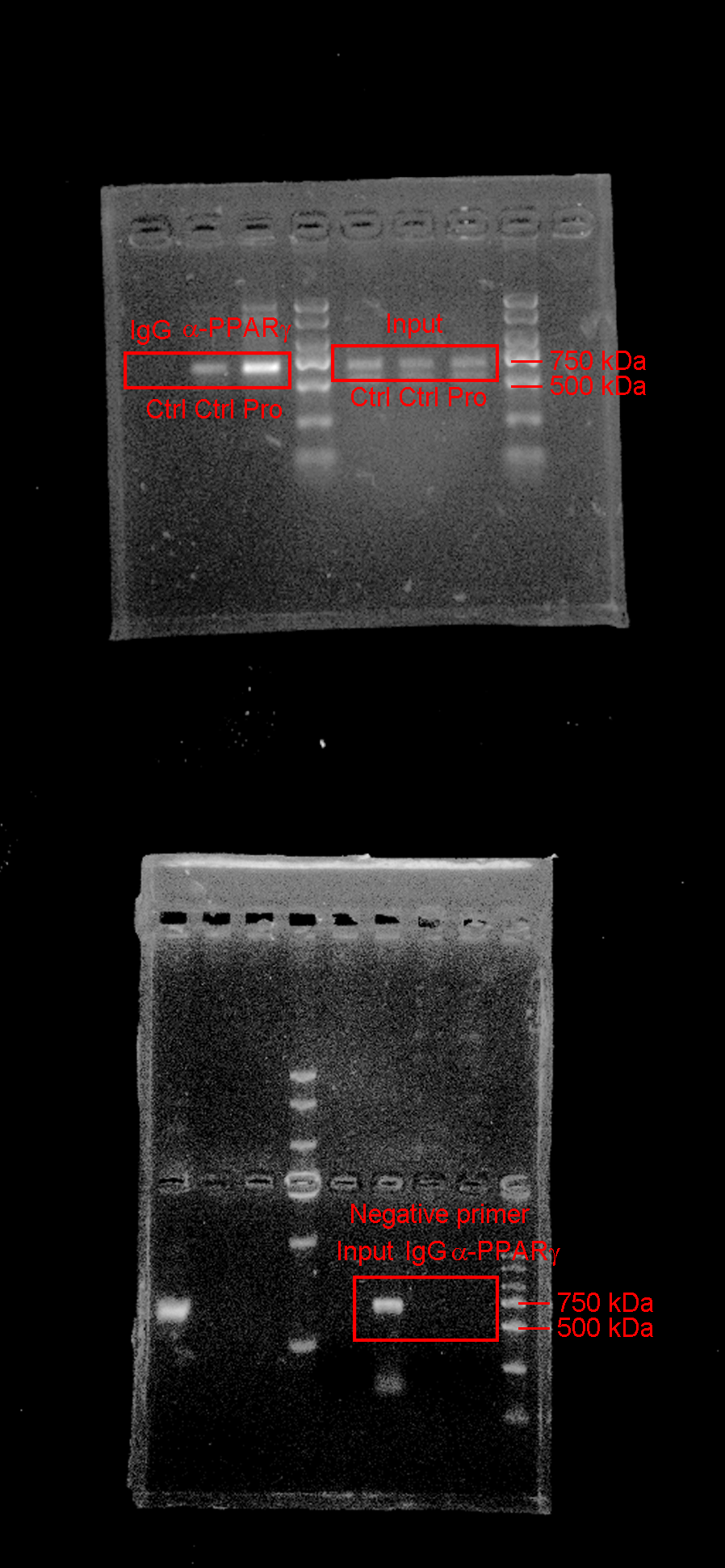

Supplement: Supplementary file 6 — Source data Fig. 5 [file 44319_2024_121_MOESM6_ESM.zip › Figure 5/C/left.tif]

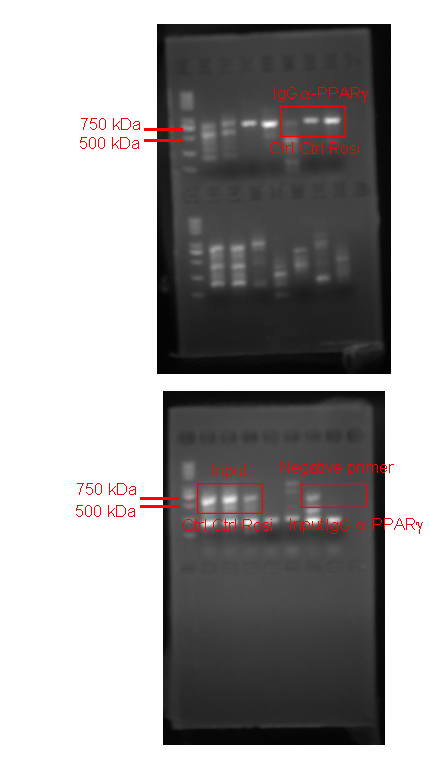

Supplement: Supplementary file 6 — Source data Fig. 5 [file 44319_2024_121_MOESM6_ESM.zip › Figure 5/C/right.tif]

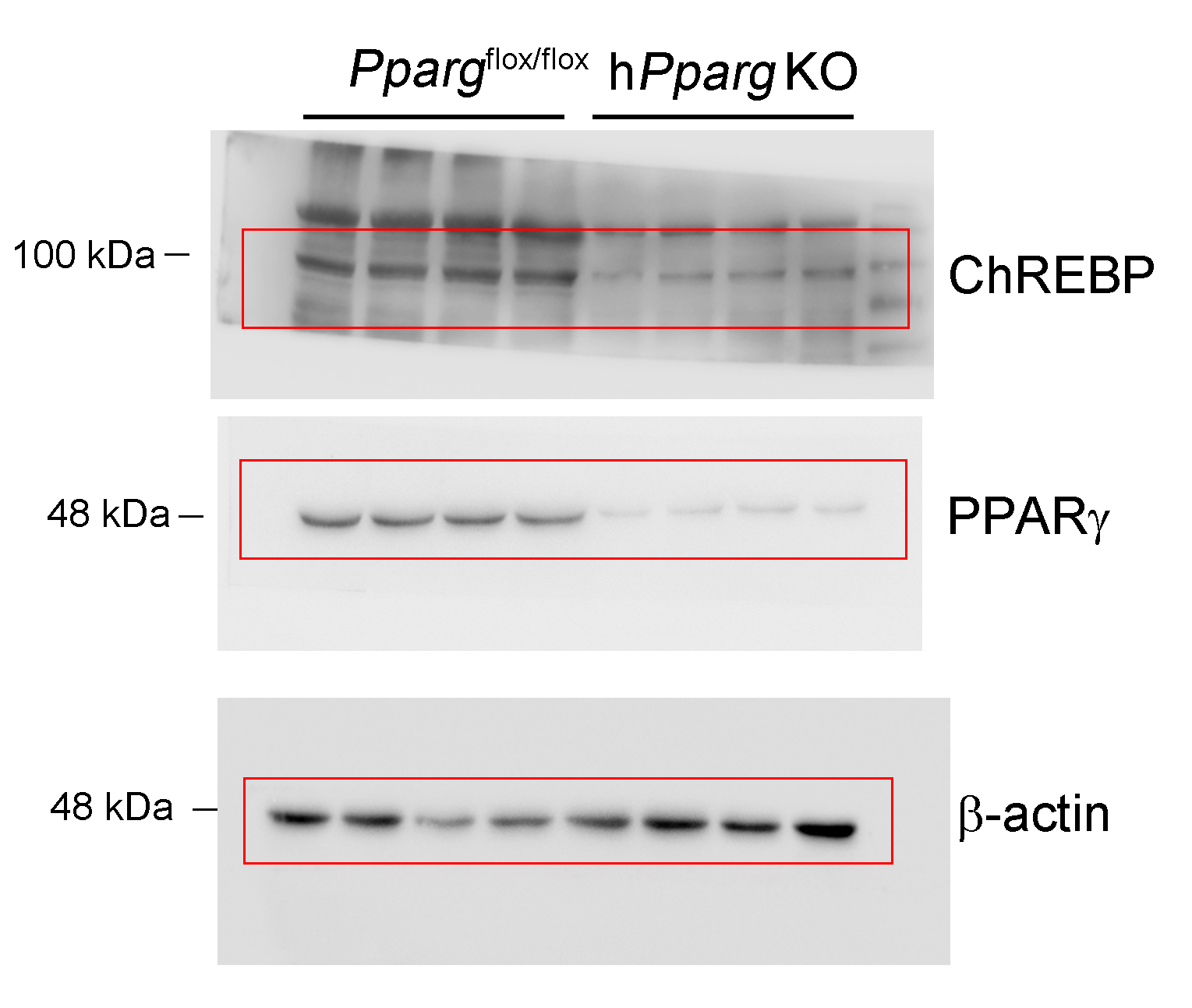

Supplement: Supplementary file 6 — Source data Fig. 5 [file 44319_2024_121_MOESM6_ESM.zip › Figure 5/D/Figure 5D.tif]

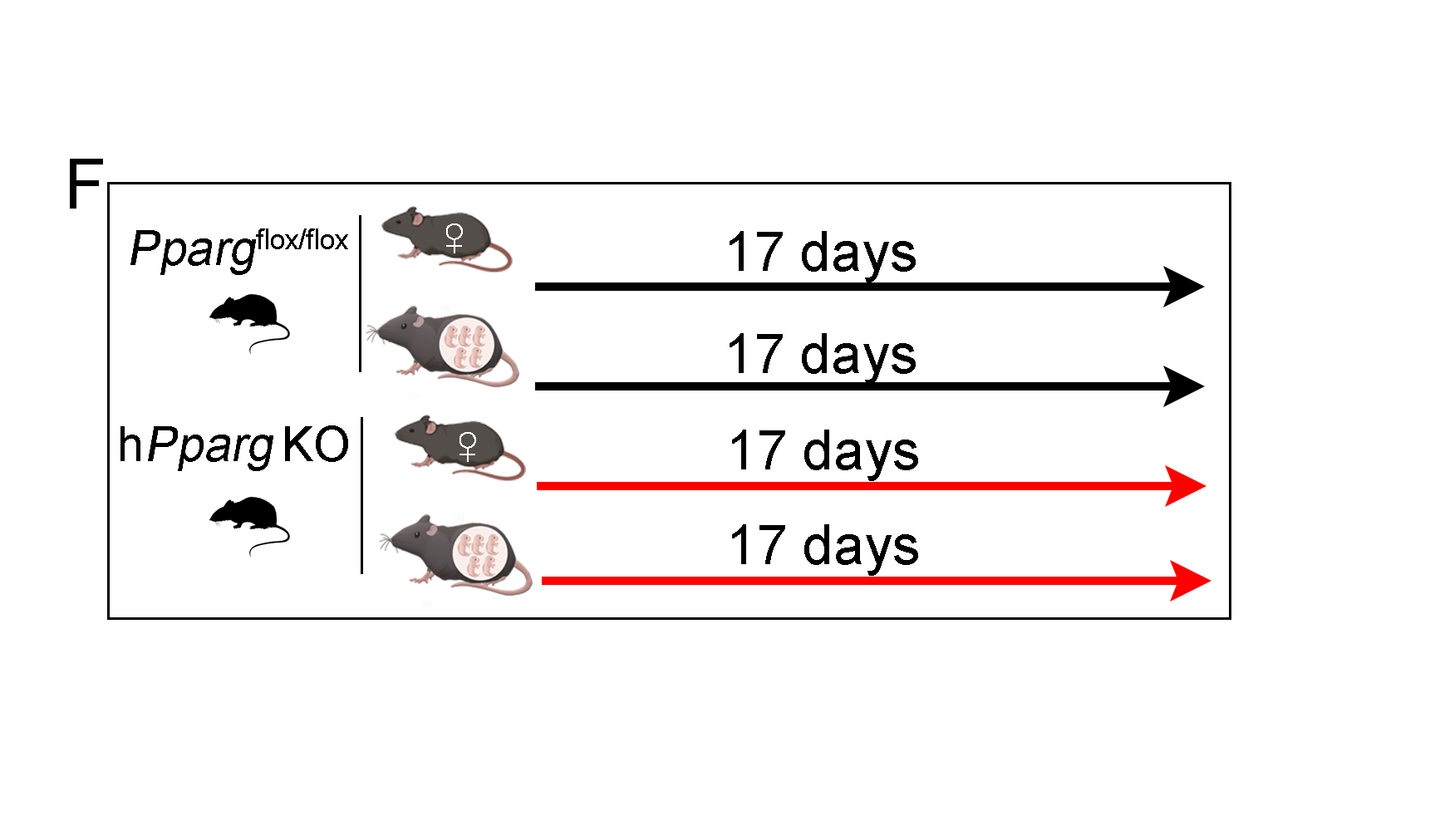

Supplement: Supplementary file 6 — Source data Fig. 5 [file 44319_2024_121_MOESM6_ESM.zip › Figure 5/F/Figure 5F.tif]

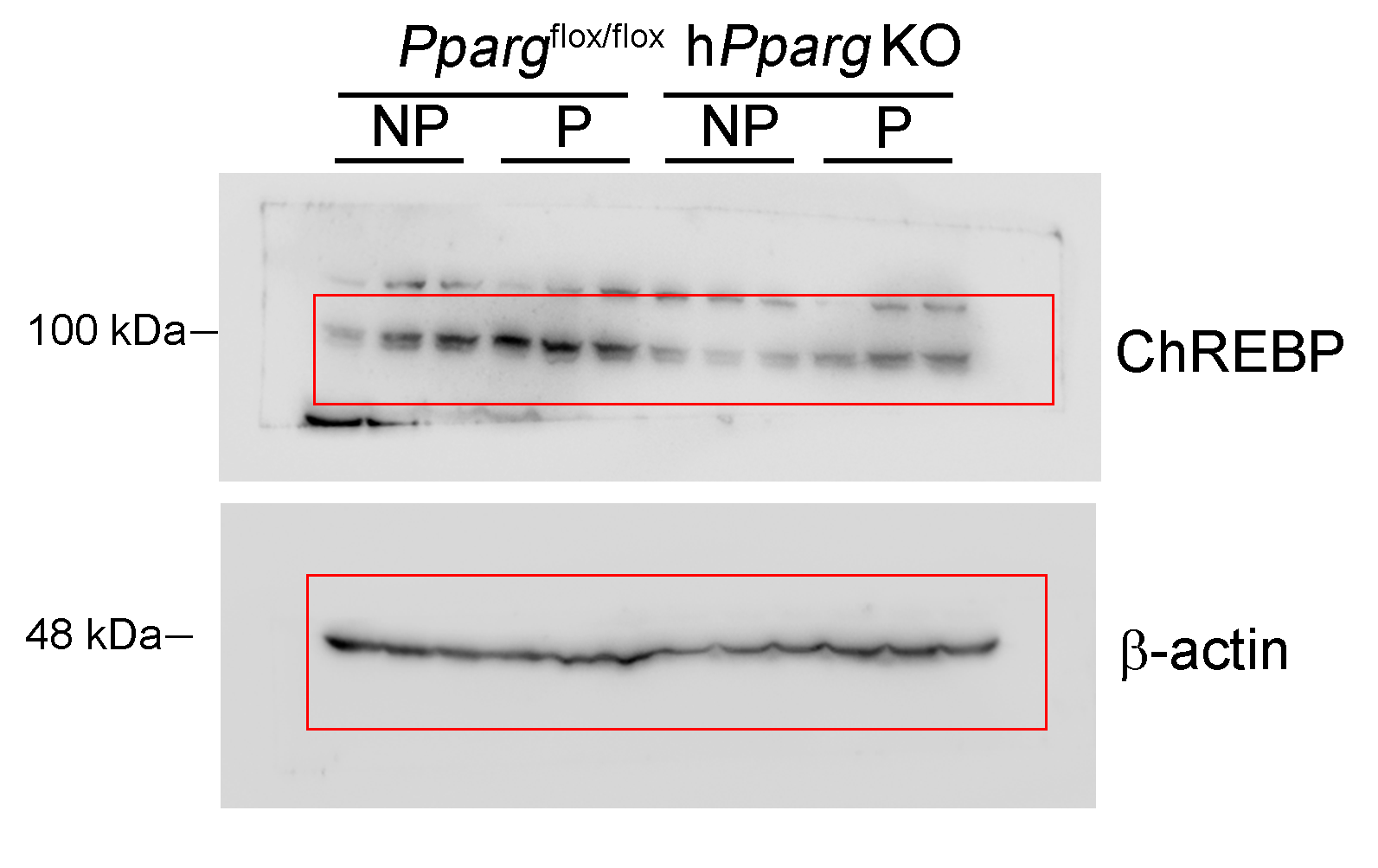

Supplement: Supplementary file 6 — Source data Fig. 5 [file 44319_2024_121_MOESM6_ESM.zip › Figure 5/H/Figure 5H.tif]

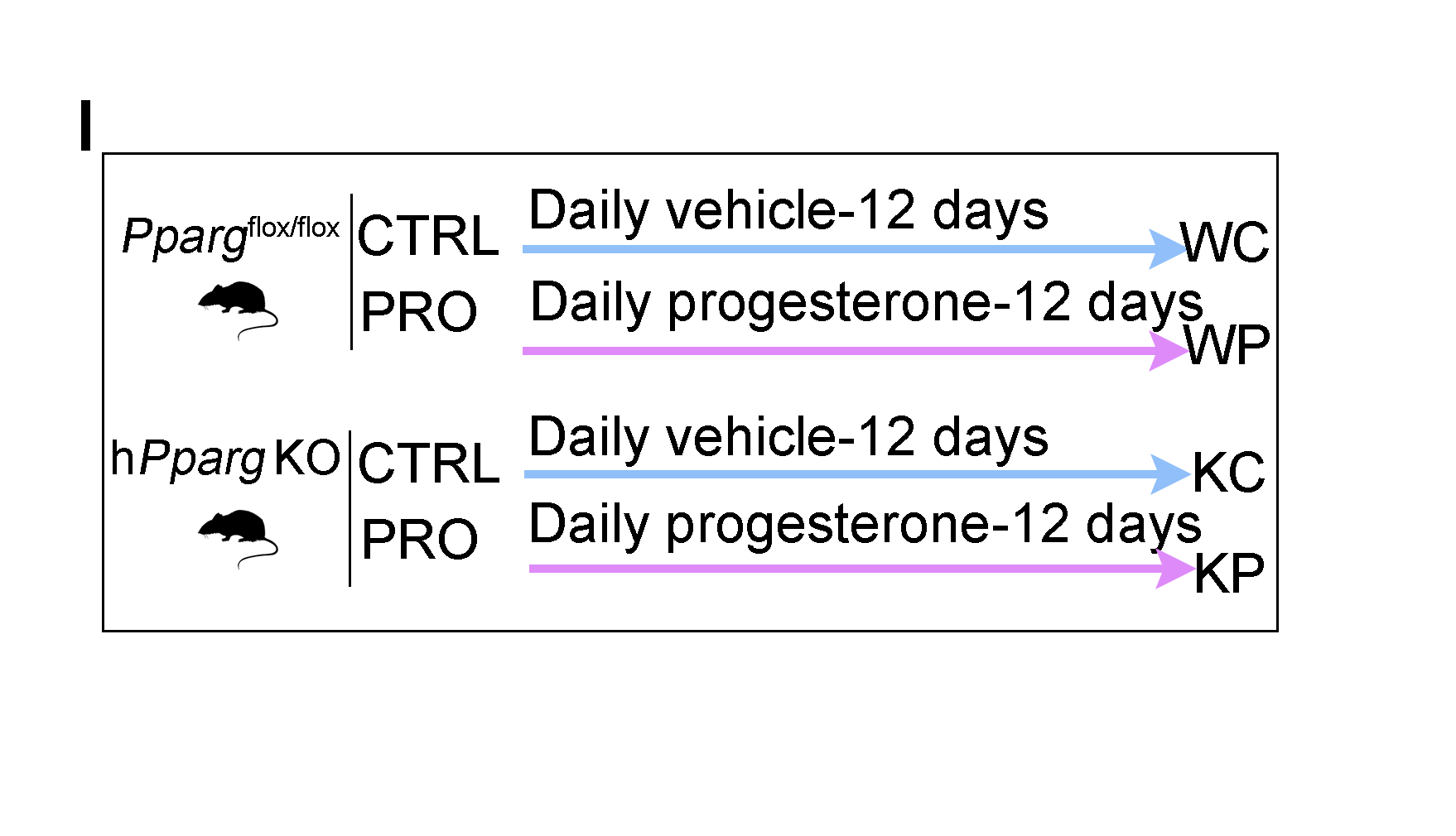

Supplement: Supplementary file 6 — Source data Fig. 5 [file 44319_2024_121_MOESM6_ESM.zip › Figure 5/I/Figure 5.tif]

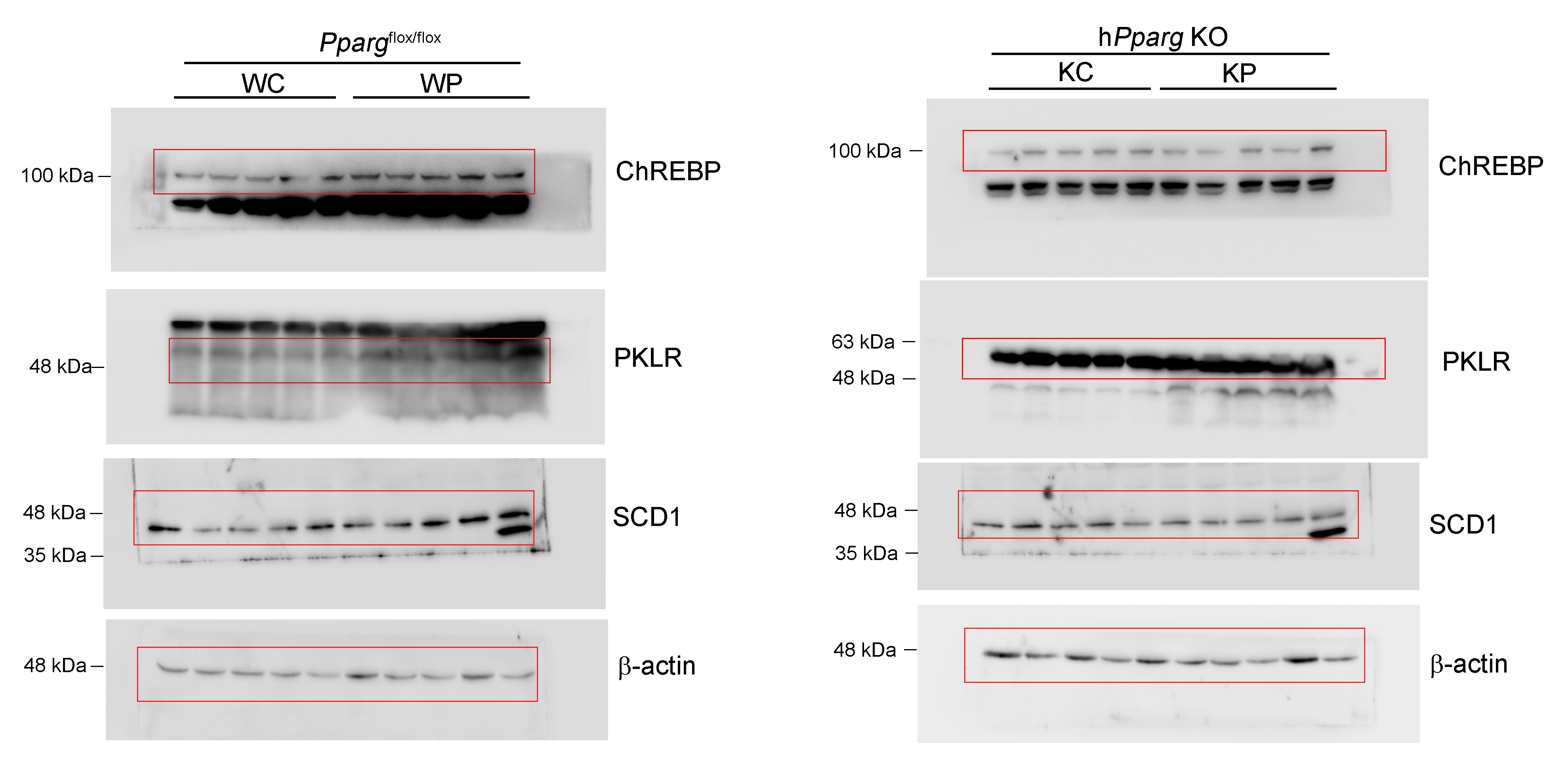

Supplement: Supplementary file 6 — Source data Fig. 5 [file 44319_2024_121_MOESM6_ESM.zip › Figure 5/L/Figure 5L.tif]

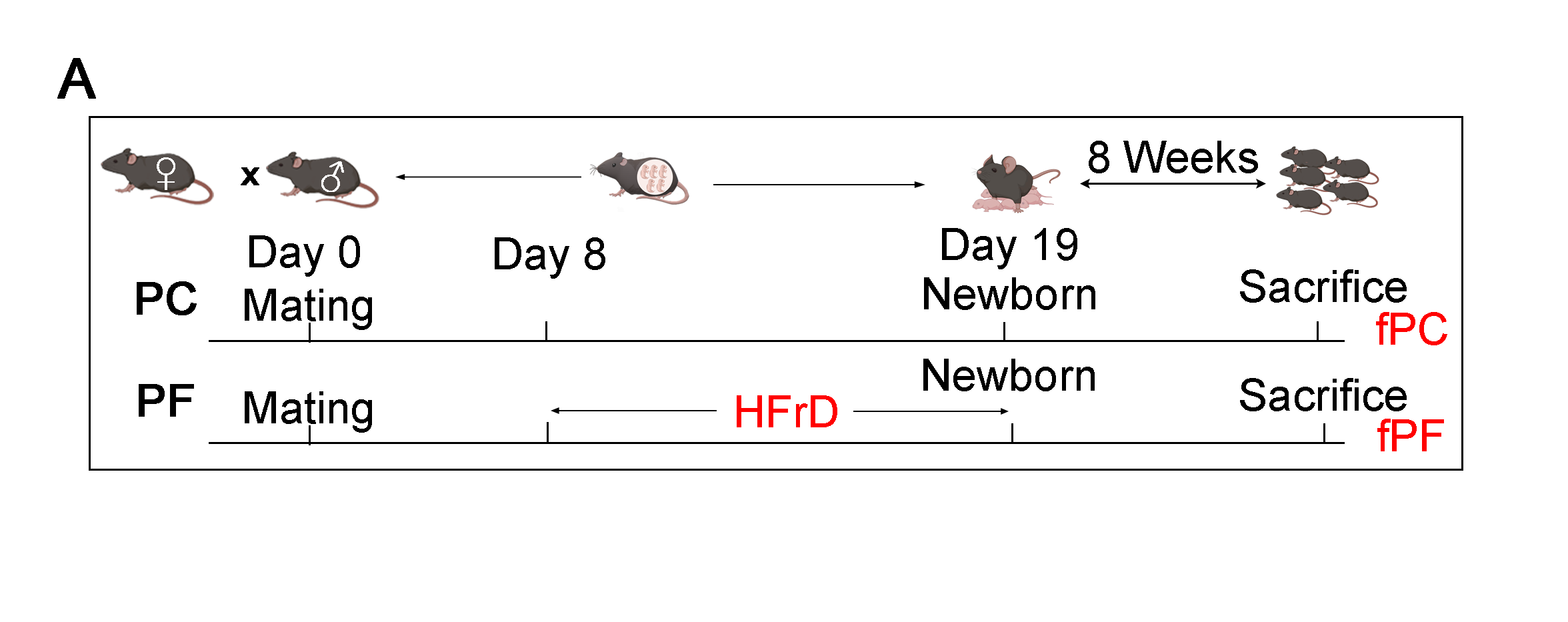

Supplement: Supplementary file 7 — Source data Fig. 6 [file 44319_2024_121_MOESM7_ESM.zip › Figure 6/A/Figure 6A.tif]

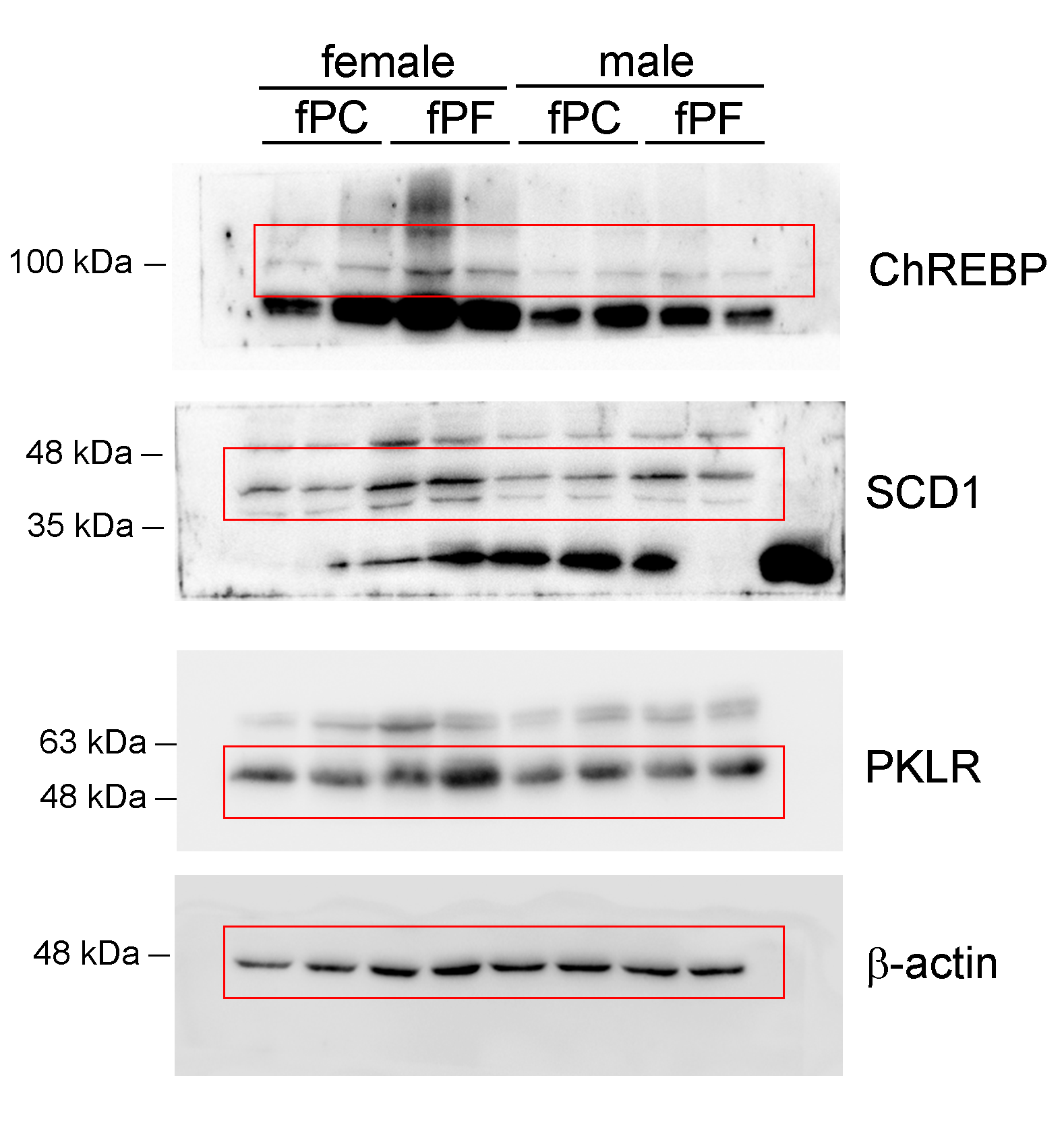

Supplement: Supplementary file 7 — Source data Fig. 6 [file 44319_2024_121_MOESM7_ESM.zip › Figure 6/I/Figure 6I.tif]

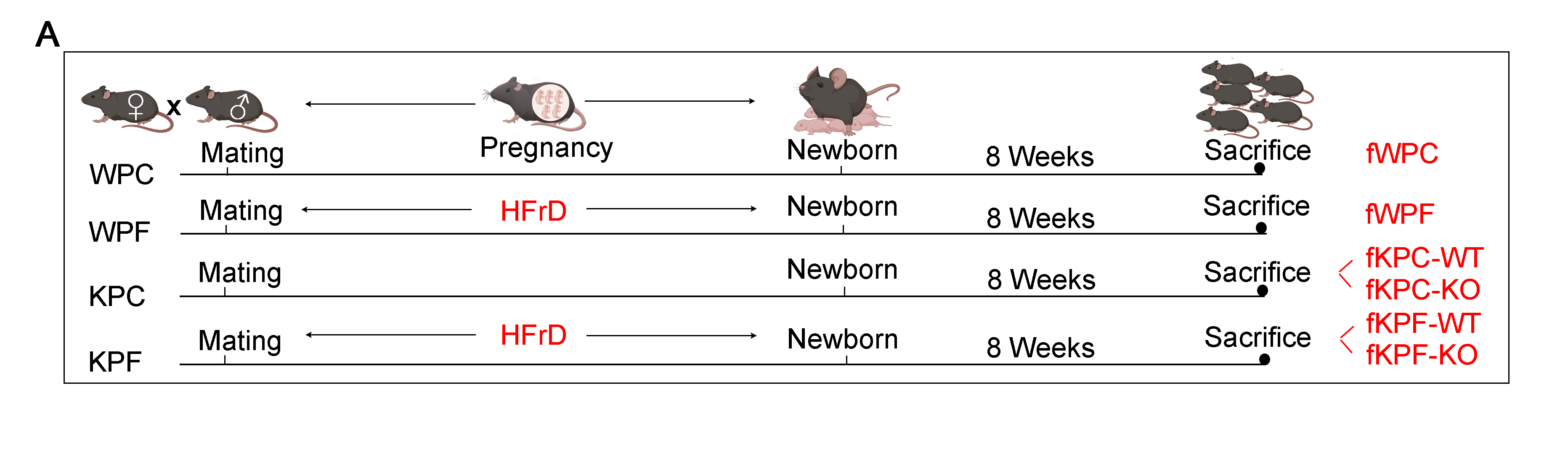

Supplement: Supplementary file 8 — Source data Fig. 7 [file 44319_2024_121_MOESM8_ESM.zip › Figure 7/A/Figure 7A.tif]

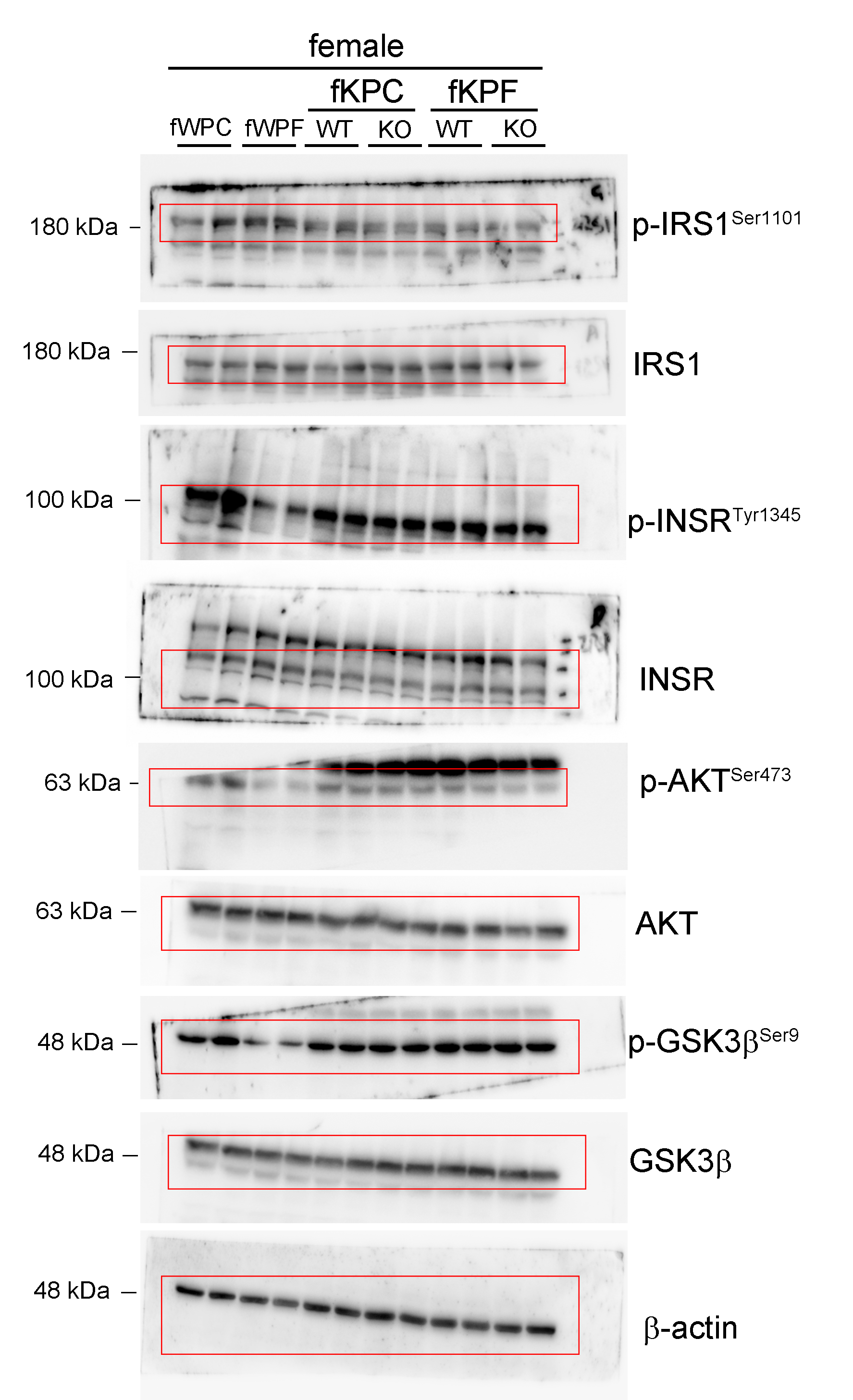

Supplement: Supplementary file 8 — Source data Fig. 7 [file 44319_2024_121_MOESM8_ESM.zip › Figure 7/E/Figure 7E.tif]

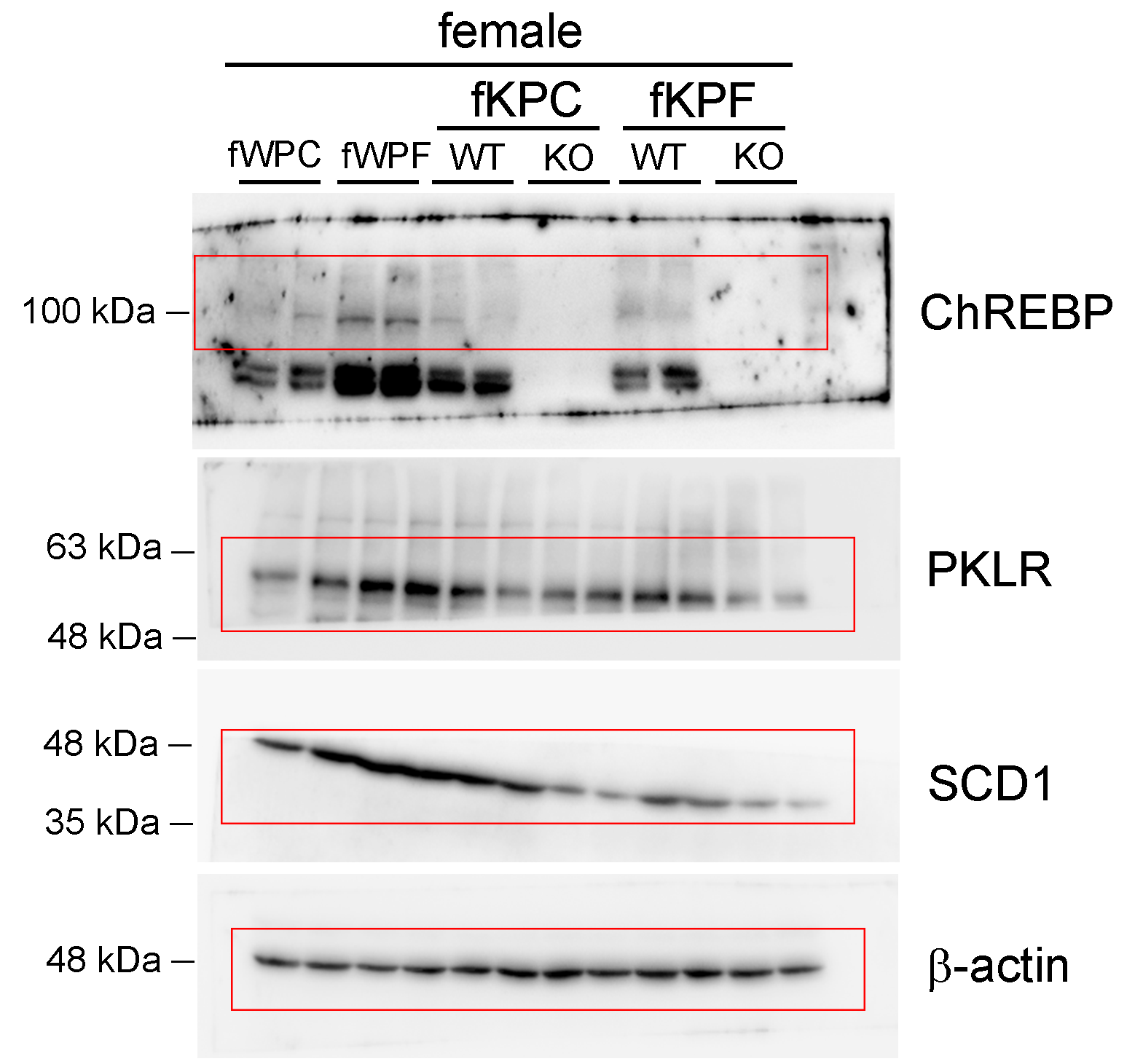

Supplement: Supplementary file 8 — Source data Fig. 7 [file 44319_2024_121_MOESM8_ESM.zip › Figure 7/G/Figure 7G.tif]

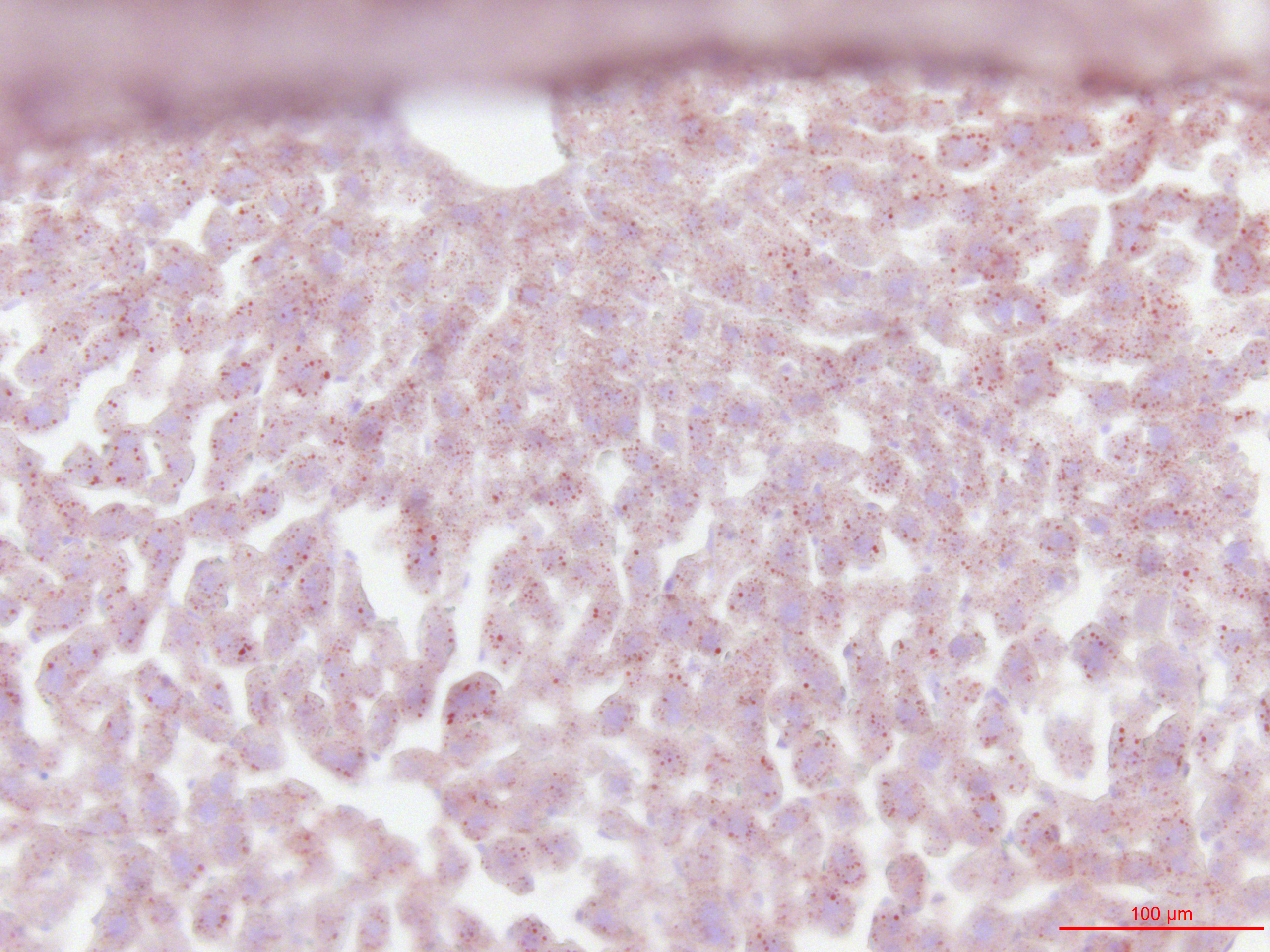

Supplement: Supplementary file 8 — Source data Fig. 7 [file 44319_2024_121_MOESM8_ESM.zip › Figure 7/L/1-fWPC.tif]

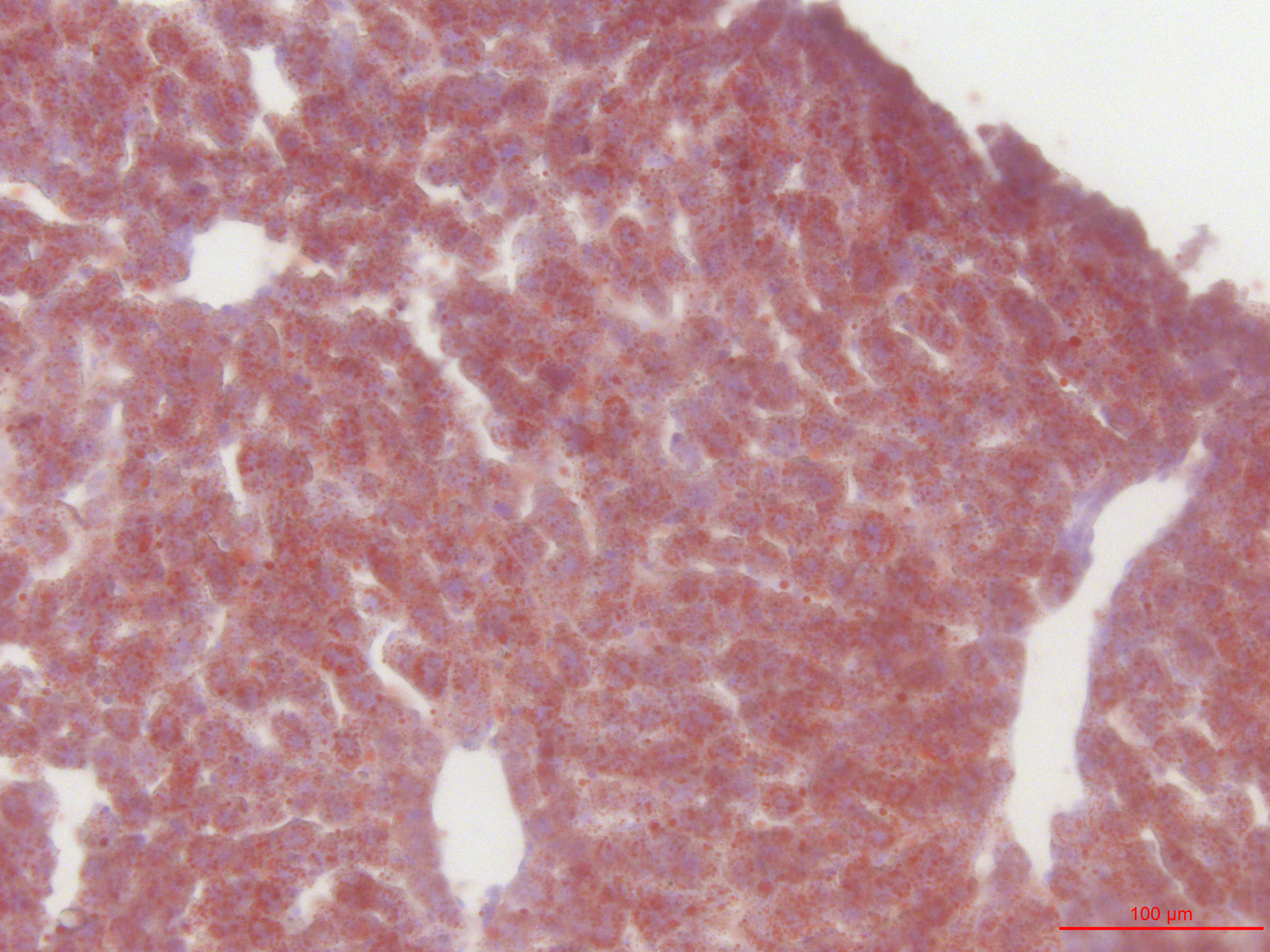

Supplement: Supplementary file 8 — Source data Fig. 7 [file 44319_2024_121_MOESM8_ESM.zip › Figure 7/L/2-fWPF.tif]

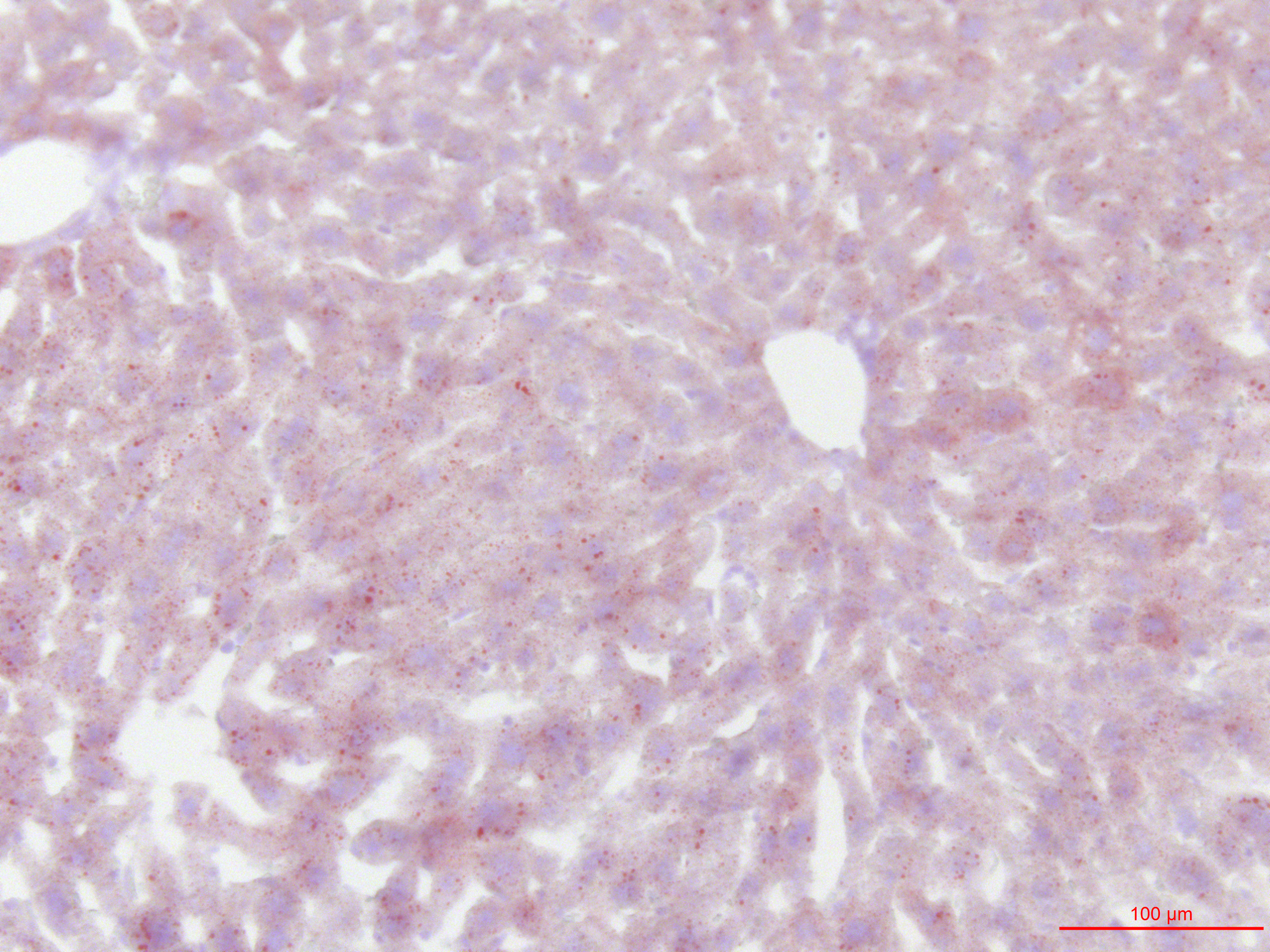

Supplement: Supplementary file 8 — Source data Fig. 7 [file 44319_2024_121_MOESM8_ESM.zip › Figure 7/L/3-fKPC-WT.tif]

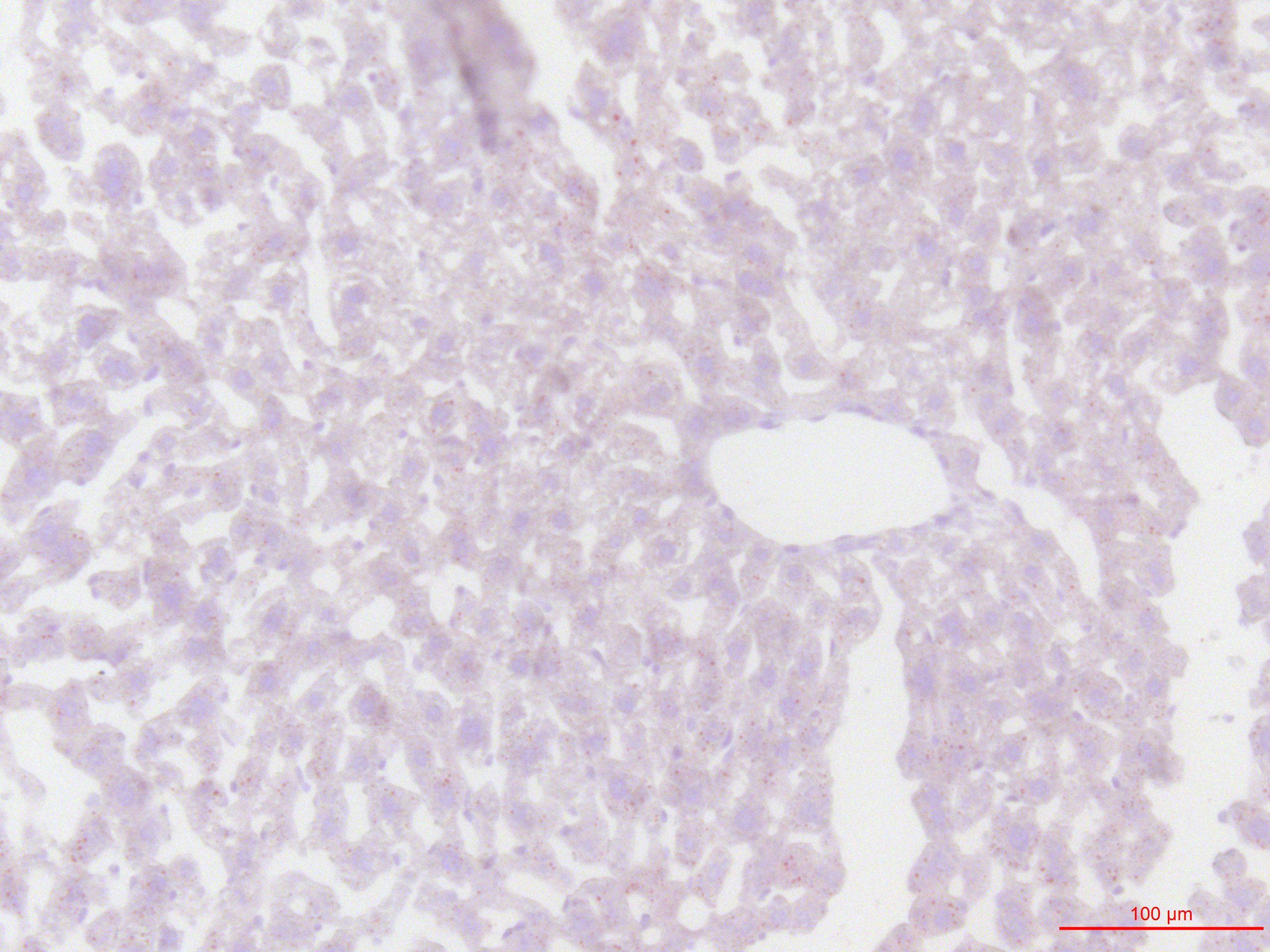

Supplement: Supplementary file 8 — Source data Fig. 7 [file 44319_2024_121_MOESM8_ESM.zip › Figure 7/L/4-fKPC-KO.tif]

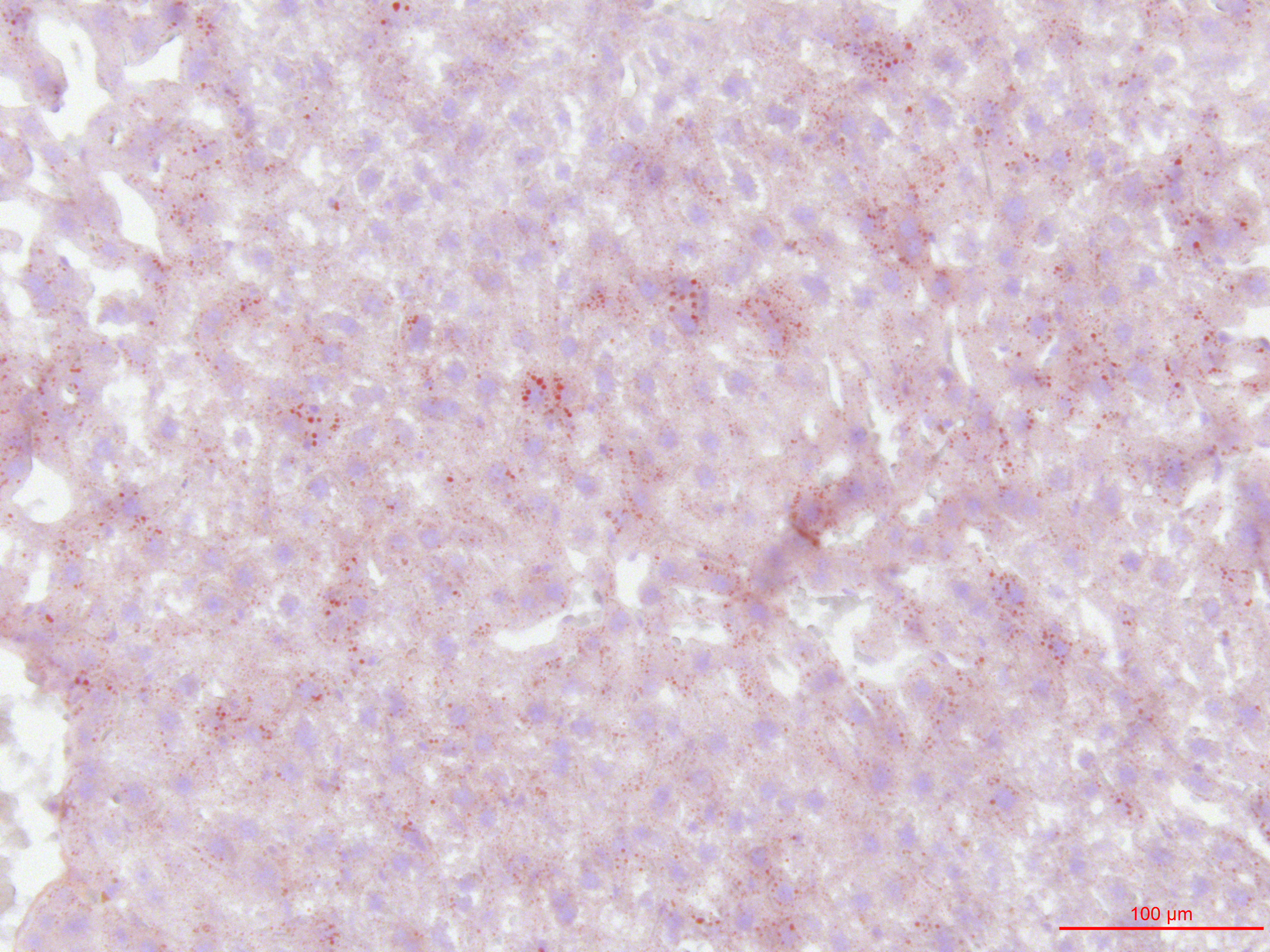

Supplement: Supplementary file 8 — Source data Fig. 7 [file 44319_2024_121_MOESM8_ESM.zip › Figure 7/L/5-fKPF-WT.tif]

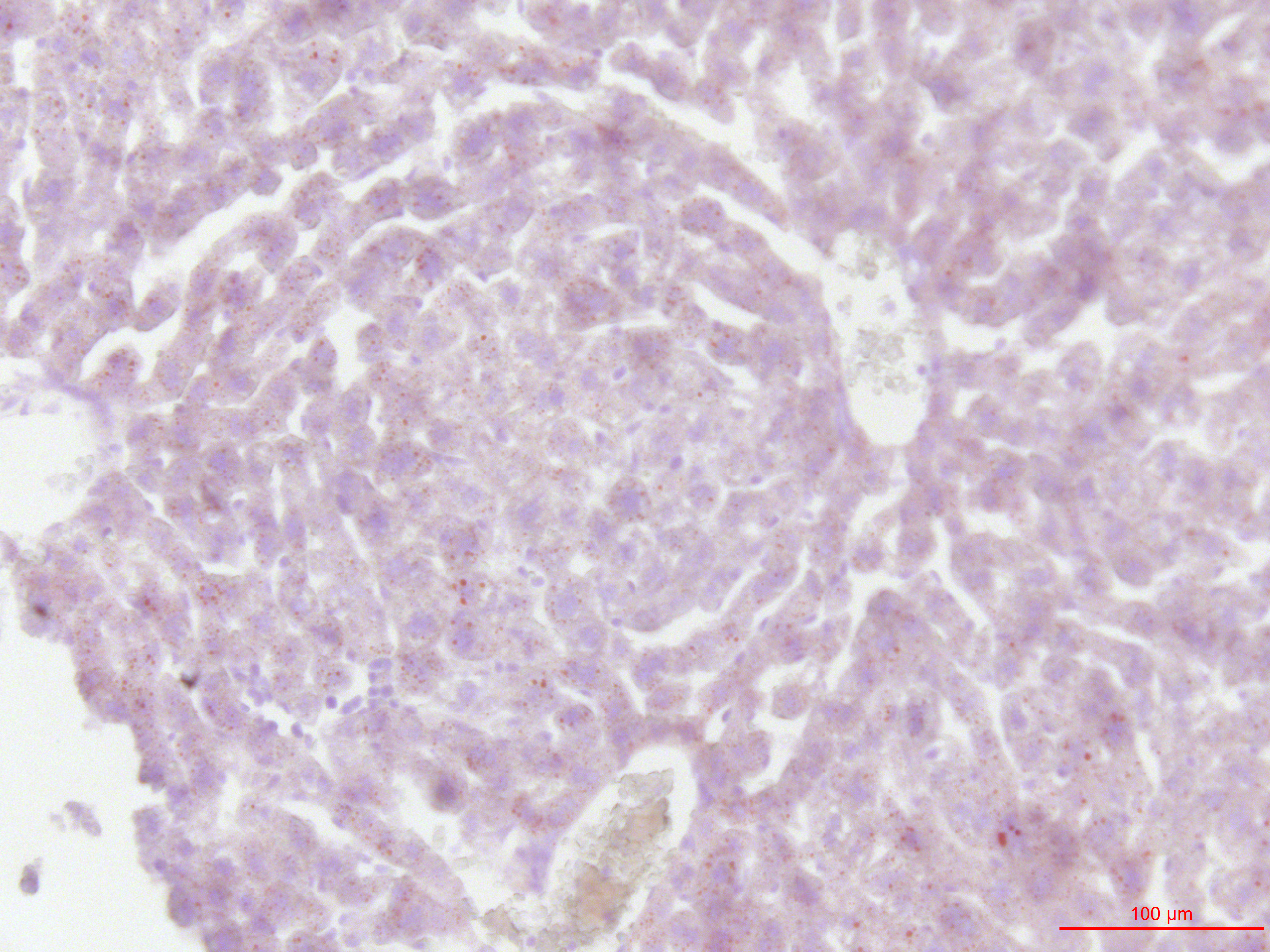

Supplement: Supplementary file 8 — Source data Fig. 7 [file 44319_2024_121_MOESM8_ESM.zip › Figure 7/L/6-fKPF-KO.tif]

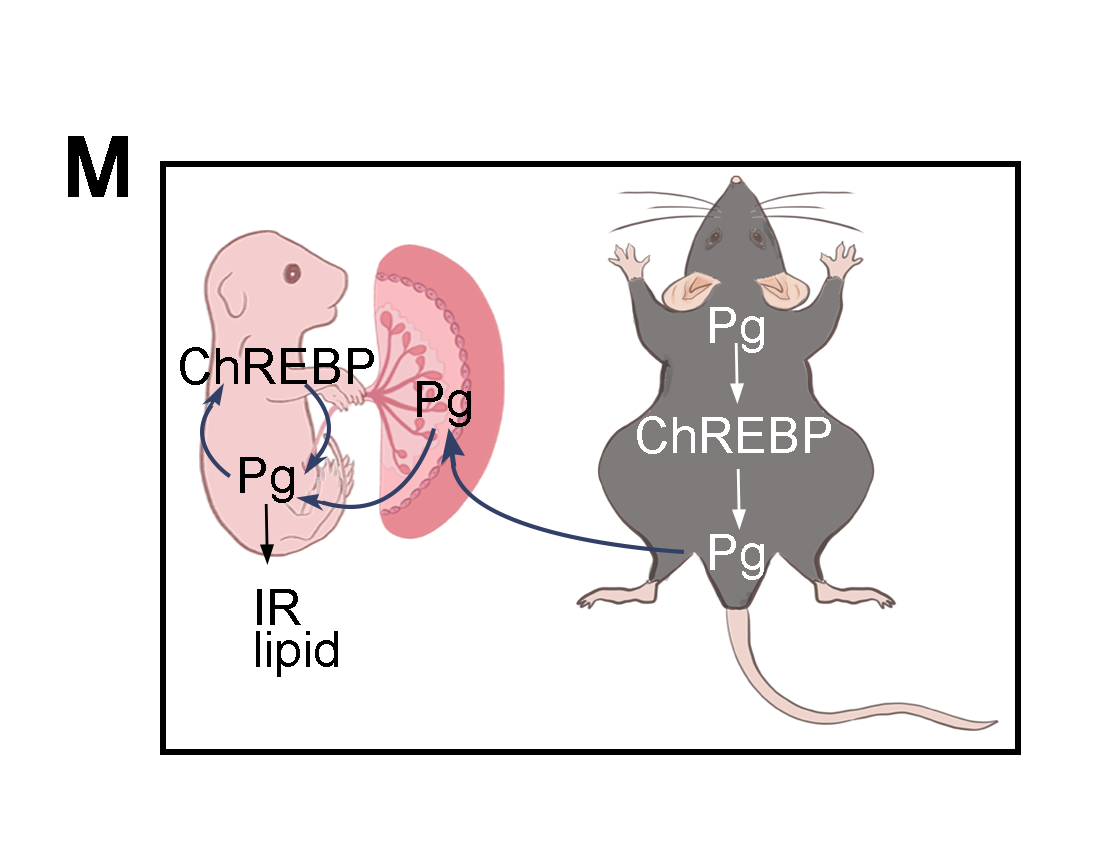

Supplement: Supplementary file 8 — Source data Fig. 7 [file 44319_2024_121_MOESM8_ESM.zip › Figure 7/M/Figure 7M.tif]
