# Supplementary material for: Liver ChREBP deficiency inhibits fructose-induced insulin resistance in pregnant mice and female offspring
Source: EMBO Rep. 2024 Mar 26;25(4):25. doi: 10.1038/s44319-024-00121-w (PMC11014959; doi:10.1038/s44319-024-00121-w)
Supplement: Supplementary file 4 — Source data Fig. 3 [file 44319_2024_121_MOESM4_ESM.zip › Figure 3/K/Results of statistical analysis of band density for Western blot.docx]

**Results of statistical analysis of band density for Western blot**

All the Western blot images were conducted analysis of band density, and normalized to the density of β-actin in the corresponding samples.

**Figure 3**

**Figure 3K:** (*P<0.05, **P<0.01, ***P<0.001 *vs.* CTRL, n = 3)

| **Genes** | **CTRL** | **PRO** |
| --- | --- | --- |
| ChREBP | 100±3 | 206±20* |
| PKLR | 100±4 | 151±2* |
| SCD1 | 100±4 | 160±12* |
