# Supplementary material for: Liver ChREBP deficiency inhibits fructose-induced insulin resistance in pregnant mice and female offspring
Source: EMBO Rep. 2024 Mar 26;25(4):25. doi: 10.1038/s44319-024-00121-w (PMC11014959; doi:10.1038/s44319-024-00121-w)
Supplement: Supplementary file 5 — Source data Fig. 4 [file 44319_2024_121_MOESM5_ESM.zip › Figure 4/E/Results of statistical analysis of band density for Western blot.docx]

**Results of statistical analysis of band density for Western blot**

All the Western blot images were conducted analysis of band density, and normalized to the density of β-actin in the corresponding samples.

**Figure 4**

**Figure 4E:** (*P<0.05, **P<0.01, ***P<0.001 *vs.* WPC, ^#^P<0.05, ^##^P<0.01, ^###^P<0.001 *vs.* WPF, ^^^P<0.05, ^^^^P<0.01, ^^^^^P<0.001 *vs.* KPC, n = 3)

| **Genes** | **WPC** | **WPF** | **KPC** | **KPF** |
| --- | --- | --- | --- | --- |
| APOB | 100±4 | 151±11** | 125±13 | 157±4 |
| MTTP | 100±6 | 216±3*** | 60±2* | 133±18^###^^^^ |
| ABCA1 | 100±2 | 267±3*** | 119±9 | 232±26^^^^^ |
| ABCG1 | 100±5 | 219±11** | 153±14 | 293±35^^^^^ |
