# Supplementary material for: Liver ChREBP deficiency inhibits fructose-induced insulin resistance in pregnant mice and female offspring
Source: EMBO Rep. 2024 Mar 26;25(4):25. doi: 10.1038/s44319-024-00121-w (PMC11014959; doi:10.1038/s44319-024-00121-w)
Supplement: Supplementary file 6 — Source data Fig. 5 [file 44319_2024_121_MOESM6_ESM.zip › Figure 5/H/Results of statistical analysis of band density for Western blot.docx]

**Results of statistical analysis of band density for Western blot**

All the Western blot images were conducted analysis of band density, and normalized to the density of β-actin in the corresponding samples.

**Figure 5**

**Figure 5H:** (*P<0.05, **P<0.01, *vs.* *Pparg*^flox/flox^-NP, n = 5)

| **Genes** | ***Pparg* ^flox/flox^-NP** | ***Pparg* ^flox/flox^-P** | ***Pparg* KO-NP** | ***Pparg* KO-P** |
| --- | --- | --- | --- | --- |
| ChREBP | 100±29 | 187±13*** | 30±7* | 60±10 |
