# Supplementary material for: Liver ChREBP deficiency inhibits fructose-induced insulin resistance in pregnant mice and female offspring
Source: EMBO Rep. 2024 Mar 26;25(4):25. doi: 10.1038/s44319-024-00121-w (PMC11014959; doi:10.1038/s44319-024-00121-w)
Supplement: Supplementary file 7 — Source data Fig. 6 [file 44319_2024_121_MOESM7_ESM.zip › Figure 6/I/Results of statistical analysis of band density for Western blot.docx]

**Results of statistical analysis of band density for Western blot**

All the Western blot images were conducted analysis of band density, and normalized to the density of β-actin in the corresponding samples.

**Figure 6**

**Figure 6I:** (*P<0.05, **P<0.01, ***P<0.001 *vs.* fPC, n = 3)

| **Genes** | **Female** | | **Male** | |
| --- | --- | --- | --- | --- |
|  | **fPC** | **fPF** | **fPC** | **fPF** |
| ChREBP | 100±10 | 166±16* | 80±6 | 84±16 |
| SCD1 | 100±20 | 159±1* | 89±1 | 121±9 |
| PKLR | 100±3 | 126±18 | 74±2 | 69±1 |
