# Supplementary material for: Liver ChREBP deficiency inhibits fructose-induced insulin resistance in pregnant mice and female offspring
Source: EMBO Rep. 2024 Mar 26;25(4):25. doi: 10.1038/s44319-024-00121-w (PMC11014959; doi:10.1038/s44319-024-00121-w)
Supplement: Supplementary file 8 — Source data Fig. 7 [file 44319_2024_121_MOESM8_ESM.zip › Figure 7/E/Results of statistical analysis of band density for Western blot.docx]

**Results of statistical analysis of band density for Western blot**

All the Western blot images were conducted analysis of band density, and normalized to the density of β-actin in the corresponding samples.

**Figure 7**

**Figure 7E:** (***P<0.001, *vs.* fWPC, n = 5)

| **Genes** | **fWPC** | **fWPF** | **fKPC-WT** | **fKPC-KO** | **fKPF-WT** | **fKPF-KO** |
| --- | --- | --- | --- | --- | --- | --- |
| p-IRS1 ^Ser1101^/IRES1 | 100±4 | 140±5*** | 114±11 | 94±4 | 92±4 | 83±12 |
| P-INSR ^Try1345^/ INSR | 100±22 | 45±5*** | 89±3 | 92±6 | 87±5 | 86±5 |
| p-AKT  ^Ser473^/ AKT | 100±21 | 41±2*** | 97±3 | 95±5 | 86±3 | 89±4 |
| p-GSK3β  ^Ser9^/ GSK3β | 100±19 | 49±4*** | 99±7 | 91±4 | 96±2 | 107±6 |
