# Supplementary material for: Liver ChREBP deficiency inhibits fructose-induced insulin resistance in pregnant mice and female offspring
Source: EMBO Rep. 2024 Mar 26;25(4):25. doi: 10.1038/s44319-024-00121-w (PMC11014959; doi:10.1038/s44319-024-00121-w)
Supplement: Supplementary file 8 — Source data Fig. 7 [file 44319_2024_121_MOESM8_ESM.zip › Figure 7/G/Results of statistical analysis of band density for Western blot.docx]

**Results of statistical analysis of band density for Western blot**

All the Western blot images were conducted analysis of band density, and normalized to the density of β-actin in the corresponding samples.

**Figure 7**

**Figure 7G:** (**P<0.01, ***P<0.001, *vs.* fWPC, n = 5)

| **Genes** | **fWPC** | **fWPF** | **fKPC-WT** | **fKPC-KO** | **fKPF-WT** | **fKPF-KO** |
| --- | --- | --- | --- | --- | --- | --- |
| ChREBP | 100±14 | 388±58** | 90±27 | 11±3*** | 91±13 | 15±3*** |
| PKLR | 100±14 | 174±22** | 85±29 | 60±9** | 86±5 | 27±4*** |
| SCD1 | 100±25 | 202±93** | 101±14 | 31±2*** | 87±5 | 26±3*** |
