# Supplementary material for: Liver ChREBP deficiency inhibits fructose-induced insulin resistance in pregnant mice and female offspring
Source: EMBO Rep. 2024 Mar 26;25(4):25. doi: 10.1038/s44319-024-00121-w (PMC11014959; doi:10.1038/s44319-024-00121-w)
Supplement: Supplementary file 9 — EV and Appendix Figures Source Data [file 44319_2024_121_MOESM9_ESM.zip › Appendix Figure S1/D/Results of statistical analysis of band density for Western blot.docx]

**Results of statistical analysis of band density for Western blot**

All the Western blot images were conducted analysis of band density, and normalized to the density of β-actin in the corresponding samples.

**Appendix Figure S1**

**Appendix Figure S1D:** (***P<0.001, *vs.* E0, n = 3)

| **Genes** | **E0** | **E2** | **E11** | **E17** |
| --- | --- | --- | --- | --- |
| p-IRS1 ^Ser1101^/IRES1 | 100±3 | 123±1*** | 182±5*** | 226±12*** |
| P-INSR ^Try1345^/ INSR | 100±4 | 48±8*** | 55±1*** | 35±2*** |
| p-AKT  ^Ser473^/ AKT | 100±1 | 71±8*** | 44±3*** | 30±3*** |
| p-GSK3β  ^Ser9^/ GSK3β | 100±8 | 91±5 | 49±5*** | 32±1*** |
