# Supplementary material for: Liver ChREBP deficiency inhibits fructose-induced insulin resistance in pregnant mice and female offspring
Source: EMBO Rep. 2024 Mar 26;25(4):25. doi: 10.1038/s44319-024-00121-w (PMC11014959; doi:10.1038/s44319-024-00121-w)
Supplement: Supplementary file 9 — EV and Appendix Figures Source Data [file 44319_2024_121_MOESM9_ESM.zip › Appendix Figure S1/E/Results of statistical analysis of band density for Western blot.docx]

**Results of statistical analysis of band density for Western blot**

All the Western blot images were conducted analysis of band density, and normalized to the density of β-actin in the corresponding samples.

**Appendix Figure S1**

**Appendix Figure S1E:** (*P<0.05, **P<0.01, ***P<0.001 *vs.* E0, n = 3)

| **Genes** | **WAT** | | | |
| --- | --- | --- | --- | --- |
|  | **E0** | **E2** | **E11** | **E17** |
| ChREBP | 100±16 | 138±23 | 89±16 | 55±13 |

| **Genes** | **BAT** | | | |
| --- | --- | --- | --- | --- |
|  | **E0** | **E2** | **E11** | **E17** |
| ChREBP | 100±15 | 126±12 | 155±28 | 247±46* |

| **Genes** | **Intestine** | | | |
| --- | --- | --- | --- | --- |
|  | **E0** | **E2** | **E11** | **E17** |
| ChREBP | 100±12 | 110±18 | 220±31*** | 137±29 |

| **Genes** | **Muscle** | | | |
| --- | --- | --- | --- | --- |
|  | **E0** | **E2** | **E11** | **E17** |
| ChREBP | 100±7 | 86±6 | 132±10* | 95±15 |

| **Genes** | **Pancreas** | | | |
| --- | --- | --- | --- | --- |
|  | **E0** | **E2** | **E11** | **E17** |
| ChREBP | 100±1 | 164±31 | 150±11* | 142±9 |
