# Supplementary material for: Liver ChREBP deficiency inhibits fructose-induced insulin resistance in pregnant mice and female offspring
Source: EMBO Rep. 2024 Mar 26;25(4):25. doi: 10.1038/s44319-024-00121-w (PMC11014959; doi:10.1038/s44319-024-00121-w)
Supplement: Supplementary file 9 — EV and Appendix Figures Source Data [file 44319_2024_121_MOESM9_ESM.zip › Appendix Figure S2/A/Results of statistical analysis of band density for Western blot.docx]

**Results of statistical analysis of band density for Western blot**

All the Western blot images were conducted analysis of band density, and normalized to the density of β-actin in the corresponding samples.

**Appendix Figure S2**

**Appendix Figure S2A:** (***P<0.001, *vs.* E0, n = 3)

| **Genes** | **E0** | **E2** | **E11** | **E17** |
| --- | --- | --- | --- | --- |
| SCD1 | 100±6 | 288±31*** | 380±8*** | 410±22*** |
| PKLR | 100±5 | 254±4*** | 278±12*** | 300±12*** |
