# Supplementary material for: Liver ChREBP deficiency inhibits fructose-induced insulin resistance in pregnant mice and female offspring
Source: EMBO Rep. 2024 Mar 26;25(4):25. doi: 10.1038/s44319-024-00121-w (PMC11014959; doi:10.1038/s44319-024-00121-w)
Supplement: Supplementary file 9 — EV and Appendix Figures Source Data [file 44319_2024_121_MOESM9_ESM.zip › Appendix Figure S4/C/Results of statistical analysis of band density for Western blot.docx]

**Results of statistical analysis of band density for Western blot**

All the Western blot images were conducted analysis of band density, and normalized to the density of β-actin in the corresponding samples.

**Appendix Figure S4**

**Appendix Figure S4C:** (*P<0.05, **P<0.01, ***P<0.001 *vs.* Progesterone dose-0, n = 3)

| **Genes**  Estradiol  dose | **Primary hepatocytes** | | | | | |
| --- | --- | --- | --- | --- | --- | --- |
|  | **0** | **0.1** | **0.5** | **1** | **5** | **10** |
| ChREBP | 100±5 | 90±1 | 72±3** | 60±1*** | 86±1 | 87±1 |
