# Supplementary material for: Liver ChREBP deficiency inhibits fructose-induced insulin resistance in pregnant mice and female offspring
Source: EMBO Rep. 2024 Mar 26;25(4):25. doi: 10.1038/s44319-024-00121-w (PMC11014959; doi:10.1038/s44319-024-00121-w)
Supplement: Supplementary file 9 — EV and Appendix Figures Source Data [file 44319_2024_121_MOESM9_ESM.zip › Appendix Figure S7/B/Results of statistical analysis of band density for Western blot.docx]

**Results of statistical analysis of band density for Western blot**

All the Western blot images were conducted analysis of band density, and normalized to the density of β-actin in the corresponding samples.

**Appendix Figure S7**

**Appendix Figure S3B:** (*P<0.05, **P<0.01, ***P<0.001, *vs.* Ctrl, n = 4)

| **Progesterone** | **Primary hepatocytes** | | | | | |
| --- | --- | --- | --- | --- | --- | --- |
|  | **0** | **25** | **100** | **0** | **25** | **100** |
| **Mifepristone** | **0** | **0** | **0** | **100** | **100** | **100** |
| ChREBP | 100±3 | 142±3*** | 149±7*** | 102±3 | 95±2 | 85±1 |
| PGR-A | 100±1 | 99±1 | 108±1 | 49±3*** | 39±1*** | 32±1*** |
| PGR-B | 100±5 | 101±6 | 126±4 | 40±1*** | 40±1*** | 42±2*** |
