# Supplementary material for: Liver ChREBP deficiency inhibits fructose-induced insulin resistance in pregnant mice and female offspring
Source: EMBO Rep. 2024 Mar 26;25(4):25. doi: 10.1038/s44319-024-00121-w (PMC11014959; doi:10.1038/s44319-024-00121-w)
Supplement: Supplementary file 9 — EV and Appendix Figures Source Data [file 44319_2024_121_MOESM9_ESM.zip › Appendix Figure S7/C/Results of statistical analysis of band density for Western blot.docx]

**Results of statistical analysis of band density for Western blot**

All the Western blot images were conducted analysis of band density, and normalized to the density of β-actin in the corresponding samples.

**Appendix Figure S7**

**Appendix Figure S3C:** (*P<0.05, *vs.* Ctrl, n = 4)

| **Genes** | ***Pparg*** ^flox/flox^ | |
| --- | --- | --- |
|  | **Ctrl** | **Rosi** |
| ChREBP | 100±5 | 144±11* |
| PPARγ | 100±8 | 129±4* |
