# Supplementary material for: Liver ChREBP deficiency inhibits fructose-induced insulin resistance in pregnant mice and female offspring
Source: EMBO Rep. 2024 Mar 26;25(4):25. doi: 10.1038/s44319-024-00121-w (PMC11014959; doi:10.1038/s44319-024-00121-w)
Supplement: Supplementary file 9 — EV and Appendix Figures Source Data [file 44319_2024_121_MOESM9_ESM.zip › Appendix Figure S10/Results of statistical analysis of band density for Western blot.docx]

**Results of statistical analysis of band density for Western blot**

All the Western blot images were conducted analysis of band density, and normalized to the density of β-actin in the corresponding samples.

**Appendix Figure S10**

**Appendix Figure S10:** (*P<0.05, **P<0.01, ***P<0.001 *vs.* EV-Progesterone-insulin-, ^#^P<0.05, ^##^P<0.01, ^###^P<0.001 *vs.* plRES-ChREBP-Progesterone-insulin, n = 3)

| **HepG2 cells** | | | | | | | | |
| --- | --- | --- | --- | --- | --- | --- | --- | --- |
| Pro | **-** | **+** | **-** | **+** | **-** | **+** | **-** | **+** |
| insulin | **-** | **-** | **+** | **+** | **-** | **-** | **+** | **+** |
| **Gene** | **EV** | | | | **plRES-ChREBP** | | | |
| p-AKT ^Ser473^/AKT | 100±4 | 94±6 | 188±4* | 319±79*** | 379±7*** | 479±41^###^ | 739±10^###^ | 785±34^###^ |
| ChREBP | 100±1 | 174±6* | 216±6* | 204±8* | 311±7*** | 390±16^###^ | 405±17^###^ | 618±18^###^ |
