# Supplementary material for: Liver ChREBP deficiency inhibits fructose-induced insulin resistance in pregnant mice and female offspring
Source: EMBO Rep. 2024 Mar 26;25(4):25. doi: 10.1038/s44319-024-00121-w (PMC11014959; doi:10.1038/s44319-024-00121-w)
Supplement: Supplementary file 9 — EV and Appendix Figures Source Data [file 44319_2024_121_MOESM9_ESM.zip › Figure EV2/D/Results of statistical analysis of band density for Western blot.docx]

**Results of statistical analysis of band density for Western blot**

All the Western blot images were conducted analysis of band density, and normalized to the density of β-actin in the corresponding samples.

**Figure EV2**

**Figure EV2D:** (n = 5)

| **Genes** | **fWPC** | **fWPF** | **fKPC-WT** | **fKPC-KO** | **fKPF-WT** | **fKPF-KO** |
| --- | --- | --- | --- | --- | --- | --- |
| p-IRS1 ^Ser1101^/IRES1 | 100±11 | 86±3 | 79±12 | 78±7 | 85±7 | 78±5 |
| P-INSR ^Try1345^/ INSR | 100±9 | 93±10 | 86±3 | 80±3 | 79±9 | 78±22 |
| p-AKT  ^Ser473^/ AKT | 100±18 | 113±8 | 83±4 | 93±6 | 88±10 | 82±6 |
| p-GSK3β  ^Ser9^/ GSK3β | 100±10 | 196±47 | 226±19 | 137±20 | 119±8 | 170±29 |
