# Supplementary material for: Liver ChREBP deficiency inhibits fructose-induced insulin resistance in pregnant mice and female offspring
Source: EMBO Rep. 2024 Mar 26;25(4):25. doi: 10.1038/s44319-024-00121-w (PMC11014959; doi:10.1038/s44319-024-00121-w)
Supplement: Supplementary file 9 — EV and Appendix Figures Source Data [file 44319_2024_121_MOESM9_ESM.zip › Figure EV2/F/Results of statistical analysis of band density for Western blot.docx]

**Results of statistical analysis of band density for Western blot**

All the Western blot images were conducted analysis of band density, and normalized to the density of β-actin in the corresponding samples.

**EV 2**

**EV 2F:** (**P<0.01, ***P<0.001, *vs.* fWPC, n = 5)

| **Genes** | **fWPC** | **fWPF** | **fKPC-WT** | **fKPC-KO** | **fKPF-WT** | **fKPF-KO** |
| --- | --- | --- | --- | --- | --- | --- |
| ChREBP | 100±14 | 113±17 | 106±28 | 15±7*** | 106±7 | 13±9*** |
| PKLR | 100±21 | 133±27 | 126±20 | 63±25* | 103±12 | 74±9* |
| SCD1 | 100±6 | 111±19 | 121±10 | 63±7** | 124±17 | 55±7** |
