# Supplementary material for: Liver ChREBP deficiency inhibits fructose-induced insulin resistance in pregnant mice and female offspring
Source: EMBO Rep. 2024 Mar 26;25(4):25. doi: 10.1038/s44319-024-00121-w (PMC11014959; doi:10.1038/s44319-024-00121-w)
Supplement: Supplementary file 9 — EV and Appendix Figures Source Data [file 44319_2024_121_MOESM9_ESM.zip › Figure EV1/D/Results of statistical analysis of band density for Western blot.docx]

**Results of statistical analysis of band density for Western blot**

All the Western blot images were conducted analysis of band density, and normalized to the density of β-actin in the corresponding samples.

**Figure EV1**

**Figure EV1D:** (*P<0.05, **P<0.01, ***P<0.001 *vs.* CC, ^#^P<0.05, ^##^P<0.01, ^###^P<0.001 *vs.* CF, ^^^P<0.05, ^^^^P<0.01, ^^^^^P<0.001 *vs.* PC, n = 3)

| **Genes** | **CC** | **CF** | **PC** | **PF** |
| --- | --- | --- | --- | --- |
| p-IRS1 ^Ser1101^/IRES1 | 100±5 | 132±4*** | 204±6*** | 370±17^###^^^^ |
| P-INSR ^Try1345^/ INSR | 100±10 | 79±5* | 67±3*** | 38±2^###^^^ |
| p-AKT  ^Ser473^/ AKT | 100±7 | 72±9* | 86±13* | 47±1^###^^^^ |
| p-GSK3β  ^Ser9^/ GSK3β | 100±5 | 69±7*** | 52±2*** | 25±2^###^^^^ |
