# Supplementary material for: Liver ChREBP deficiency inhibits fructose-induced insulin resistance in pregnant mice and female offspring
Source: EMBO Rep. 2024 Mar 26;25(4):25. doi: 10.1038/s44319-024-00121-w (PMC11014959; doi:10.1038/s44319-024-00121-w)
Supplement: Supplementary file 11 — Expanded View Figures [file 44319_2024_121_MOESM11_ESM.pdf]

Expanded View Figures

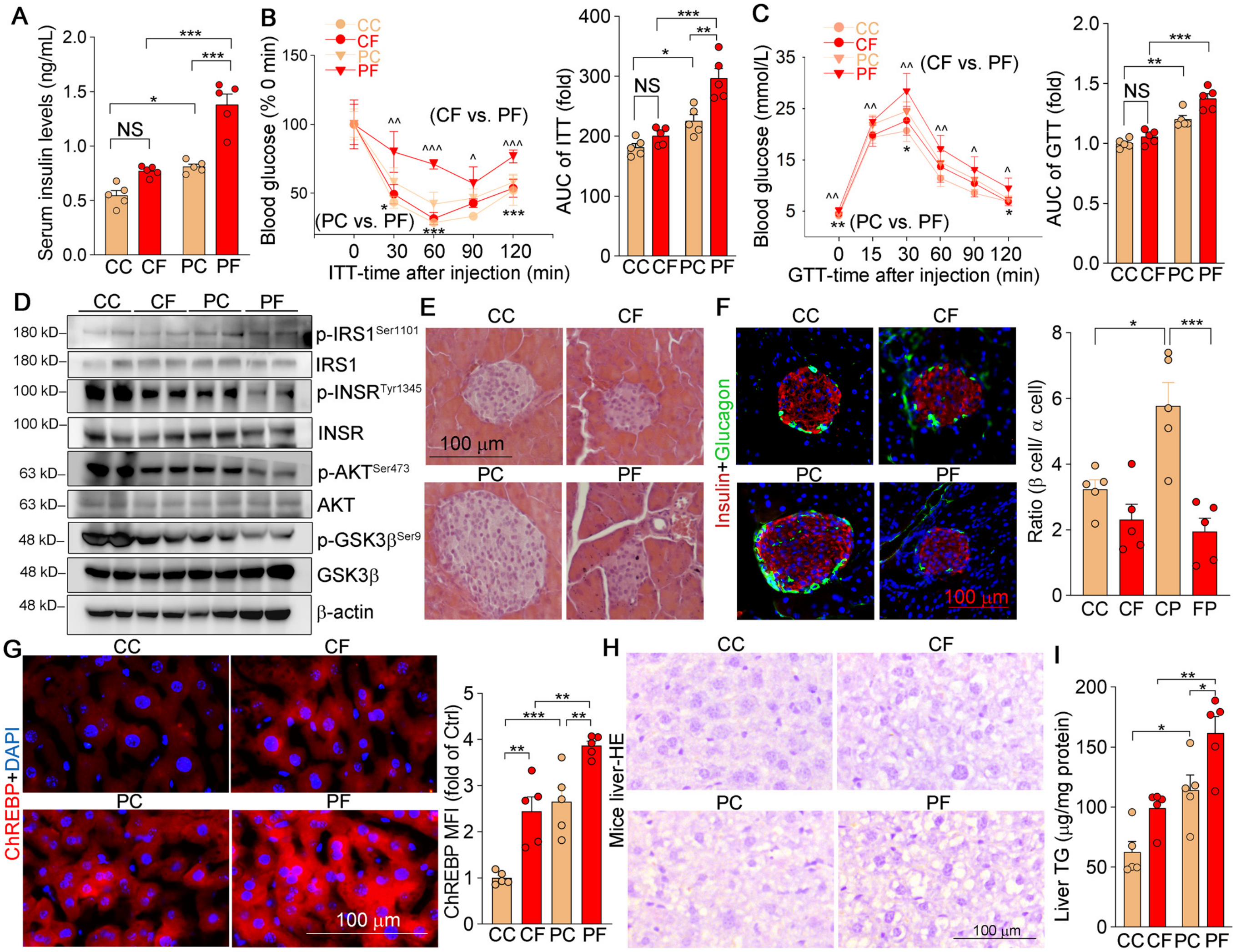

Figure EV1. HFrD aggravates glucose and lipid metabolism disorders in maternal mice.

The mice in Fig. 1E were used to complete following assays: (**A**–**C**) Serum insulin level (**A**), ITT (**B**) or GTT (**C**) assay at the E14 or E15, and quantitation of areas under curves (AUC). (**D**) Expression of protein related to insulin signaling pathway in mouse liver was determined by Western blot. (**E**) HE staining of mice pancreas sections. (**F**) Immunofluorescent staining with anti-glucagon (green) or anti-insulin (red) antibodies of mice pancreas sections, and the ratio of beta cell area (insulin-positive) to alpha cell area (glucagon-positive) was calculated, $n = 5$. (**G**) The protein expression of ChREBP was determined by immunofluorescent staining with quantification of the mean immunofluorescence intensity (MFI) of images. (**H**) HE staining of liver sections. (**I**) triglyceride (TG) quantification in the liver. $n = 5$. Data information: All graphs are represented as Mean ± SEM, *n*: biological replicates. Two-way ANOVA followed by Tukey's multiple comparisons test was used. *$P < 0.05$, **$P < 0.01$, ***$P < 0.001$, NS: no significance vs. indicated group. Source data are available online for this figure.

 © The Author(s)

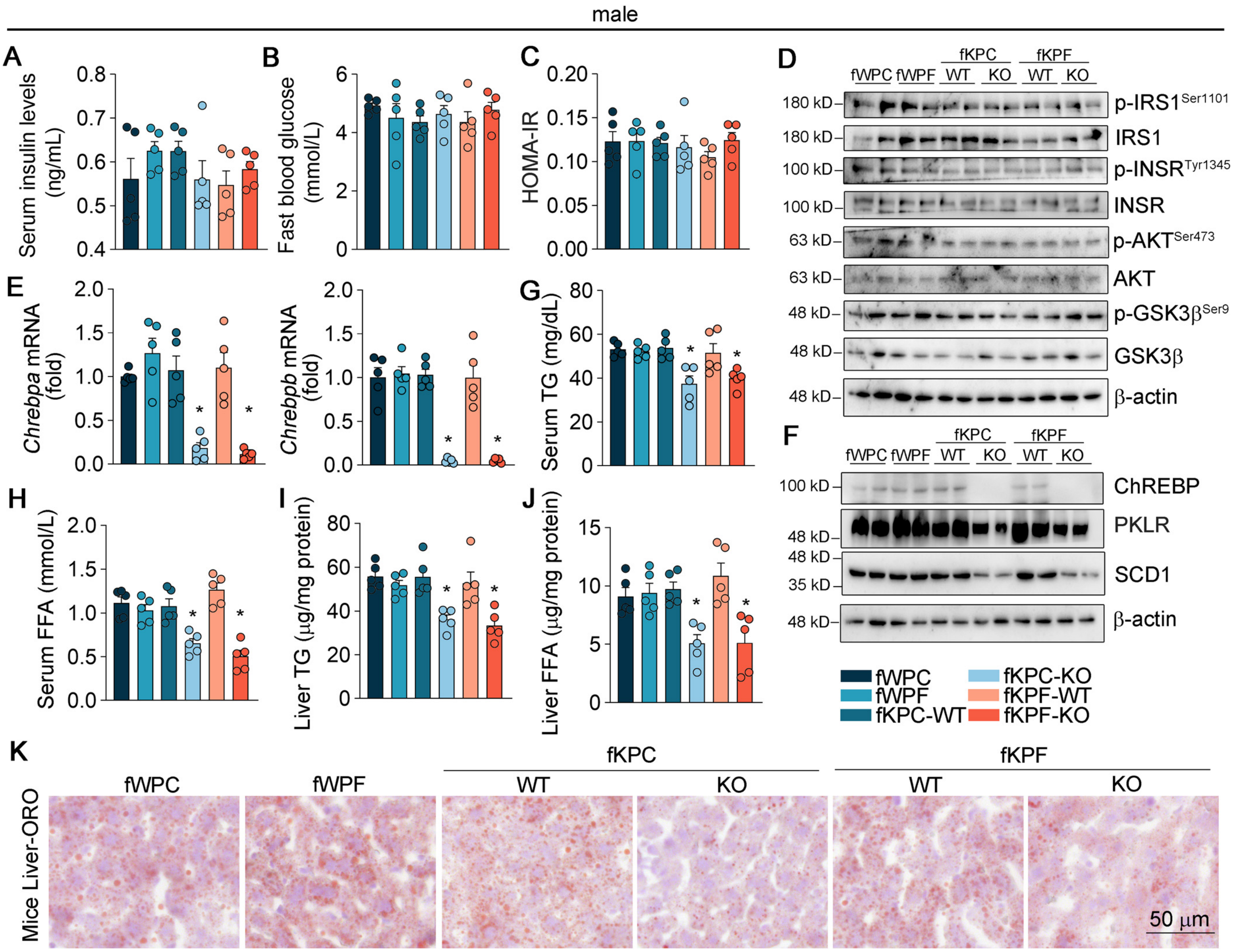

Figure EV2. Hepatic ChREBP deficiency did not improve HFrD-impaired the glucose and lipid homeostasis of male offspring.

The male offspring in Fig. 7A were used to complete following assays: (**A–C**) Serum insulin levels (**A**), fast blood glucose (**B**) and HOMA-IR index (**C**) of male offspring. (**D**,**F**) Expression of protein related to insulin signaling pathway (**D**) and ChREBP and its downstream target protein (**F**) was determined by Western blot in the liver from male offspring. (**E**) Expression of *Chrebpa* and *Chrebpb* mRNA in the liver from male offspring was determined by qRT-PCR. (**G–J**) The serum (**G** and **H**) and liver (**I** and **J**) were conducted triglyceride (TG) and free fatty acid (FFA) quantitative analysis. (**I**) Oil Red O (ORO) staining of liver sections (**K**). $n = 5$. Data information: All graphs are represented as Mean ± SEM, *n*: biological replicates. One-way ANOVA followed by Tukey's multiple comparisons test was used. *$P < 0.05$ vs. fWPC group. Source data are available online for this figure.

© The Author(s)
